# From amides to thioamides: understanding enhanced anion binding in acyclic receptors

**DOI:** 10.1039/d5ra08433d

**Published:** 2025-11-28

**Authors:** Nasim Akhtar, Siebe Lekanne Deprez, Senuri G. Jayawardana, Macallister Davis, Célia Fonseca Guerra, Víctor García-López

**Affiliations:** a Department of Chemistry, Louisiana State University Baton Rouge LA 70803 USA vglopez@lsu.edu; b Department of Chemistry and Pharmaceutical Sciences, Amsterdam Institute for Molecular and Life Sciences (AIMMS), Vrije Universiteit Amsterdam De Boelelaan 1108 1081 HZ Amsterdam The Netherlands c.fonsecaguerra@vu.nl

## Abstract

We synthesized acyclic amide and thioamide-based receptors and evaluated their anion binding efficacy experimentally and computationally. Our study shows the receptors adopt favorable conformations upon chloride binding and confirms that the stronger NH donor ability of thioamides arises from sulfur's larger size relative to oxygen. Moreover, the anion size dictates selectivity.

## Abstract 2

Anion selectivity of amide- and thioamide-based receptors determined by conformation and hydrogen bonds.

## Graphical abstract

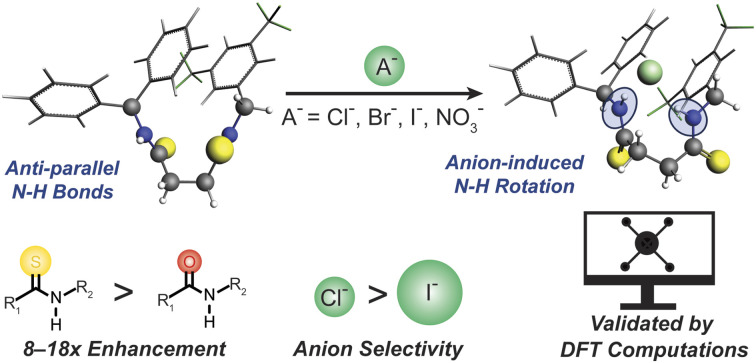

## Introduction

Synthetic anion receptors^1^ are widely used in catalysis,^2^ sensing,^3^ and bioimaging,^4^ and they also offer promising opportunities for therapeutic applications by transporting anions across cell membranes.^5–7^ Most synthetic anion receptors rely on hydrogen bonding,^8–10^ often using N–H donor groups such as pyrroles,^11^ indoles,^12^ squaramides,^13^ ureas,^14^ and amides.^15^ Many of these receptors are highly preorganized,^16–18^ which boosts anion binding efficiency but usually requires more demanding synthesis. Some simpler acyclic systems incorporate rigid motifs to help orient N–H groups,^19–21^ while a few utilize anion-driven reorganization,^22–24^ in which the target anion itself induces a favorable binding conformation.

A notable modification to amides and ureas to enhance their hydrogen-bond donor strength is the replacement of oxygen with sulfur to obtain thioureas and thioamides.^14,25^ Although this strategy had long been recognized experimentally, its fundamental electronic origin has only recently been clarified through theoretical work.^26–28^ These studies demonstrated that the larger size of the S atom in thioureas and thioamides is responsible for the enhanced acidity of the N–H groups, rather than electronegativity or polarizability effects. This theoretical framework was primarily investigated in the context of thioureas and thioamides binding carbonyl groups for organocatalysis and supramolecular materials.^27–29^ However, a comparable study aimed at understanding the role of sulfur *versus* oxygen in thioamides for anion binding and selectivity has so far remained elusive.

Furthermore, while thioureas have been extensively studied as anion receptors, thioamide-based systems, especially acyclic ones, remain less explored, partly because effective binding requires the cooperative orientation of two N–H groups toward the same anion.

We designed a series of synthetically simple acyclic thio(amide)-based receptors that adopt a favorable conformation upon anion binding, allowing cooperative engagement of the two N–H groups. Despite lacking permanent preorganization, these receptors display clear binding preferences for Cl^−^ anions over nitrate and other halides. Notably, incorporation of thioamides enhances anion binding by about eight to eighteen-fold compared to the corresponding amides. Additionally, we present a theoretical framework that provides a mechanistic understanding of the steric, orbital, and electrostatic contributions governing the (thio)amide–anion interactions.

## Result and discussion

### Design and synthesis

**Fig. 1:**
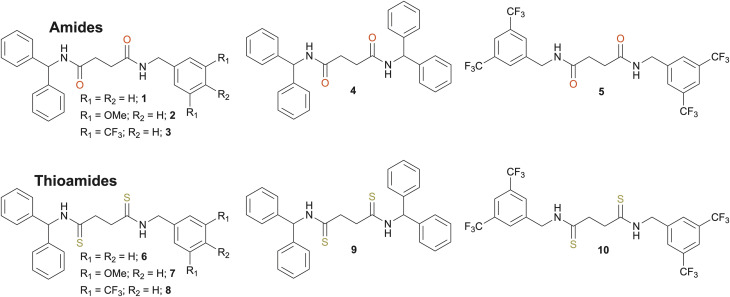
Structures of amide-based receptors 1–5 and thioamide-based receptors 6–10.

We designed ten acyclic receptors, five containing amides (1–5) and five containing the corresponding thioamide analogs (6–10) (Fig. 1). The compounds contain central (thio)amide groups linked by an ethylene spacer and capped with diphenylmethyl or benzyl groups bearing different substituents.

**Scheme 1:**
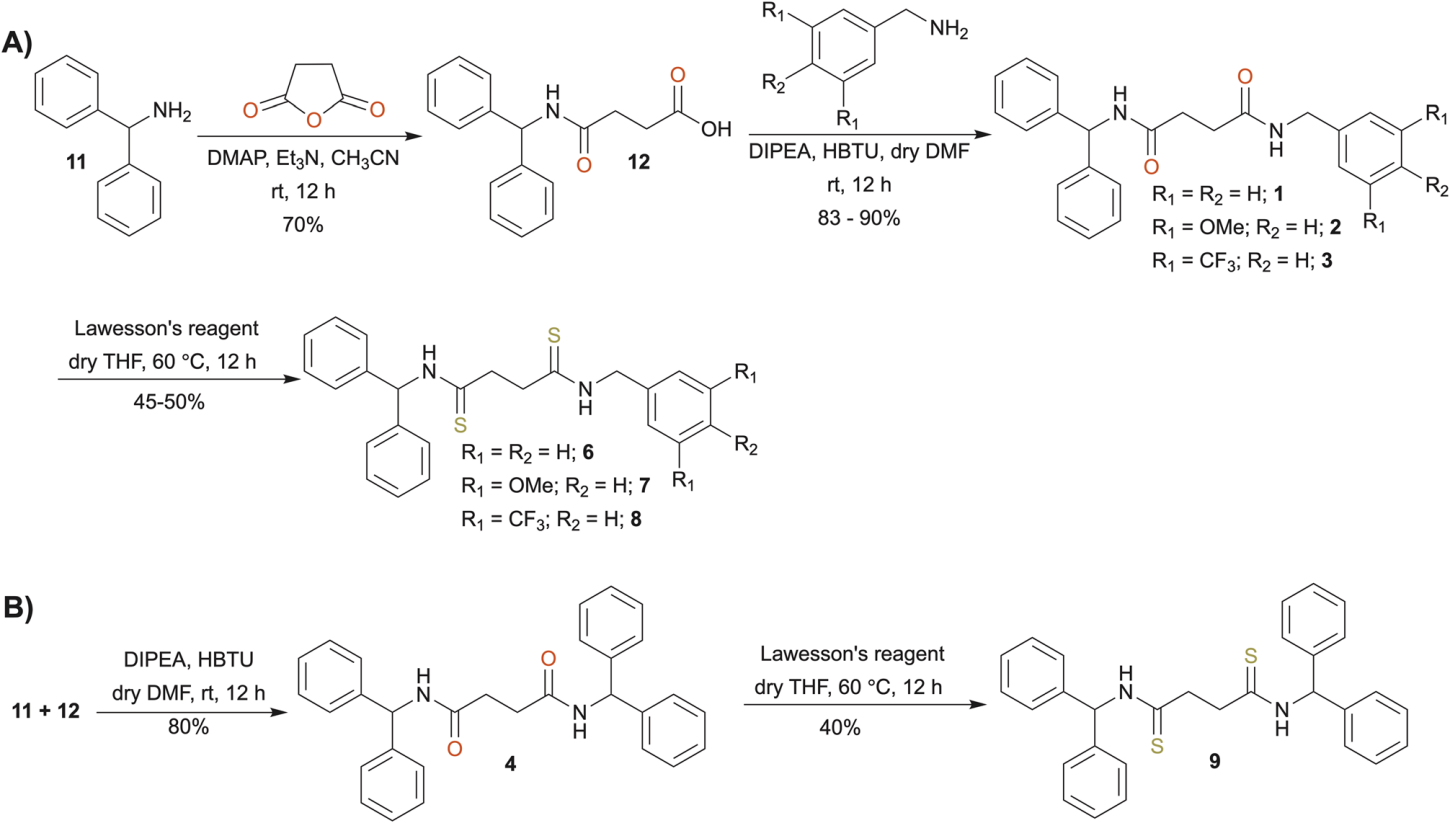
Synthesis of (A) receptors 1–3 and 6–8, and (B) receptors 4 and 9.

**Scheme 2:**
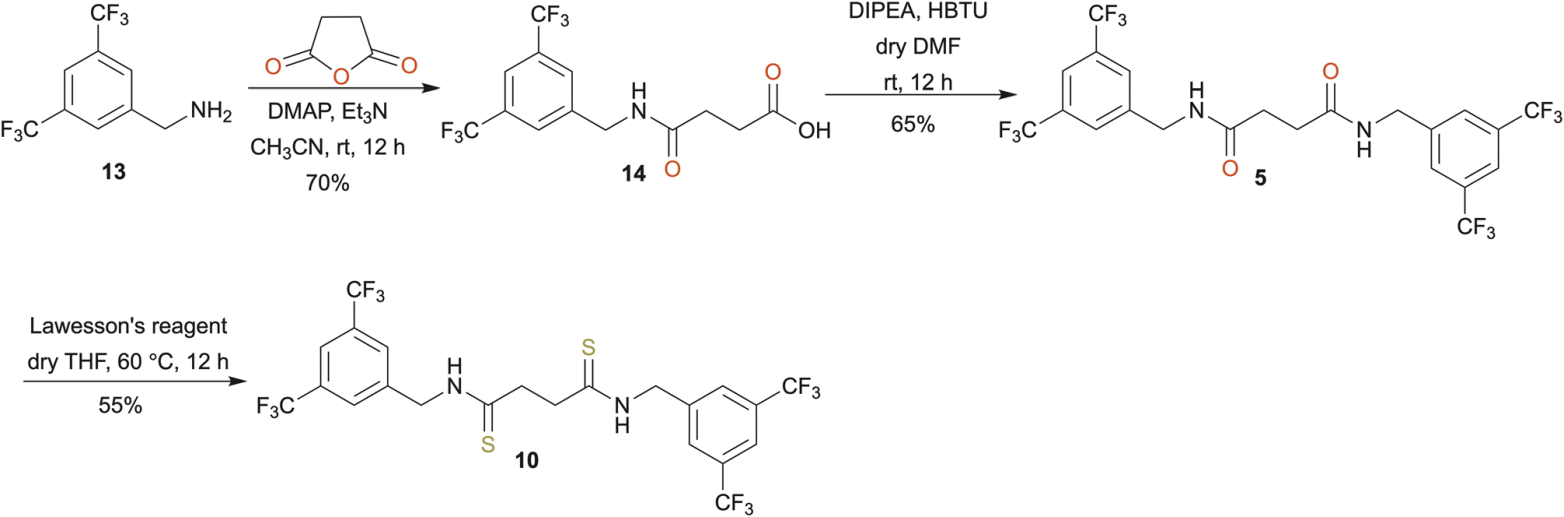
Synthesis of receptors 5 and 10.

The synthesis initiated by reacting diphenylmethanamine (11) and succinic anhydride to obtain the intermediate 4-(benzhydrylamino)-4-oxobutanoic acid (12) (Scheme 1). The reaction of 12 with the corresponding benzylamine derivatives gave the desired amide-based anion receptors (1–4). Afterwards, Lawesson's reagent was used to obtain the thioamide-based anion receptors (6–9). We also synthesized compounds 5 and 10 containing 3,5-bis(trifluoromethyl)benzyl groups (Scheme 2) using similar synthetic routes. Specifically, 3,5-bis(trifluoromethyl)benzyl amine (13) reacts with succinic anhydride to obtain compound 14, which then reacts with another 3,5-bis(trifluoromethyl)benzyl amine to obtain amide-based receptor 5. Reaction with Lawesson's reagent gave thioamide-based receptor 10. The synthetic protocols and structural characterization are described in the SI (Sections S1–S3).

### Anion recognition

**Fig. 2:**
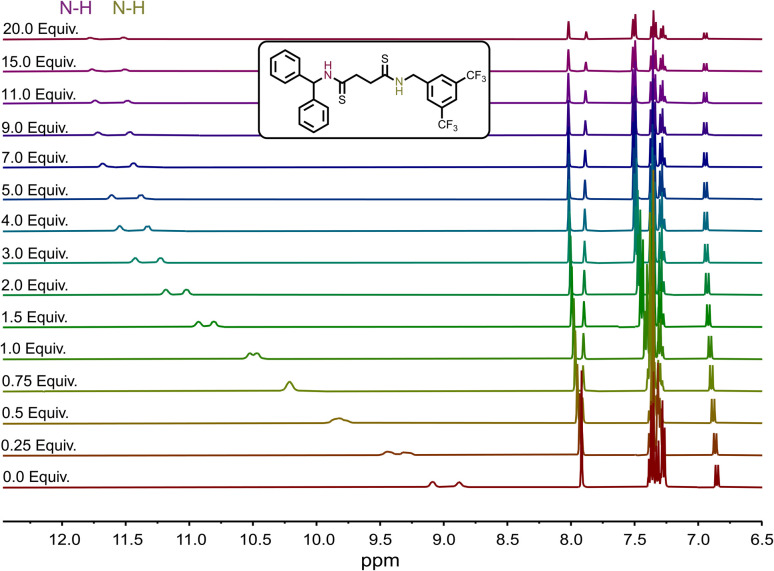
^1^H NMR spectra showing the chemical shifts of the N–H signals during the titration of 8 with TBACl in acetonitrile-d_3_.

We assigned the chemical shifts of the N–H protons for receptors 3 and 8 through comprehensive 2D NMR analyses (Fig. S1–S4). We then conducted ^1^H NMR titrations in acetonitrile-d_3_ to assess chloride binding. A 5 mM solution of each receptor was titrated with increasing concentrations of tetrabutylammonium chloride (TBACl). The N–H proton signals shifted downfield as the equivalents of TBACl increased, indicating the binding of Cl^−^ anions (Fig. 2). Amide-based receptors 1–5 displayed relatively weak binding, with association constants ranging from 30 to 59 M^−1^ (Table 1).

**Table 1:** Binding constants (*K*_a_) for Cl^−^ with receptors in acetonitrile-*d*_3_ obtained from NMR titration data (1 : 1 binding model)

Compound	*K*_a_ (M^−1^)	Compound	*K*_a_ (M^−1^)
1	30 ± 3	6	530 ± 50
2	39 ± 4	7	300 ± 30
3	59 ± 6	8	540 ± 50
4	32 ± 3	9	a
5	50 ± 5	10	510 ± 50

a Low solubility of compound 9 hindered the titration.

In contrast, thioamide-based receptors 6–10 bound chloride much more strongly, with constants ranging from 300 to 540 M^−1^. Compound 8 exhibited the highest affinity (540 M^−1^), representing a ninefold enhancement over its amide analog (3). Receptor 6 showed the most significant relative improvement, with a 18-fold increase, while receptors 10 and 7 exhibited 10- and 8-fold increases, respectively. Due to poor solubility in acetonitrile (ACN), receptor 9 could not be titrated, and its binding constant could not be determined. Within the thioamide series, compounds 6, 8, and 10 exhibited comparable binding constants, indicating that the substituents in the peripheral aromatic groups exert similar effects in these receptors. In contrast, compound 7 showed a significantly lower binding constant. We propose that the aromatic end groups participate in π–π stacking interactions that help define a cavity for chloride binding. In this context, the electron-rich ring bearing methoxy substituents in compound 7 likely weakens these interactions, leading to a detrimental effect compared to the other substituents and resulting in reduced binding affinity.^30^

The binding constants reported in Table 1 were obtained using a 1 : 1 binding model. However, the inherent flexibility of the receptors could, in principle, allow for alternative binding stoichiometries. To assess this possibility, we evaluated their binding behavior using different host–guest models. All receptors were analyzed with 1 : 1, 1 : 2, and 2 : 1 models within the Bindfit v0.5 platform (Nelder–Mead fitting method). The best fit was obtained for the 1 : 1 model, as indicated by the lowest fitting error. For example, the 1 : 2 model resulted in significantly higher errors, while the 2 : 1 model provided a reasonable fit but consistently with larger errors than the 1 : 1 model. Comparative fitting results for all receptors, along with detailed plots for receptors 3 and 8, are provided in the SI (Tables S2 and S3, Section S5).

Further, compound 8 was selected for anion-selectivity studies, as it exhibited the highest chloride binding affinity. ^1^H NMR titrations with TBABr, TBAI, and TBANO_3_ revealed a clear halide preference, with the selectivity order Cl^−^ > Br^−^ > NO_3_^−^ > I^−^ (Table 2, and Fig. S27–S32). The binding constant for Cl^−^ was 540 M^−1^, which dropped nearly fivefold for Br^−^ (112 M^−1^) and further to 32 M^−1^ for NO_3_^−^ and 15 M^−1^ for I^−^. This trend indicates a strong preference for chloride over the larger and less basic anions.

**Table 2:** Binding constants of receptor 8 for different anions in acetonitrile-*d*_3_ obtained from NMR titration data (1 : 1 binding model)

*K*_a_ (M^−1^)			
Cl^−^	Br^−^	I^−^	NO_3_^−^
540 ± 54	112 ± 11	15 ± 2	32 ± 3

### Mass spectrometric and crystallographic analysis

We sought to gain further insight into the receptor binding modes using X-ray crystallography and mass spectrometry. Receptors 3, 4, 8, and 9 were analyzed in the presence of TBACl by electrospray ionization mass spectrometry (ESI-MS). The ESI-MS data indicated the formation of chloride-bound complexes for all receptors, with clear peaks corresponding to the chloride-associated species (Fig. S33–S36). These results further support the anion-binding capability of both the amide- and thioamide-based receptors.

**Fig. 3:**
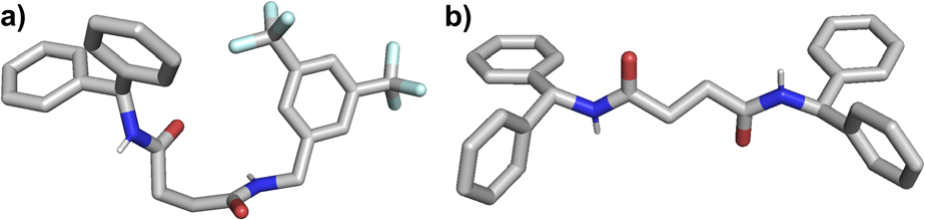
Crystal structures of (a) receptor 3 and (b) receptor 4.

We attempted to obtain single-crystal structures of the receptors in their chloride-bound forms. Despite numerous attempts, single crystals of the chloride complexes could not be obtained. However, using a slow evaporation method in acetonitrile, we successfully grew single crystals of the unbound amide receptors 3 and 4 (Fig. 3, S37 and S38). In these structures, the N–H bonds are oriented in opposite directions, revealing a flexible conformation that lacks a defined binding cavity in the absence of anions. This structural observation supports our hypothesis that, without bound anions, the amide receptors are not preorganized to form a cavity suitable for high-affinity binding.

### Computational studies

**Fig. 4:**
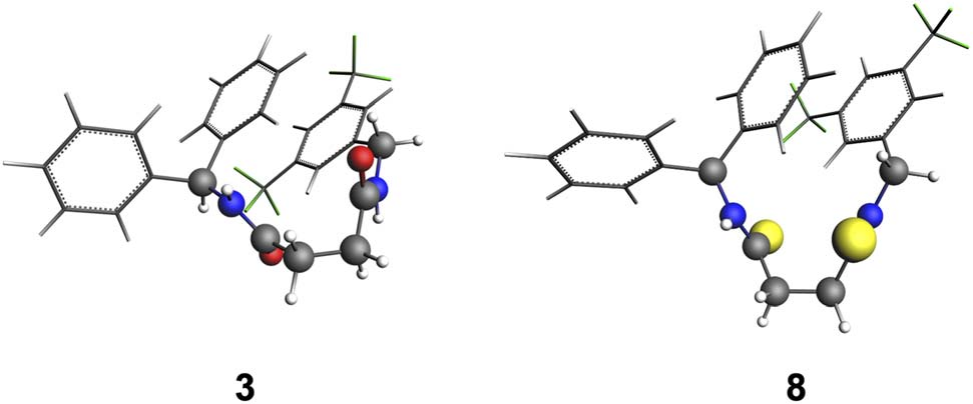
Global minima structures for receptors 3 and 8 (see Fig. S35 for less stable conformers), computed at COSMO(ACN)-ZORA-BLYP-D3(BJ)/TZ2P.

We conducted a computational study to help rationalize the experimental results, using compounds 3 and 8 as model systems. The crystal structure of 3 served as an input structure for determining the relevant conformers of 3 and 8, of which the oxygen atoms were replaced by sulfur for the latter (see Section S8). Fig. 4 depicts the most stable conformers of 3 and 8, which show two distinct geometric features (see Fig. S39 for less stable conformers). The first feature of 3 and 8 is the stacked phenyl ring, originating from the phenyl groups at both ends of the receptor, and the second feature is the anti-parallel orientation of the amide groups. These features are relevant for predicting how an anion binds to the receptor, as the amide groups should be oriented in a parallel manner to facilitate a double hydrogen bond with the anion (*vide infra*).

**Fig. 5:**
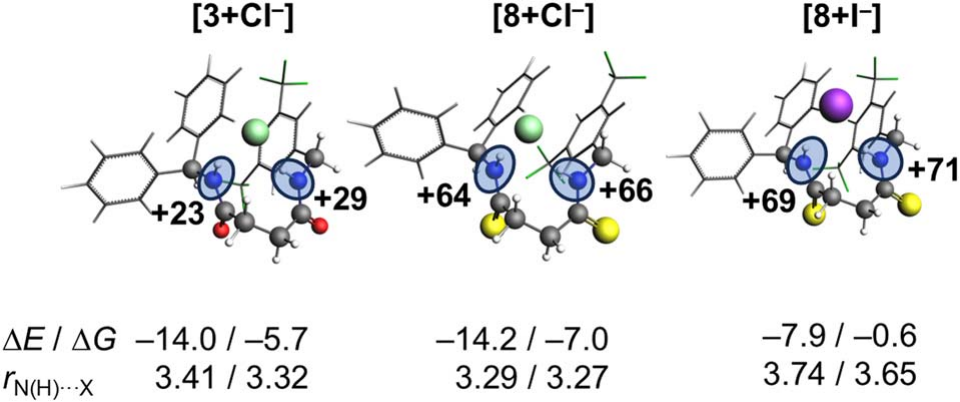
Anion-receptor complexes [3+Cl^−^], [8+Cl^−^] and [8+I^−^]. The (Gibbs free) energy in acetonitrile is reported in kcal mol^−1^. The hydrogen bond distances *r*_N(H)⋯X_ are reported in Å and the charges of the NH groups in milli-electrons, computed at COSMO(ACN)-ZORA-BLYP-D3(BJ)/TZ2P.

We first consider the difference in varying the chalcogen of the receptor on the binding affinities between the receptor and the anion Cl^−^. Our computations reveal that the anion binds to the receptor through two hydrogen bonds with both NH hydrogen-bond donors simultaneously, which is shown in Fig. 5 (weaker coordination modes between receptor and anion are shown in Fig. S40).

Both the gas phase and in acetonitrile calculations show that the (Gibbs free) energy trends are in line with experiments. For example, the thioamide-based complex [8+Cl^−^] is more stabilized in acetonitrile than [3+Cl^−^] in terms of energies (−14.2 *vs.* −14.0 kcal mol^−1^) and Gibbs free energies (−7.0 *vs.* −5.7 kcal mol^−1^).

**Fig. 6:**
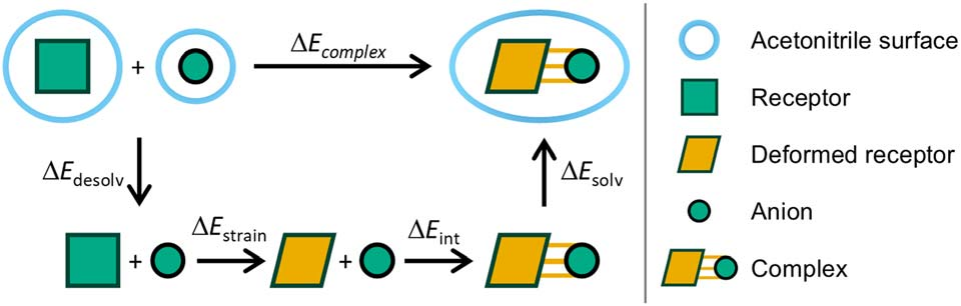
Schematic representation of the coordination strength partitioning in solution. Since the anion is a single atom, no geometric deformation occurs for the anion.

To further understand why Cl^−^ binds stronger to 8 than 3, the activation strain model (ASM) and energy decomposition analysis (EDA) schemes have been employed, which divide the complexation energy into the energy required to deform the monomer and the actual interaction with the anion (see Section S9.1). The energy terms have been adapted to study the role of the solvent during the hydrogen bond formation process, as has been done in previous work.^31^Fig. 6 shows the decomposition of the complexation energy, Δ*E*_complex_, into a desolvation term, Δ*E*_desolv_, which accounts for desolvating the equilibrium structures in acetonitrile to the gas phase; a deformation term, Δ*E*_strain_, that describes the energy penalty originating from preparing the receptor to form a complex (the anion does not deform since it is a single atom); the interaction energy Δ*E*_int_, stemming from favorable interactions (in the gas-phase) between the deformed receptor and anion; and finally, a solvation term Δ*E*_solv_, a stabilizing term for solvating the complex.

We observe that [8+Cl^−^] is more stabilized than [3+Cl^−^] because the interaction between the receptor and anion is stronger for [8+Cl^−^], which is depicted by the Δ*E*_int_ term: −58.6 *vs.* −51.2 kcal mol^−1^ for [8+Cl^−^] and [3+Cl^−^], respectively (see Table 3). The energy differences of the strain energy are smaller (8.7 kcal mol^−1^ for 3 and 11.7 kcal mol^−1^ for 8) and also of the rearrangement (Δ*E*_desolv_ + Δ*E*_solv_) of the solvent (27.6 kcal mol^−1^ for 3 and 31.3 kcal mol^−1^ for 8).

**Table 3:** Decomposition of the total bonding energy Δ*E*_complex_ into its components (ASM, in kcal mol^−1^), and a further partitioning of the interaction energy Δ*E*_int_ of the hydrogen-bond interaction between receptor 3 or 8, and Cl^−^, computed at COSMO(ACN)-ZORA-BLYP-D3(BJ)/TZ2P

Complex	Δ*E*_complex_	Δ*E*_desolv_	Δ*E*_solv_	Δ*E*_strain_	Δ*E*_int_	Δ*V*_elstat_	Δ*E*_Pauli_	Δ*E*_oi_	Δ*E*_disp_
[3+Cl^−^]	−14.8	75.6	−48.0	8.7	−51.2	−43.8	26.2	−27.8	−5.7
[8+Cl^−^]	−15.7	73.3	−42.0	11.7	−58.6	−51.7	33.3	−33.7	−6.5
[8+I^−^]	−10.7	67.0	−44.8	9.0	−41.9	−39.6	29.0	−22.7	−8.6

Performing the EDA enables us to understand the difference in hydrogen bond strengths between the complexes. We see that the quasi-classical electrostatic interaction, Δ*V*_elstat_, is the predominant actor for explaining why [8+Cl^−^] forms a more stable complex as it becomes more favorable for receptor 8 to interact with chloride with a strength of −51.7 kcal mol^−1^ with respect to −43.8 kcal mol^−1^ belonging to [3+Cl^−^]. The steric repulsion, Δ*E*_Pauli_, shows the opposite trend because it becomes more destabilizing from 3 (26.2 kcal mol^−1^) to 8 (33.3 kcal mol^−1^), which stems from occupied orbitals overlapping with each other. Charge transfer between occupied–unoccupied orbitals on the receptor and anion, Δ*E*_oi_, and dispersion effects, Δ*E*_disp_, also make [8+Cl^−^] interact stronger, but are less significant than the electrostatic interactions.

The stronger electrostatic interactions between 8 and Cl^−^ compared to 3 can be understood from earlier studies, which revealed that the size of the chalcogen atom influences the hydrogen-bond capability in amides.^27,29,32^ The 
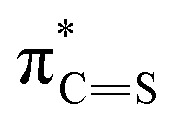
 is lower in energy than the 
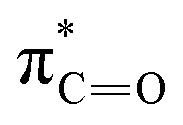
 and accepts more electronic density from the lone pair of the N–H group, which then leads to higher donor capability for thioamide than for amide as the charge of the hydrogen-bond donors, the NH groups, increases. For the current anion–receptor complexes, we observe the same phenomenon in which the NH groups become more positively charged: from +23 and +29 milli-electrons for 3 in [3+Cl^−^], to +64 and +66 milli-electrons for 8 in [8+Cl^−^] (Fig. 5). We thus conclude that Cl^−^ binds more strongly to 8 than 3 because the thioamide-based receptor interacts stronger with the anion, which agrees with experimental findings.

Lastly, we studied the origin of the selectivity observed experimentally, using Cl^−^ and I^−^ as the anions. Our computations reveal that both complexes adopt similar coordination modes in which the anion forms two hydrogen bonds with the two NH groups of the receptor (Fig. 5). The trend in computed (Gibbs free) energies agrees with experiments as [8+Cl^−^] is stabilized by −7.0 kcal mol^−1^ while [8+I^−^] by −0.6 kcal mol^−1^ upon forming the complex. Furthermore, we observe a significant elongation in the hydrogen bond distances, *r*_N(H)⋯X_ when Cl^−^ is substituted by I^−^.

Since the complexes share the same receptor, the larger size of the anion (I^−^*vs.* Cl^−^) causes the hydrogen bond to weaken significantly, as can be seen in Table 3. The interaction energy is considerably more stabilizing for [8+Cl^−^] (−58.6 kcal mol^−1^) than for [8+I^−^] (−41.9 kcal mol^−1^) which compensates for the increase in strain energy (11.7 kcal mol^−1^*vs.* 9.0 kcal mol^−1^) and rearrangement (Δ*E*_desolv_ + Δ*E*_solv_) of the solvent (31.3 kcal mol^−1^*vs.* 22.2 kcal mol^−1^). The reason why the larger anion interacts more weakly is that the hydrogen bond length is longer, which coincides with less favorable electrostatic interactions between the two positively charged NH groups of the receptor and the negatively charged anion. Furthermore, the orbital overlap between the occupied np orbital of the anion and unoccupied orbitals 
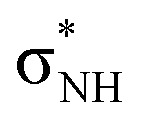
 on the receptor becomes smaller and contributes less to the hydrogen bonds (see Table S5 for the orbital interaction diagram). Hence, 8 favors Cl^−^ over I^−^ because the hydrogen bond lengths are shorter and stronger, resulting in more favorable electrostatic and orbital interactions.

## Conclusions

In summary, we designed acyclic receptors containing (thio)amide groups in an anti-parallel orientation; however, in the presence of anions, they reorient so that the two N–H groups align in parallel, creating a cavity for binding. The higher affinity of thioamides for anions is primarily driven by electrostatic interactions, owing to the larger size of sulfur compared to oxygen. The size of the anion is also critical for forming an optimal cavity, as larger anions do not fit as effectively. Overall, this study provides new insights that can inform the design of acyclic anion receptors.

## Author contributions

Conceptualization: N. Akhtar and V. García-López. Data curation, formal analysis, investigation, methodology, validation: N. Akhtar, S. Lekanne Deprez, S. Jayawardana, M. Davis. Funding acquisition, project administration, supervision: C. Fonseca Guerra and V. García-López. Writing – original draft: N. Akhtar, S. Lekanne Deprez, C. Fonseca Guerra, and V. García-López.

## Conflicts of interest

The authors do not have conflicts of interest associated with this publication.

## Supplementary Material

RA-015-D5RA08433D-s001

RA-015-D5RA08433D-s002

## Data Availability

The data originated in this study has been included as part of the supplementary information (SI). This includes all experimental details, spectra, procedures, and crystallographic data. Supplementary information is available. See DOI: https://doi.org/10.1039/d5ra08433d.

## References

[cit1] Macreadie L. K., Gilchrist A. M., McNaughton D. A., Ryder W. G., Fares M., Gale P. A. (2022). Chem.

[cit2] Wang Q.-Q. (2024). Acc. Chem. Res..

[cit3] Tay H. M., Beer P. (2021). Org. Biomol. Chem..

[cit4] Busschaert N., Caltagirone C., Van Rossom W., Gale P. A. (2015). Chem. Rev..

[cit5] Akhtar N., Conthagamage U. N. K., Bucher S. P., Abdulsalam Z. A., Davis M. L., Beavers W. N., García-López V. (2024). Mater. Adv..

[cit6] Gale P. A., Pérez-Tomás R., Quesada R. (2013). Acc. Chem. Res..

[cit7] Gale P. A., Davis J. T., Quesada R. (2017). Chem. Soc. Rev..

[cit8] Choi K., Hamilton A. D. (2003). Coord. Chem. Rev..

[cit9] Dong J., Davis A. P. (2021). Angew. Chem..

[cit10] McNaughton D. A., Ryder W. G., Gilchrist A. M., Wang P., Fares M., Wu X., Gale P. A. (2023). Chem.

[cit11] Vargas-Zúñiga G. I., Sessler J. L. (2017). Coord. Chem. Rev..

[cit12] Pfeffer F. M., Lim K. F., Sedgwick K. J. (2007). Org. Biomol. Chem..

[cit13] Amendola V., Bergamaschi G., Boiocchi M., Fabbrizzi L., Milani M. (2010). Chem.–Eur J..

[cit14] Kundu S., Egboluche T. K., Hossain Md. A. (2023). Acc. Chem. Res..

[cit15] Kang S. O., Hossain Md. A., Bowman-James K. (2006). Coord. Chem. Rev..

[cit16] Kang S. O., Llinares J. M., Day V. W., Bowman-James K. (2010). Chem. Soc. Rev..

[cit17] Mohammed F. A., Xiao T., Wang L., Elmes R. B. P. (2024). Chem. Commun..

[cit18] Chen L., Berry S. N., Wu X., Howe E. N. W., Gale P. A. (2020). Chem.

[cit19] Gale P. A. (2006). Acc. Chem. Res..

[cit20] Kavallieratos K., De Gala S. R., Austin D. J., Crabtree R. H. (1997). J. Am. Chem. Soc..

[cit21] Kavallieratos K., Bertao C. M., Crabtree R. H. (1999). J. Org. Chem..

[cit22] Makuc D., Lenarčič M., Bates G. W., Gale P. A., Plavec J. (2009). Org. Biomol. Chem..

[cit23] Gavette J. V., Evoniuk C. J., Zakharov L. N., Carnes M. E., Haley M. M., Johnson D. W. (2014). Chem. Sci..

[cit24] Turner D. R., Paterson M. J., Steed J. W. (2006). J. Org. Chem..

[cit25] Hossain Md. A., Kang S. O., Llinares J. M., Powell D., Bowman-James K. (2003). Inorg. Chem..

[cit26] Nieuwland C., Van Dam A. N., Bickelhaupt F. M., Fonseca Guerra C. (2025). Phys. Chem. Chem. Phys..

[cit27] Nieuwland C., Fonseca Guerra C. (2024). Chem.–Eur J..

[cit28] Nieuwland C., Fonseca Guerra C. (2022). Chem.–Eur J..

[cit29] Nieuwland C., Lekanne Deprez S., De Vries C., Fonseca Guerra C. (2023). Chem.–Eur J..

[cit30] Riwar L., Trapp N., Kuhn B., Diederich F. (2017). Angew. Chem., Int. Ed..

[cit31] Nieuwland C., Zaccaria F., Fonseca Guerra C. (2020). Phys. Chem. Chem. Phys..

[cit32] Nieuwland C., Verdijk R., Fonseca Guerra C., Bickelhaupt F. M. (2024). Chem.–Eur J..

