# Supplementary material for: From amides to thioamides: understanding enhanced anion binding in acyclic receptors
Source: RSC Adv. 2025 Nov 28;15(55):46908–13. doi: 10.1039/d5ra08433d (PMC12661136; doi:10.1039/d5ra08433d)
Supplement: RA-015-D5RA08433D-s001 [file RA-015-D5RA08433D-s001.pdf]

Supplementary Information (SI) for RSC Advances.
This journal is © The Royal Society of Chemistry 2025

From Amides to Thioamides: Understanding Enhanced Anion Binding in Acyclic Receptors.

Nasim Akhtar,[a] Siebe Lekanne Deprez,[b] Senuri G. Jayawardana,[a] Macallister Davis,[a] Prof. Dr. Célia Fonseca Guerra,*[b] and Dr. Víctor García-López*[a]

a. Department of Chemistry, Louisiana State University, Baton Rouge, LA 70803, United States of America.

b. Department of Chemistry and Pharmaceutical Sciences, Amsterdam Institute for Molecular and Life Sciences (AIMMS), Vrije Universiteit Amsterdam, Amsterdam 1081 HZ, The Netherlands.

* Corresponding authors: c.fonsecaguerra@vu.nl, vglopez@lsu.edu

Table of Contents

S1 Materials and General Experimental Methods 1
S2 Synthetic Procedures and Characterization 2
S3 Structural determination using 2D NMR. 12
S4 Binding Studies in Solution 14
S5 Other fitting models for receptors 3 and 8. 24
S6 Anion Selectivity 27
S7 Mass Spectrometric Study 30
S8 Crystal structure details 33
S9 Computational Details 53
S10 Figures and Tables from Computational Study 56
S11 Cartesian Coordinates 57
S12 NMR spectra of synthesized compounds 78
S13 Mass Spectra 90
S14 References 95

S1 Materials and General Experimental Methods

All the glassware for synthesis was dried overnight in an oven before use. All the reactions were carried out under an inert atmosphere unless otherwise stated in the individual protocols. All the required chemicals and solvents were purchased from different commercial sources and were used directly without purification. Solvents such as tetrahydrofuran, dimethylformamide, acetonitrile, dichloromethane, and toluene were dried in a solvent purification system (Pure Process Technology) under an argon atmosphere. ACS-grade solvents were used for liquid extractions as well as for column chromatography. Flash column chromatography (FC) was performed using a Buchi Pure FlashPrep C-850 Chromatography System using silica as the stationary phase (60 Å, 230-400 mesh, silicycle) at 21 °C. The reaction progress was monitored by thin-layer chromatography (TLC) using aluminum sheets coated with silica gel 60 F254 (Supelco, Sigma Aldrich), and visualization was done with UV light (254/365 nm). ^{1}H nuclear magnetic resonance (NMR) spectra were recorded using Bruker AV III 400 and Bruker AV III 500 spectrometers. All the ^{13}C NMR spectra were recorded on a Bruker AV III 500 spectrometer. Chemical shifts (δ) values are reported in ppm using the residual non-deuterated solvent signals as an internal reference (chloroform-d: δ_H = 7.26 ppm, δ_C = 77.16 ppm; acetone-d_6: δ_H = 2.05 ppm, δ_C = 29.84 ppm; acetonitirile-d_3: δ_H = 1.94 ppm, δ_C = 1.32 ppm; methanol-d_4: δ_H = 3.31 ppm, δ_C = 49.00 ppm, dimethyl sulfoxide-d$_6$: δ_H = 2.50 ppm, δ_C = 39.52 ppm). Coupling constant (*J*) values are recorded in hertz (Hz), and resonance multiplicity of peaks are described as s (singlet), d (doublet), t (triplet), q (quartet), m (multiple), and brs (broad singlet). High-resolution mass spectrometry (HR-MS) was performed by the Mass Spectrometry Facility at Louisiana State University using an Agilent 6230 ESI TOF and a Bruker rapifleX MALDI TOF/TOF.

S2 Synthetic Procedures and Characterization

S2.1. 4-(benzhydrylamino)-4-oxobutanoic acid (12)

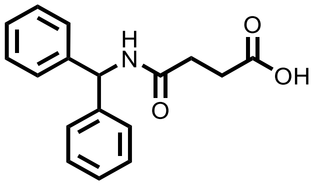

Adapted from reported procedure,[1] a stirred solution of diphenylmethanamine (100 mg, 0.54 mmol), 4-(dimethylamino)pyridine (13 mg, 0.11 mmol), and triethylamine (83 µL, 0.60 mmol) in acetonitrile (10 mL) was prepared in a 100 mL round bottom flask, to which succinic anhydride (54 mg, 0.54 mmol) was added. The reaction mixture was stirred for 12 h at room temperature under N_2 atmosphere. The progress of the reaction was monitored by thin-layer chromatography. Upon completion, the reaction mixture was concentrated under reduced pressure and diluted with 1 (N) aqueous hydrochloric acid to remove the 4-(dimethylamino)pyridine. Subsequently, the mixture was extracted with ethyl acetate (3 × 10 mL). The organic layer was washed with brine and dried over anhydrous magnesium sulfate to remove residual water. Removal of the solvent under reduced pressure afforded a solid, which was thereafter washed with diethyl ether to obtain the product as a white solid (108 mg, 70%). ^{1}H NMR (400 MHz, dimethyl sulfoxide-*d*$_6$) δ 12.06 (brs, 1H), 8.78 (d, *J* = 8.7 Hz, 1H), 7.34 – 7.21 (m, 10H), 6.10 (d, *J* = 8.7 Hz, 1H), 2.46 – 2.45 (m, 4H). ^{13}C NMR (125 MHz, dimethyl sulfoxide-*d*$_6$) δ 173.89, 170.38, 142.64, 128.34, 127.32, 126.89, 55.87, 29.99, 29.12. HRMS (ESI) (m/z): Calcd for $C_{17}H_{17}NO_3$ [M+H]$^+$ 284.1281, found: 284.1293; $C_{17}H_{17}NO_3$ [M+Na]$^+$ 306.1101, found: 306.1119.

S2.2. 4-((3,5-bis(trifluromethyl)benzyl)amino)-4-oxobutanoic acid (14)

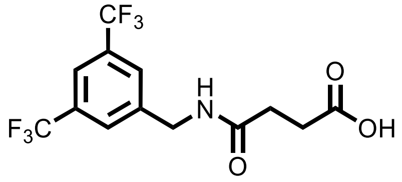

A stirred solution of 3,5-bis(trifluoromethyl)benzylamine (100 mg, 0.41 mmol), *4-*(Dimethylamino)pyridine (10 mg, 0.082 mmol), and triethylamine (62 µL, 0.45 mmol)

in acetonitrile was prepared in a 100 mL round-bottom flask to which succinic anhydride (54 mg, 0.54 mmol) was added. The reaction mixture was stirred for 12 h at room temperature under N_2 atmosphere. The progress of the reaction was monitored by thin-layer chromatography. Upon completion, the reaction mixture was concentrated under reduced pressure and diluted with 1 (N) aqueous hydrochloric acid to remove the *4*-(dimethylamino)pyridine. Subsequently, the mixture was extracted with ethyl acetate (3 × 30 mL). The organic layer was washed with brine and dried over anhydrous magnesium sulfate to remove residual water. Removal of the solvent under reduced pressure afforded a white precipitate, which was thereafter washed with diethyl ether to obtain the product as a while solid (91 mg, 65%). ^{1}H NMR (400 MHz, methanol-*d₄*) δ 7.90 (s, 2H), 7.84 (s, 1H), 4.52 (s, 2H), 2.65 – 2.62 (m, 2H), 2.56 – 2.53 (m, 2H). ^{13}C NMR (125 MHz, methanol-d_4) δ 176.05, 174.95, 143.80, 132.79 (q, *J* = 33.2 Hz), 129.04, 129.00, 125.94, 123.77, 121.87, 121.83, 121.80, 121.77,121.61, 43.25, 31.41, 30.00. HRMS (ESI) (m/z): Calcd for $C_{13}H_{11}F_6NO_3$ $[M+H]^+$ 344.0716, found: 344.0729.

S2.3. Receptor 1

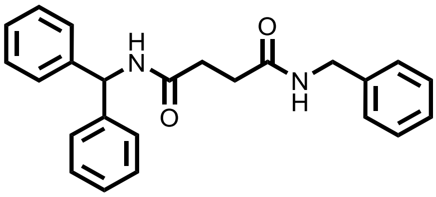

4-(benzhydrylamino)-4-oxobutanoic acid **12** (100 mg, 0.35 mmol) and *N,N,N',N'*-tetramethyl-*O*-(1*H*-benzotriazol-1-yl)uronium hexafluorophosphate *(*147 mg, 0.38 mmol*)* were dissolved in dry dimethylformamide (3.0 mL) in a 100 mL two-neck round-bottom flask under a N_2 atmosphere. *N*,*N*-diisopropylethylamine (184 μL, 1.05 mmol) was added, and the reaction mixture was stirred at room temperature for 1 h. After 1 h, a solution of aniline (38 μL, 0.42 mmol) in dry dimethylformamide (2.0 mL) was added slowly, and the reaction was stirred for 12 h. The progress of the reaction was monitored by thin-layer chromatography. Upon completion, the reaction mixture was concentrated under reduced pressure and extracted with ethyl acetate (3 × 30 mL). To remove residual high-boiling dimethylformamide, chilled water (3 × 20 mL) was

used during the extraction procedure. Subsequently, the organic layer was washed with 10% citric acid followed by the 10% sodium bicarbonate and brine to remove other byproducts. Finally, the organic layer was concentrated under reduced pressure and the residue was washed repeatedly with diethyl ether and hexane to remove the tetramethylurea byproduct, affording the product as a white solid (309 mg, 83%).^{1}H NMR (400 MHz, dimethyl sulfoxide-d_6) δ 8.79 (d, J = 8.6 Hz, 1H), 8.34 (t, J = 5.6 Hz, 1H), 7.33 – 7.22 (m, 15H), 6.11 (d, J = 8.6 Hz, 1H), 4.25 (d, J = 5.9 Hz, 2H), 2.48 (s, 2H), 2.42 – 2.39 (m, 2H). ^{13}C NMR (125 MHz, dimethyl sulfoxide-d_6) δ 171.37, 170.71, 142.68, 139.61, 128.35, 128.24, 127.28, 127.16, 126.88, 126.68, 55.85, 42.03, 30.74. HRMS (ESI) (m/z): Calcd for $C_{24}H_{24}N_2O_2$ [M+Na]$^+$ 395.1730, found: 395.1726; $C_{48}H_{48}N_4O_4$ [2M+Na]$^+$ 767.3568, found 767.3565.

S2.4. Receptor 2

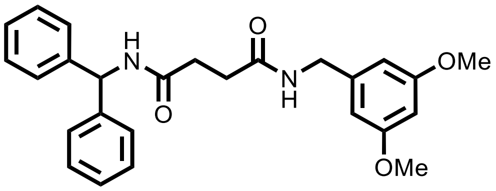

4-(benzhydrylamino)-4-oxobutanoic acid **12** (100 mg, 0.35 mmol) and *N,N,N',N'*-Tetramethyl-*O*-(1*H*-benzotriazol-1-yl)uronium hexafluorophosphate (147 mg, 0.38 mmol) were dissolved in dry dimethylformamide (3.0 mL) in a 100 mL two-neck round-bottom flask under a N_2 atmosphere. *N,N*-diisopropylethylamine (184 µL, 1.05 mmol) was added, and the reaction mixture was stirred at room temperature for 1 h. Then, a solution of (3,5-dimethoxyphenyl)methanamine (71 mg, 0.42 mmol) in dry dimethylformamide (2.0 mL) was added, and the reaction was stirred overnight (12 h). The progress of the reaction was monitored by thin-layer chromatography. Upon completion, the reaction mixture was concentrated under reduced pressure and extracted with ethyl acetate (3 × 30 mL). To remove residual high-boiling dimethylformamide, cold water was used during the extraction procedure. Subsequently, the organic layer was washed with 10% citric acid, 10% sodium bicarbonate, and brine to remove other byproducts. Finally, the organic layer was concentrated under reduced pressure. The residue was washed repeatedly with diethyl ether and hexane to remove the tetramethylurea byproduct, affording the

product as a white solid (389 mg, 90%). ^{1}H NMR (500 MHz, methanol-*d4*) δ 7.32 – 7.29 (m, 4H), 7.25 – 7.22 (m, 6H), 6.44 (d, *J* = 1.7 Hz, 2H), 6.34 (t, *J* = 2.4 Hz, 1H), 6.16 (s, 1H), 4.28 (s, 2H), 3.72 (s, 6H), 2.63 (t, *J* = 6.9 Hz, 2H), 2.56 (t, *J* = 6.9 Hz, 2H). ^{13}C NMR (125 MHz, methanol-*d4*) δ 174.57, 173.70, 162.47, 143.18, 142.30, 129.50, 128.64, 128.27, 106.33, 100.10, 58.26, 55.72, 44.20, 32.16, 32.11. HRMS (ESI) (m/z): Calcd for $C_{26}H_{28}N_2O_4$ $[M+Na]^+$ 455.1941, found: 455.1937; $C_{52}H_{56}N_4O_8$ $[2M+Na]^+$ 887.3990, found: 887.3987.

S2.5. Receptor 3.

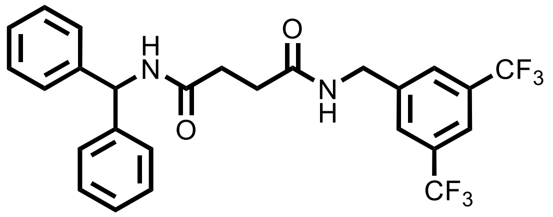

4-(benzhydrylamino)-4-oxobutanoic acid **12** (100 mg, 0.35 mmol) and *N,N,N',N'*-Tetramethyl-*O*-(1*H*-benzotriazol-1-yl)uronium hexafluorophosphate *(*147 mg, 0.38 mmol*)* were dissolved in dry dimethylformamide (3.0 mL) in a 100 mL two-neck round-bottom flask under a N_2 atmosphere. *N*,*N*-diisopropylethylamine (184 μL, 1.05 mmol) was added and reaction mixture was stirred at room temperature for 1 h. After 1 h, a solution of 3,5-bis(trifluoromethyl)benzylamine (103 mg, 0.42 mmol) in dry dimethylformamide (2 mL) was added and the reaction was stirred for 12 h. The progress of the reaction was monitored by thin-layer chromatography. Upon completion, the reaction mixture was concentrated under reduced pressure and extracted with ethyl acetate (3 $\times$ 30 mL). To remove residual high-boiling dimethylformamide, chilled water was used during the extraction procedure. Subsequently, the organic layer was washed with 10% citric acid, 10% sodium bicarbonate, and brine to remove other byproducts. Finally, the organic layer was concentrated under reduced pressure, and the residue was washed repeatedly with diethyl ether and hexane to remove the tetramethylurea byproduct, affording the product as a white solid (431 mg, 85%). ^{1}H NMR (500 MHz, methanol-*d4*) δ 7.89 (s, 2H), 7.83 (s, 1H), 7.31 – 7.28 (m, 4H), 7.24 – 7.22 (m, 4H), 6.17 (s, 1H), 4.50 (s, 2H), 2.64 (t, J = 6.5 Hz, 2H), 2.58 (t, *J* = 6.5 Hz, 2H); ^{13}C NMR (125 MHz, methanol-*d4*) δ 175.03, 173.58, 143.76, 143.20, 129.48, 129.12, 128.63, 128.25, 125.93, 123.77,

121.85, 58.18, 43.29, 31.96. HRMS (ESI) (m/z): Calcd for $C_{26}H_{22}F_6N_2O_2$ $[M+Na]^+$ 531.1478, found: 531.1481; $C_{52}H_{44}F_{12}N_4O_4$ $[2M+Na]^+$ 1039.3063, found: 1039.3078.

S2.6. Receptor 4.

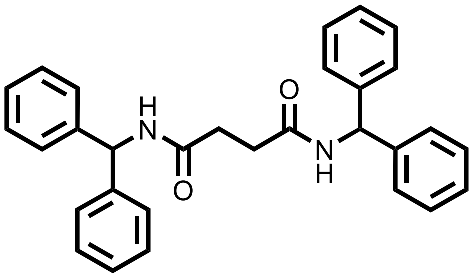

4-(benzhydrylamino)-4-oxobutanoic acid **12** (100 mg, 0.35 mmol) and *N,N,N',N'*-Tetramethyl-*O*-(1*H*-benzotriazol-1-yl)uronium hexafluorophosphate (147 mg, 0.38 mmol) were dissolved in dry dimethylformamide (3.0 mL) in a 100 mL two-neck round-bottom flask under a N_2 atmosphere. DIPEA (184 µL, 1.05 mmol) was added and the reaction was stirred at room temperature for 1 h. After 1h, a solution of diphenylmethanamine (73 µL, 0.42 mmol) in dry dimethylformamide (2.0 mL) was added and the reaction was stirred overnight (12 h). The progress of the reaction was monitored by thin layer chromatography. Upon completion, the reaction mixture was concentrated under reduced pressure and extracted with ethyl acetate (3 × 30 mL). To remove residual high-boiling dimethylformamide, cold water was used during the extraction procedure. Subsequently, the organic layer was washed with 10% citric acid, 10% sodium bicarbonate, and brine to remove other byproducts. Finally, the organic layer was concentrated under reduced pressure and the residue was washed repeatedly with diethyl ether and hexane to remove the tetramethylurea byproduct, affording the product as a white solid (358 mg, 80%). ^1H NMR (500 MHz, methanol-*d*$_4$) δ 7.31 – 7.28 (m, 9H), 7.25 – 7.22 (m, 13H), 6.16 (s, 2H), 2.63 (s, 4H). ^{13}C NMR (125 MHz, methanol-*d*$_4$) δ 173.78, 143.16, 129.51, 128.64, 128.27, 58.26, 32.12, 30.75. HRMS (ESI) (m/z): Calcd for $C_{30}H_{28}N_2O_2$ $[M+Na]^+$ 471.2043, found:471.2028; $C_{60}H_{56}N_4O_4$ $[2M+Na]^+$ 919.4194, found: 919.4153.

S2.7. Receptor 5.

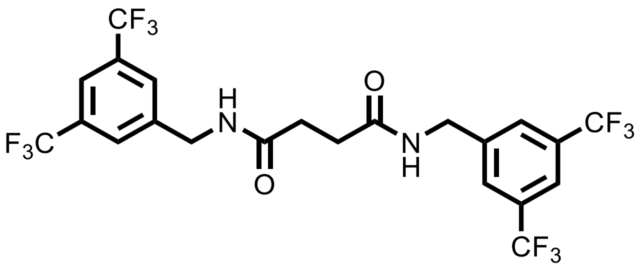

4-((3,5-bis(trifluromethyl)benzyl)amino)-4-oxobutanoic acid **14** (100 mg, 0.29 mmol) and *N,N,N',N'*-Tetramethyl-*O*-(1*H*-benzotriazol-1-yl)uronium hexafluorophosphate *(*147 mg, 0.32 mmol) was dissolved in dry dimethylformamide (3.0 mL) in a 100 mL two-neck round-bottom flask under a N_2 atmosphere. *N*,*N*-diisopropylethylamine (151 µL, 0.87 mmol) was added and the reaction mixture was stirred at room temperature for 1 h. After 1h, a solution of 3,5-Bis(trifluoromethyl)benzylamine (73 µL, 0.42 mmol) in dry dimethylformamide (2.0 mL) was added and the reaction was stirred overnight (12 h). The progress of the reaction was monitored by thin layer chromatography. Upon completion, the reaction mixture was concentrated under reduced pressure and extracted with ethyl acetate (3 × 30 mL). To remove residual high boiling dimethylformamide, chilled water was used during the extraction procedure. Subsequently, the organic layer was washed with 10% citric acid, 10% sodium bicarbonate, and brine to remove the other byproducts. Finally, the organic layer was concentrated under reduced pressure, and the residue was washed repeatedly with diethyl ether and hexane to remove tetramethyl byproduct, affording the product as a white solid (358 mg, 80%). ^{1}H NMR (500 MHz, methanol-*d₄*) δ 7.89 (s, 4H), 7.82 (s, 2H), 4.51 (s, 4H), 2.60 (s, 4H). ^{13}C NMR (125 MHz, methanol-d_4) δ 174.86, 143.86, 132.76 (q, *J* = 33.2 Hz), 129.03, 125.93, 123.77, 121.78, 43.19, 31.61. HRMS (ESI) (m/z): Calcd for $C_{22}H_{16}F_{12}N_2O_2$ $[M+H]^+$ 569.1093, found: 569.1110.

S2.8. Receptor 6.

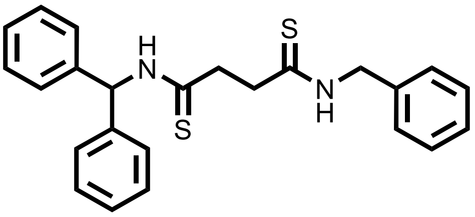

N^1-benzhydryl-N^4-benzylsuccinamide **1** (100 mg, 0.27 mmol) and Lawesson's reagent (543 mg, 1.34 mmol) were dissolved in dry tetrahydrofuran (10.0 mL) in a 100 mL two-neck round-bottom flask under a N_2 atmosphere. The reaction mixture was then stirred at 60 °C for 12 h. The progress of the reaction was monitored by thin-layer chromatography. Upon completion, the solvent was evaporated under reduced pressure, and aqueous sodium bicarbonate (20 mL) was added to remove byproducts and extracted with ethyl acetate (3 × 30 mL). The organic layer was washed with brine, dried over anhydrous magnesium sulfate to remove residual water, and concentrated under reduced pressure. The crude was purified by flash chromatography (silica, ethyl acetate/hexane solvent system) to obtain compound **6** as a white solid (202 mg, 50%). ^{1}H NMR (400 MHz, dimethyl sulfoxide-*d*$_6$) δ 10.87 (d, *J* = 8.4 Hz, 1H), 10.47 (t, *J* = 5.4 Hz, 1H), 7.37 – 7.25 (m, 15H), 6.91 (d, *J* = 8.5 Hz, 1H), 4.78 (d, *J* = 5.6 Hz, 2H), 3.13 – 3.09 (m, 2H), 3.05 – 3.01 (m, 2H). ^{13}C NMR (125 MHz, dimethyl sulfoxide-*d*$_6$) δ 202.63, 202.20, 140.56, 137.13, 128.46, 128.32, 127.72, 127.70, 127.33, 127.13, 61.65, 48.31, 43.38, 43.29. HRMS (ESI) (m/z): Calcd for $C_{24}H_{24}N_2S_2$ $[M+H]^+$ 405.1454, found:471.1460; $C_{24}H_{24}N_2S_2$ $[M+Na]^+$ 427.1273, found: 427.1276.

S2.9. Receptor 7.

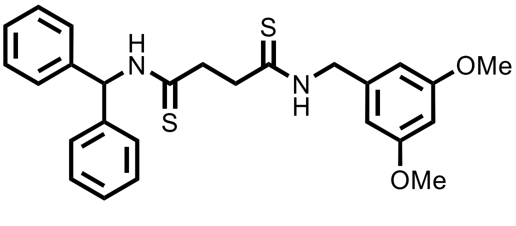

N^1-benzhydryl-N^4-(3,5-dimethoxybenzyl)succinamide **2** (100 mg, 0.231 mmol) and Lawesson's reagent (467 mg, 1.15 mmol) were dissolved in dry tetrahydrofuran (10.0 mL) in a 100 mL two-neck round-bottom flask under a N_2 atmosphere. The reaction

mixture was then stirred at 60 °C overnight (12 h). The progress of the reaction was monitored by thin-layer chromatography. Upon completion, the solvent was evaporated under reduced pressure, and aqueous sodium bicarbonate (20 mL) was added in it to remove byproducts and extracted with ethyl acetate (3 × 30 mL). The organic layer was washed with brine, dried over anhydrous magnesium sulfate to remove residual water, and concentrated under reduced pressure. The crude was purified by flash chromatography (silica, ethyl acetate/hexane solvent system) to obtain compound **7** as a white solid (209 mg, 45%). ^{1}H NMR (400 MHz, acetonitrile-d_3) δ 9.13 (s, 1H), 8.69 (s, 1H), 7.36 – 7.33 (m 4H) 7.30 – 7.29 (m, 2H), 7.26 – 7.25 (m 4H), 6.81 (d, *J* = 8.1 Hz, 2H), 6.45 (t, *J* = 2.1 Hz, 2H), 6.38 (t, *J* = 2.1, 1H),4.72 (d, *J* = 5.6 Hz, 2H), 3.73 (s, 6H), 3.18 (t, *J* = 6.6 Hz, 2H), 3.10 (t, *J* = 6.6 Hz, 2H). ^{13}C NMR (125 MHz, chloroform-*d*) δ 202.76, 202.43, 161.30, 139.90, 137.88, 128.97, 128.04, 106.20, 100.35, 63.13, 55.57, 50.78, 45.36, 45.19, 29.85. HRMS (ESI) (m/z): Calcd for $C_{26}H_{28}N_2O_2S_2$ $[M+H]^+$ 465.1592, found:465.1669; $C_{26}H_{28}N_2O_2S_2Na^+$ $[M+Na]^+$ 487.1484, found: 487.1480; $C_{26}H_{28}N_2O_2S_2K^+$ $[M+K]^+$ 503.1224, found: 503.1225.

S2.10. Receptor 8.

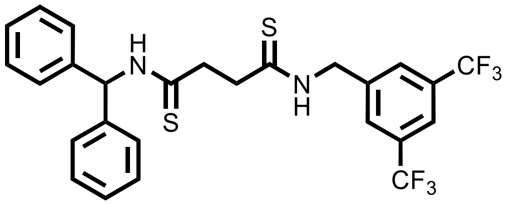

N[1]-benzhydryl-*N*[4]-(3,5-bis(trifluoromethyl)benzyl)succinamide **3** (100 mg, 0.2 mmol) and Lawesson's reagent (397 mg, 0.98 mmol) were dissolved in dry tetrahydrofuran (10.0 mL) in a 100 mL two-neck round-bottom flask under N_2 atmosphere. The reaction mixture was stirred at 60 °C overnight (12 h). The progress of the reaction was monitored by thin-layer chromatography. Upon completion, the solvent was evaporated under reduced pressure and aqueous sodium bicarbonate (20 mL) was added to the concentrated residue to remove byproducts and extracted with ethyl acetate (3 × 30 mL). The organic layer was washed with brine, dried over anhydrous magnesium sulfate to remove residual water, and concentrated under reduced pressure. The crude was purified by flash chromatography (silica, ethyl acetate/hexane solvent system) to obtain compound **8** as a white solid (254 mg,

47%).^{1}H NMR (500 MHz, chloroform-*d*) δ 8.12 (d, J = 8.2 Hz, 1H), 8.02 (t, J = 5.4 Hz, 1H), 7.79 (s, 1H), 7.65 (s, 2H), 7.35 – 7.32 (m, 4H), 7.30 – 7.27 (m, 2H), 7.23 – 7.21 (m, 4H), 6.83 (d, J = 8.1 Hz, 1H), 4.76 (d, J = 5.6 Hz, 2H), 3.22 (s, 4H). ^{13}C NMR (125 MHz, chloroform-*d*) δ 204.25, 139.48, 138.33, 131.95 (q, J = 33.4 Hz), 128.73, 128.17, 128.14, 127.86,127.62, 124.05, 121.88, 121.85, 121.82, 121.79, 62.77, 48.53, 44.82, 44.70. HRMS (ESI) (m/z): Calcd for $C_{26}H_{22}F_6N_2S_2$ $[M+H]^+$ 541.1201, found:541.1214.

S2.11. Receptor 9.

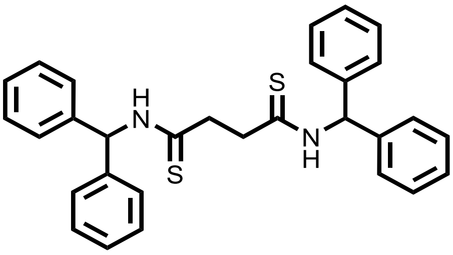

*N^1^,N^4^*-dibenzhydrylsuccinamide **4** (100 mg, 0.22 mmol), and Lawesson's reagent (450 mg, 1.11 mmol) were dissolved in dry tetrahydrofuran (10.0 mL) in a 100 mL two-neck round-bottom flask under a N_2 atmosphere. Next, the reaction mixture was stirred at 60 °C for overnight (12 h). The progress of the reaction was monitored by thin layer chromatography. Upon completion, the solvent was evaporated under reduced pressure and aqueous sodium bicarbonate (20 mL) was added to the concentrated residue to remove byproducts and extracted with ethyl acetate (3 × 30 mL). The organic (ethyl acetate) layer was washed with brine, dried over anhydrous magnesium sulfate to remove residual water, and concentrated under reduced pressure. The crude was purified by flash chromatography (silica, using ethyl acetate/hexane solvent system) to obtain compound **9** as a white solid (192 mg, 40%).^{1}H NMR (500 MHz, acetonitrile-d_3) δ 9.10 (brs, 2H), 7.35 – 7.32 (m, 8H), 7.30 – 7.28 (m, 4H), 7.27 – 7.24 (m, 8H) 6.81 (d, J = 7.9 Hz, 1H), 3.18 (s, 4H).^{13}C NMR (125 MHz, acetonitrile-d_3) δ 204.30, 141.56, 129.60, 128.73, 128.55, 63.64, 44.67. HRMS (ESI) (m/z): Calcd for $C_{30}H_{28}N_2S_2$ $[M+H]^+$ 481.1767, found:481.1776.

S2.12. Receptor 10.

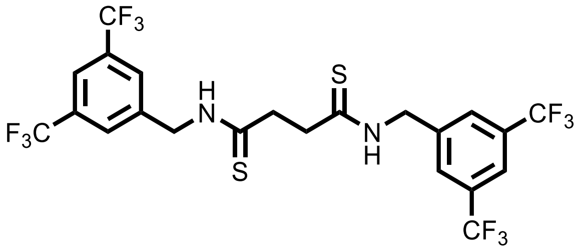

N[1],*N*[4]-bis(3,5-bis(trifluoromethyl)benzyl)succinimide **5** (100 mg, 0.18 mmol) and Lawesson's reagent (397 mg, 0.9 mmol) were dissolved in dry tetrahydrofuran (10.0 mL) in a 100 mL two-neck round-bottom flask under a N_2 atmosphere. The reaction mixture was stirred at 60 °C for 12 h. The progress of the reaction was monitored by thin layer chromatography. Upon completion, the solvent was evaporated under reduced pressure, and aqueous sodium bicarbonate (20 mL) was added to the concentrated residue to remove byproducts and extracted with ethyl acetate (3 × 30 mL). The organic layer was washed with brine, dried over anhydrous magnesium sulfate to remove the residual water, and concentrated under reduced pressure. The crude was purified by flash chromatography (silica, ethyl acetate/hexane solvent system) to obtain compound **10** as a white solid (58 mg, 55%). ^{1}H NMR (500 MHz, acetonitrile-d_3) δ 8.87 (s, 2H), 7.88 (s, 6H), 4.94 (d, J = 5.8 Hz, 4H), 3.14 (s, 4H). ^{13}C NMR (125 MHz, chloroform-d) δ 204.87, 138.74, 132.40, 132.13, 128.22, 124.33, 122.06, 48.60, 44.07. HRMS (ESI) (m/z): Calcd for $C_{22}H_{16}F_{12}N_2S_2$ $[M+H]^+$ 601.0636, found: 601.0648.

S3 Structural determination using 2D NMR.

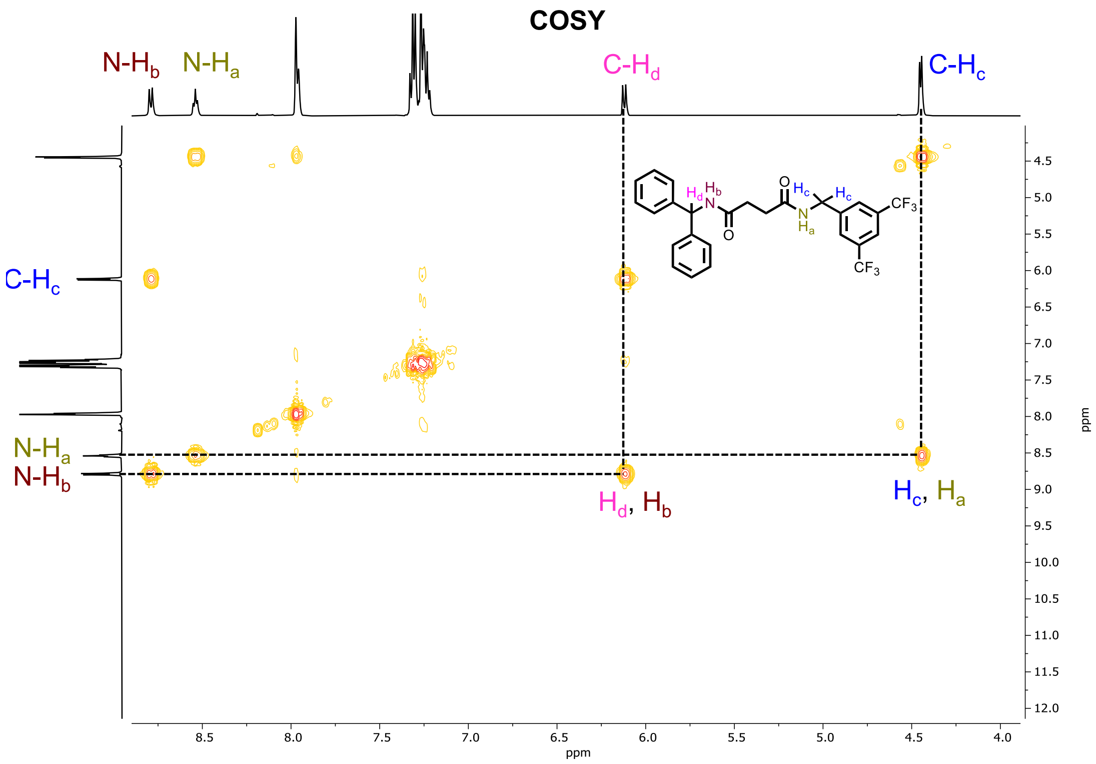

Figure S1. Structural determination of compound **3** using COSY NMR (500 MHz).

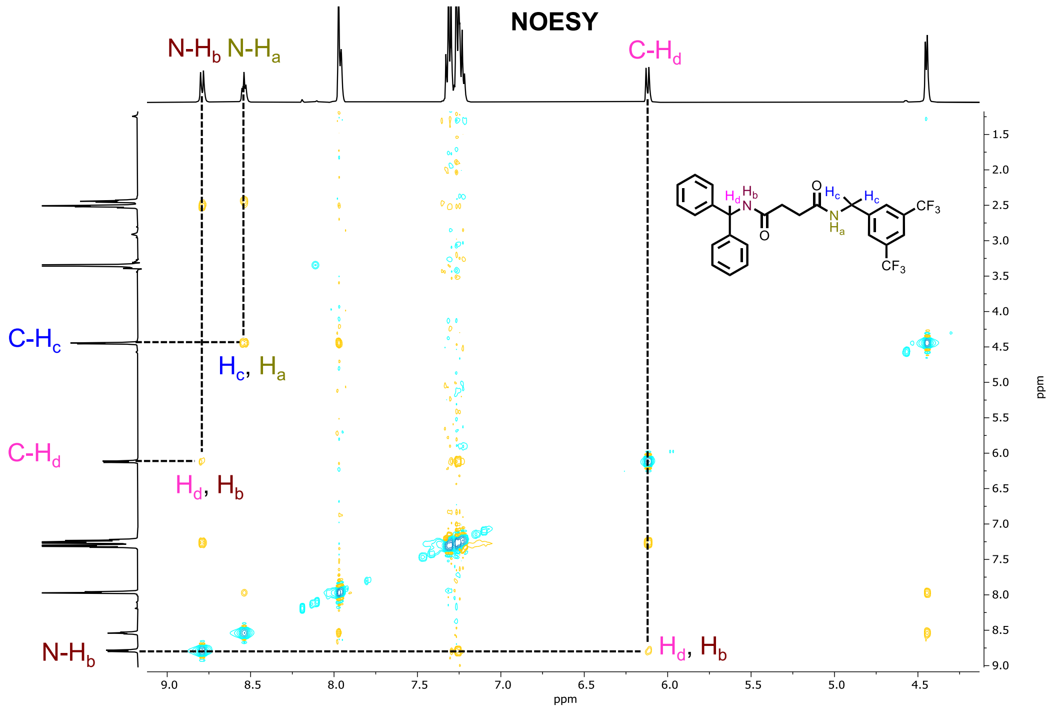

Figure S2. Structural determination of compound **3** using NOESY NMR (500 MHz).

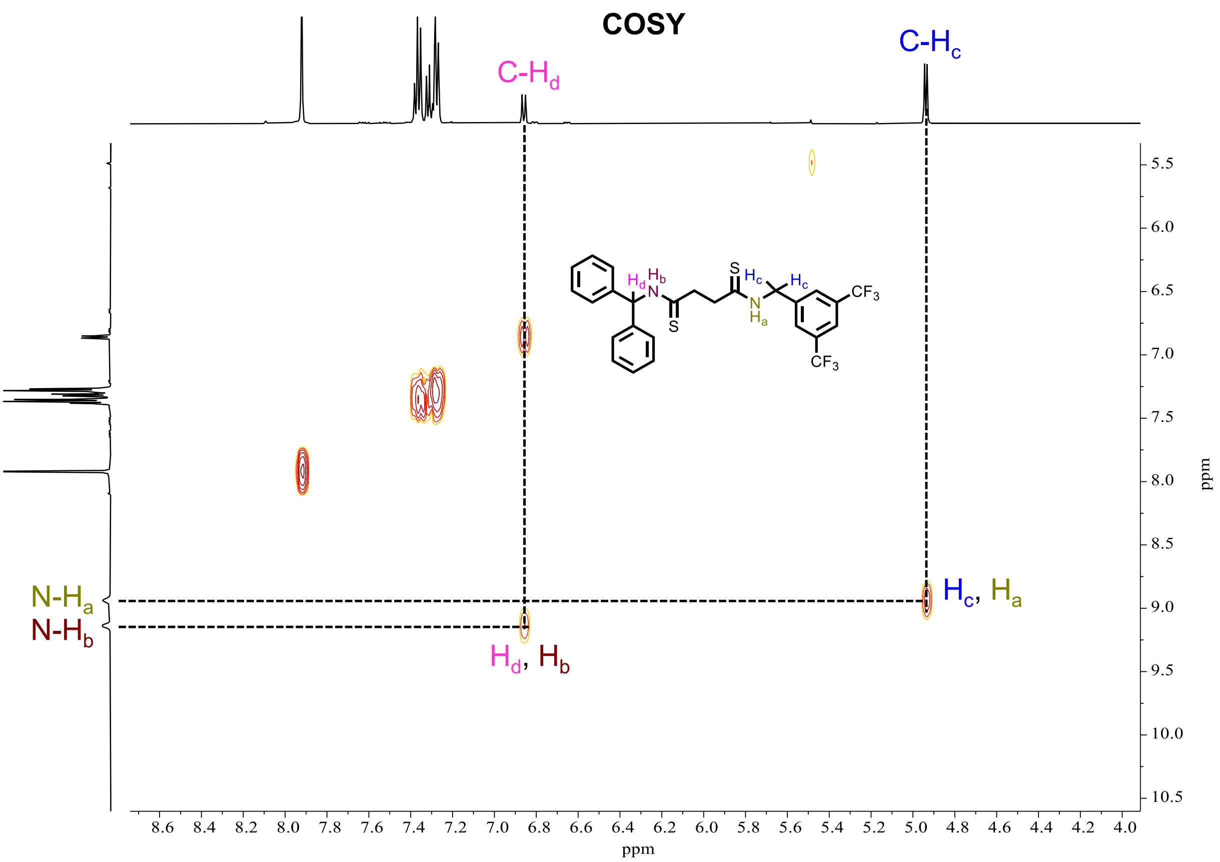

Figure S3. Structural determination of compound **8** using COSY NMR (500 MHz).

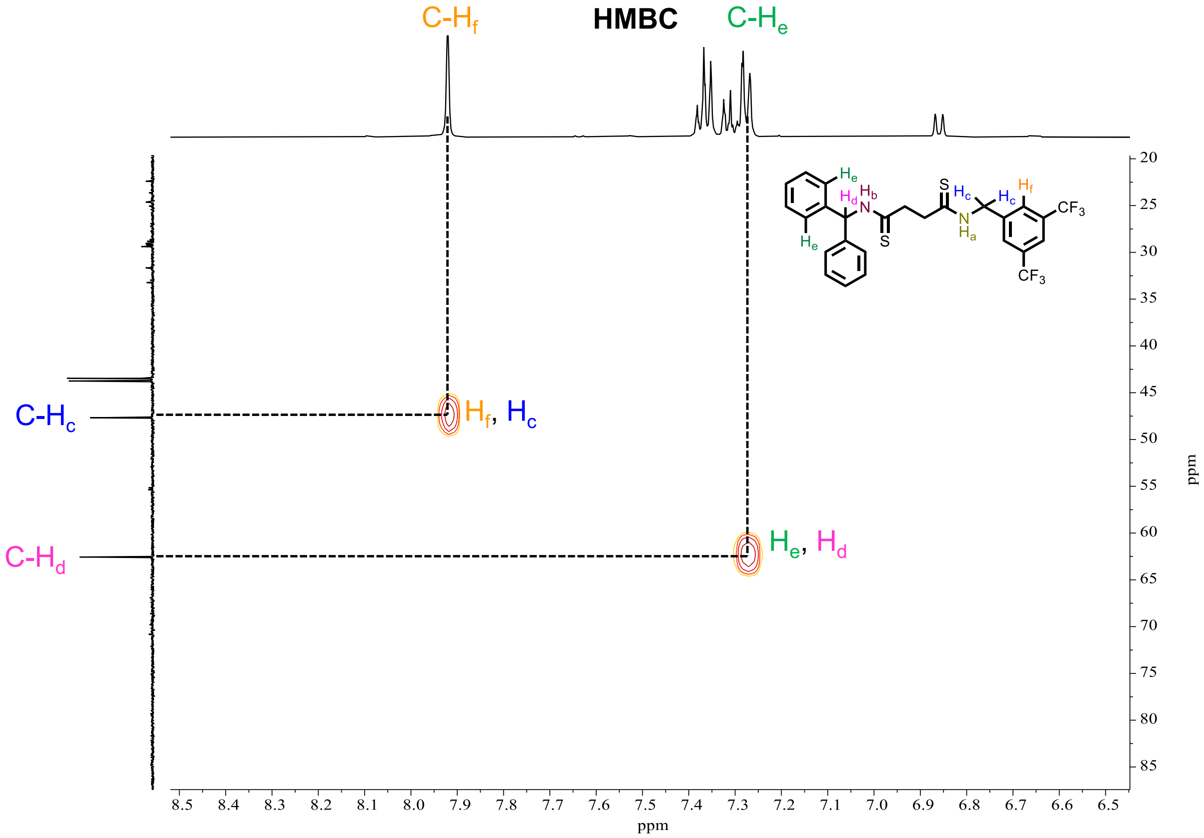

Figure S4. Structural determination of compound **8** using HMBC NMR (500 MHz).

S4 Binding Studies in Solution

^{1}H NMR titrations were carried out to determine the binding constant of all receptors towards Cl^- anions. A stock solution (5 mM) of each receptor and tetrabutyl ammonium chloride (TBACl, 500 mM) was prepared in acetonitrile-d_3. Each receptor (0.5 mL, 5 mM) was placed in a NMR tube, and the ^{1}H NMR spectrum was recorded upon incremental addition of TBACl solution. The chemical shifts of interacting N-H protons were monitored.

^{1}H NMR titrations of the receptors with other anions (Br^-, I^-, NO_3^-) were also carried out using a similar experimental protocol. TBABr, TBAI, and $TBANO_3$ salts were used as the respective anion sources. Each titration study was repeated to ensure robustness and reliability of the results. For every set of titrations performed, fresh stock solutions of receptors (0.5 mL, 5 mM) and the corresponding anion's stock solution (500 mM) were prepared in acetonitrile-d_3, thus minimizing any potential sources of variability and retaining consistency throughout experimental procedures. Similarly, chemical shifts of interacting N-H protons were recorded.

The observed chemical shifts of the N-H protons were plotted against equivalent total ($[G]_0/[H]_0$), and the binding constant values for each anion were determined using a 1:1 binding model in the BindFit v0.5 program.[2,3]

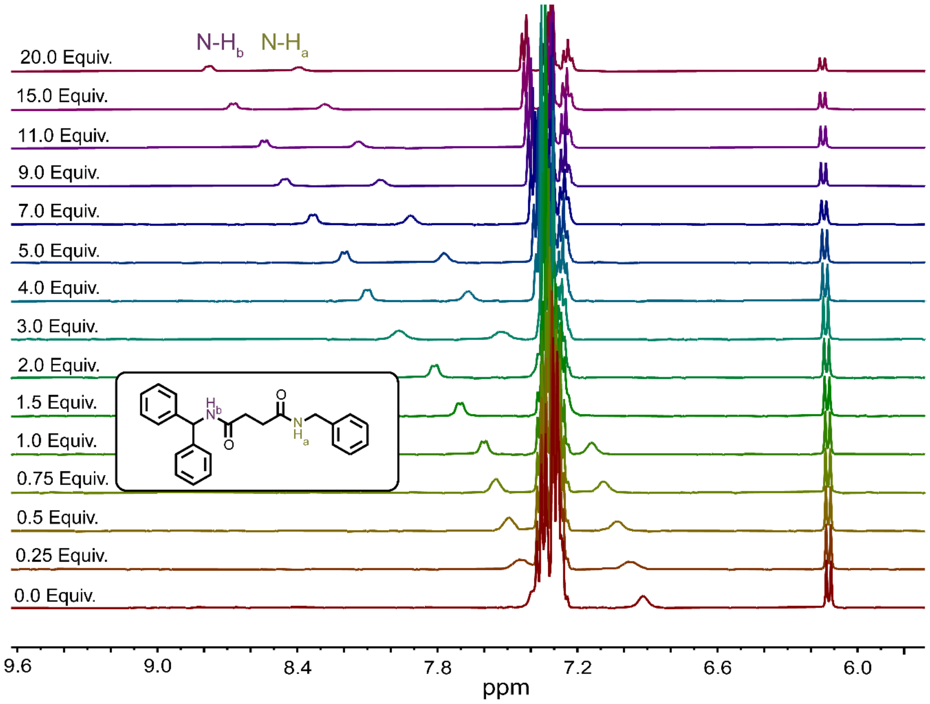

Figure S5. ^{1}H-NMR spectra showing the chemical shift (δ) of the N-H$_a$ and N-H$_b$ peaks during the titration of **1** with increasing equivalents of TBACl in acetonitrile-d_3. Representative titration spectrum from one of the duplicate experiments.

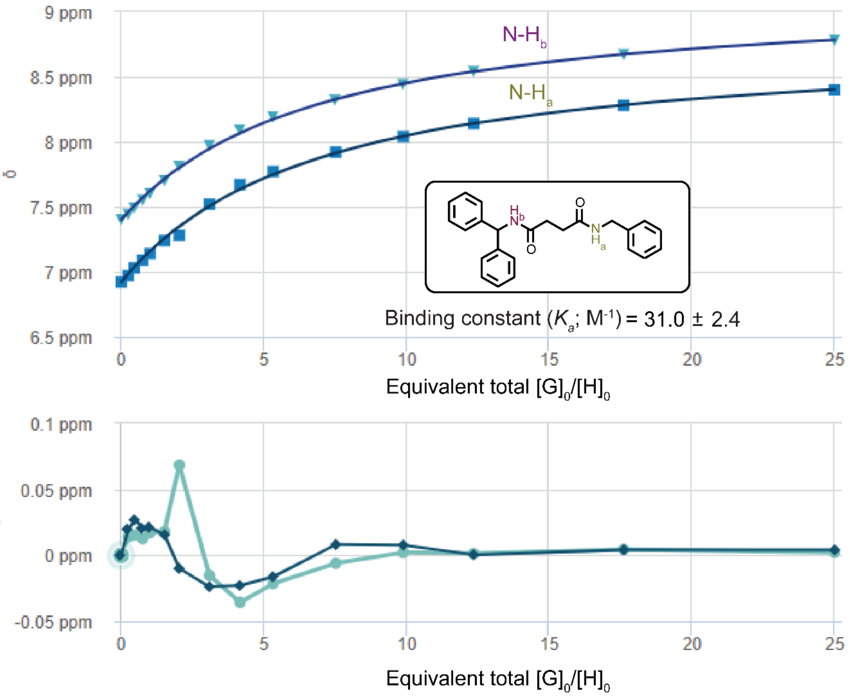

Figure S6. Chemical shift (δ) of N-H$_a$ and N-H$_b$ protons vs. equivalent total ([G]$_0$/[H]$_0$) were plotted, fitted to 1:1 binding model using BindFit v0.5 program (Nelder–Mead method). H = host (**1**) and G = guest (TBACl). Representative titration spectrum from one of the duplicate experiments.

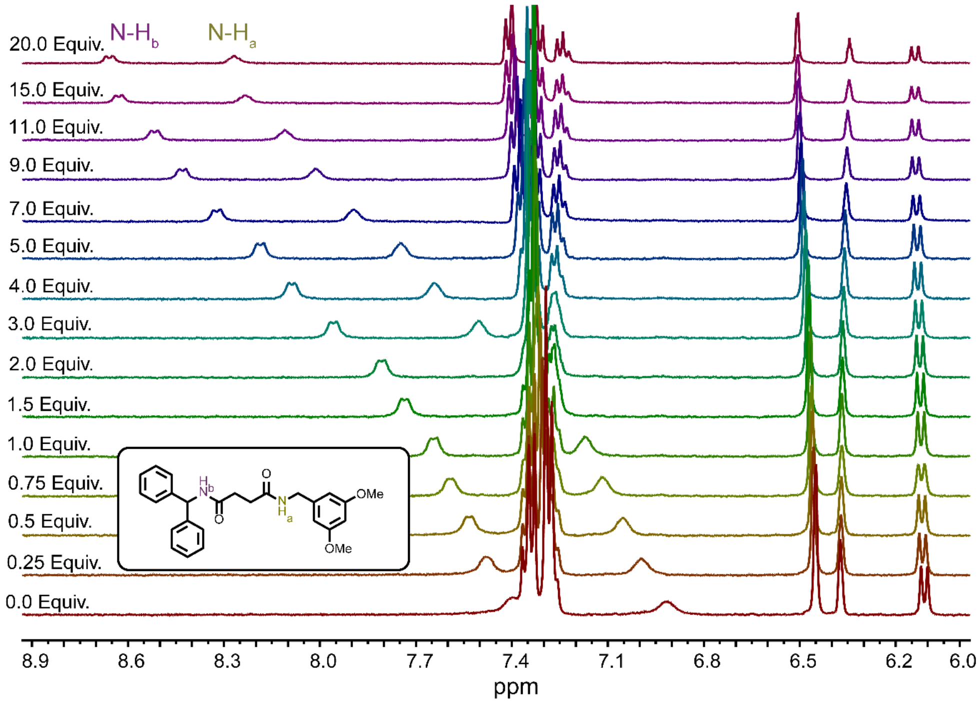

Figure S7. ^{1}H-NMR spectra showing the chemical shift (δ) of the N-H$_a$ and N-H$_b$ peaks during the titration of **2** with increasing equivalents of TBACl in acetonitrile-d_3. Representative titration spectrum from one of the duplicate experiments.

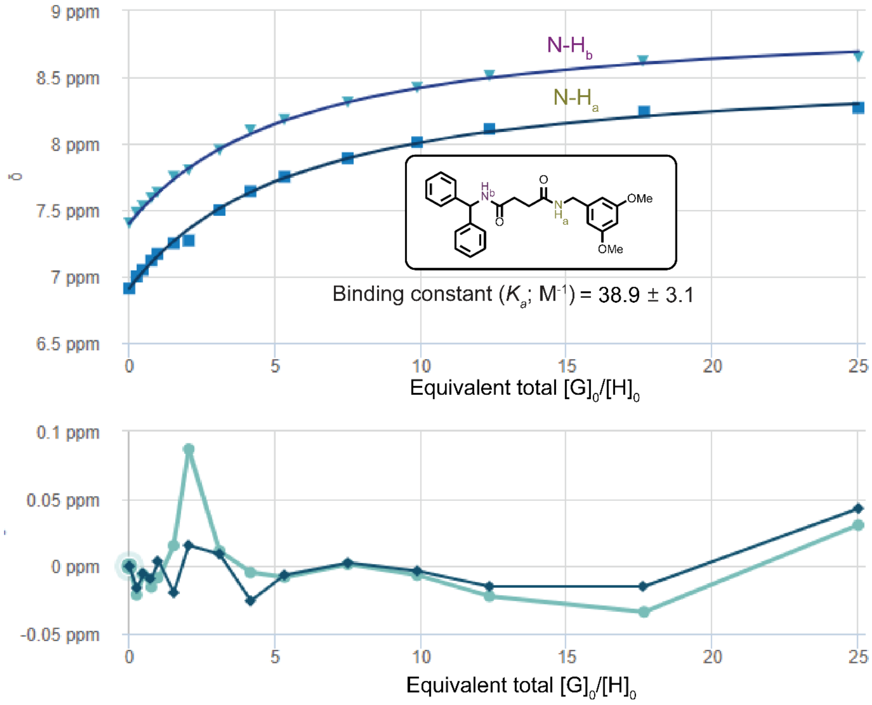

Figure S8. Chemical shift (δ) of N-H$_a$ and N-H$_b$ protons vs. equivalent total ($[G]_0/[H]_0$) were plotted, fitted to 1:1 binding model using BindFit v0.5 program (Nelder–Mead method). H = host (**2**) and G = guest (TBACl). Representative titration spectrum from one of the duplicate experiments.

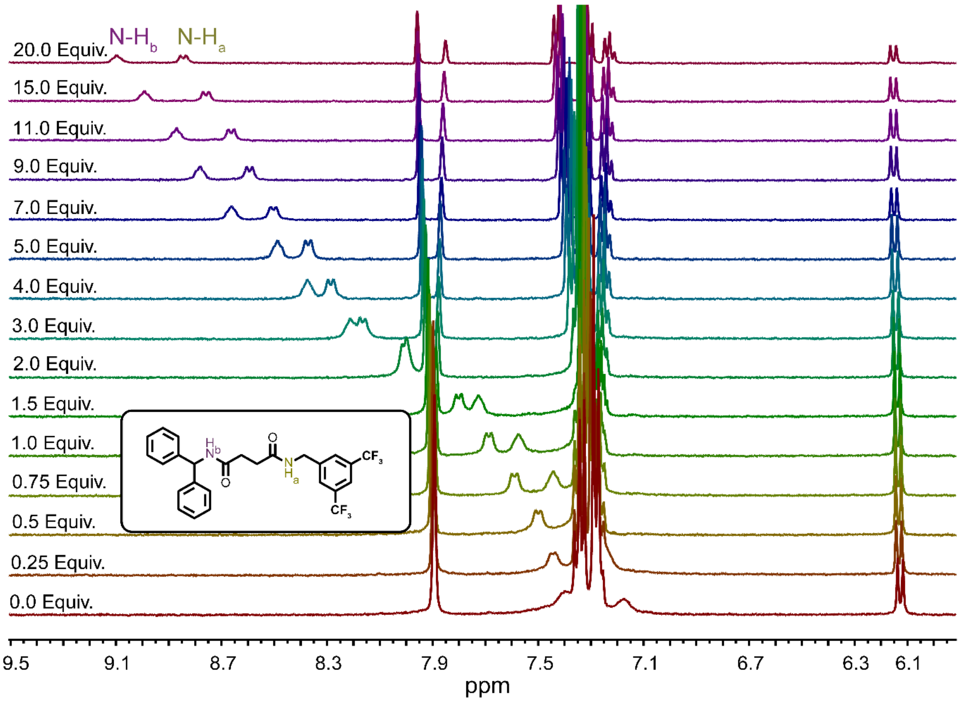

Figure S9. ^{1}H-NMR spectra showing the chemical shift (δ) of the N-H_a and N-H_b peaks during the titration of **3** with increasing equivalents of TBACl in acetonitrile-d_3. Representative titration spectrum from one of the duplicate experiments.

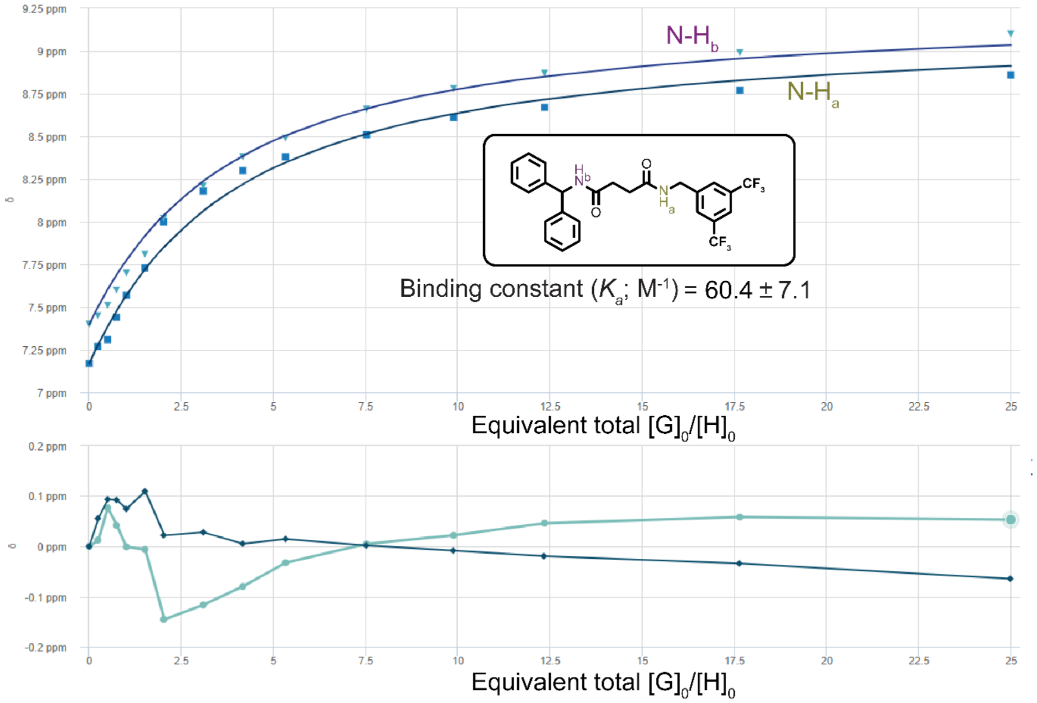

Figure S10. Chemical shift (δ) of N-H_a and N-H_b protons vs. equivalent total ($[G]_0/[H]_0$) were plotted, fitted to 1:1 binding model using BindFit v0.5 program (Nelder–Mead method). H = host (**3**) and G = guest (TBACl). Representative titration spectrum from one of the duplicate experiments.

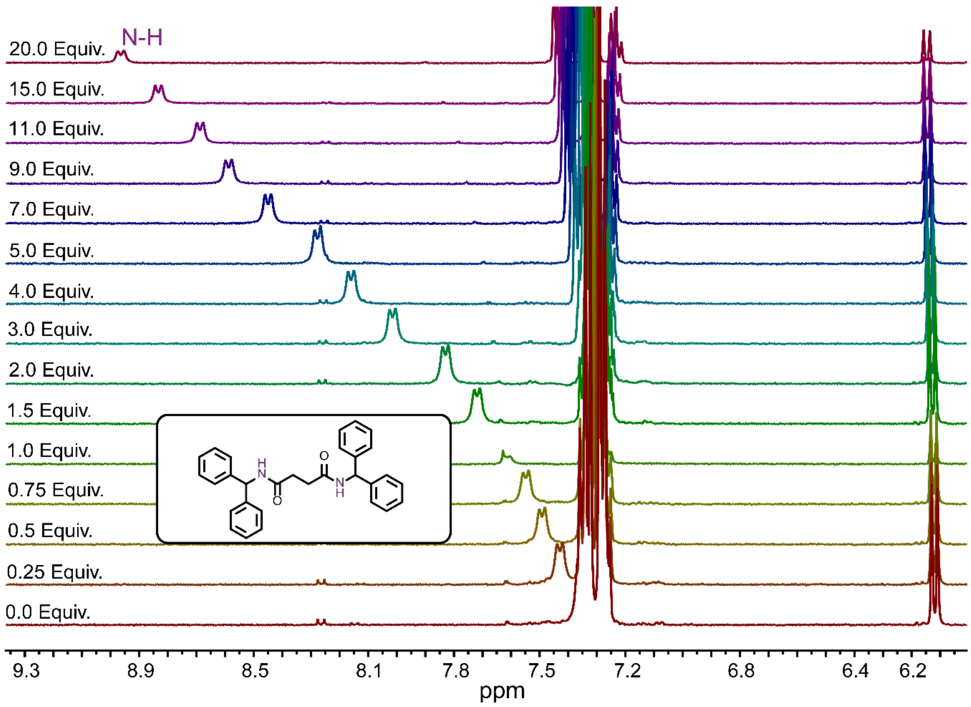

Figure S11. ^{1}H-NMR spectra showing the chemical shift (δ) of the N-H peaks during the titration of **4** with increasing equivalents of TBACl in acetonitrile-d_3. Representative titration spectrum from one of the duplicate experiments.

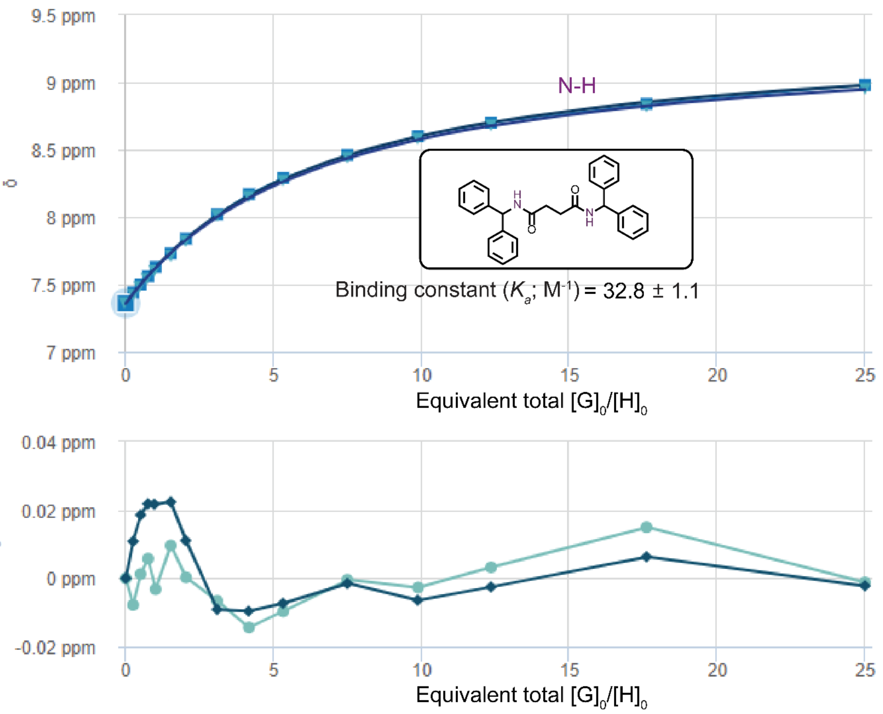

Figure S12. Chemical shift (δ) of N-H protons vs. equivalent total ($[G]_0/[H]_0$) were plotted, fitted to 1:1 binding model using BindFit v0.5 program (Nelder–Mead method). H = host (**4**) and G = guest (TBACl). Representative titration spectrum from one of the duplicate experiments.

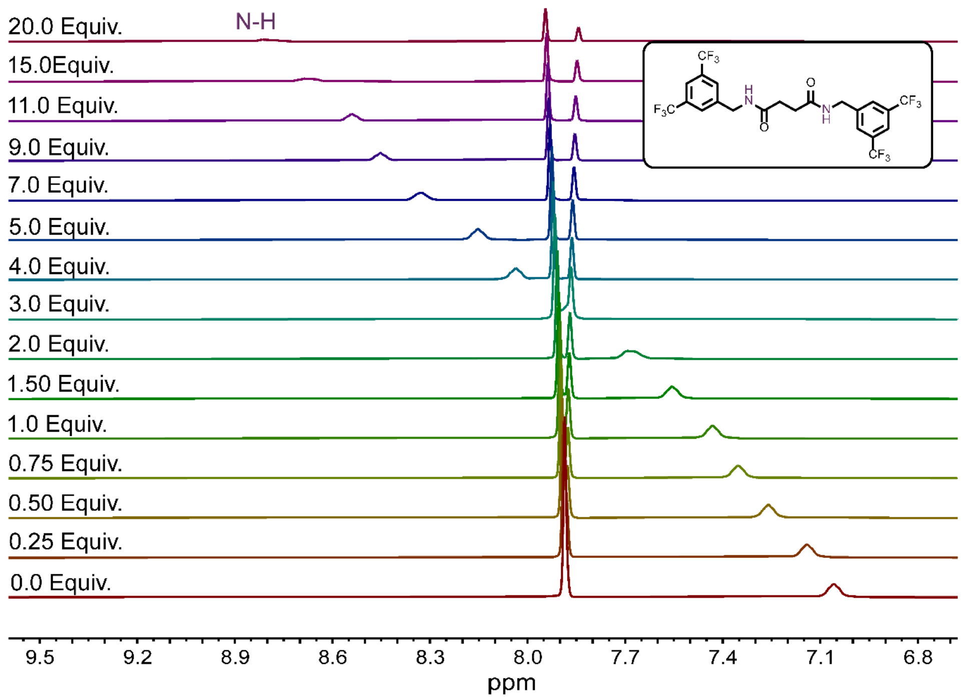

Figure S13. ^{1}H-NMR spectra showing the chemical shift (δ) of the N-H peaks during the titration of **5** with increasing equivalents of TBACl in acetonitrile-d_3. Representative titration spectrum from one of the duplicate experiments.

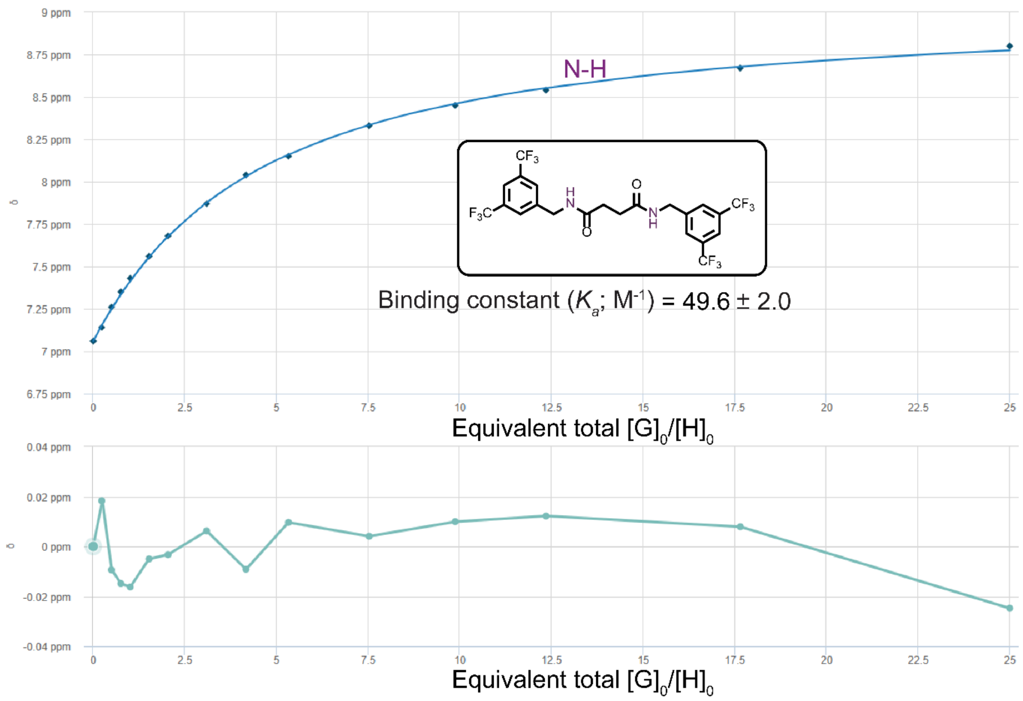

Figure S14. Chemical shift (δ) of N-H protons vs. equivalent total ($[G]_0/[H]_0$) were plotted, fitted to 1:1 binding model using BindFit v0.5 program (Nelder–Mead method). H = host (**5**) and G = guest (TBACl). Representative titration spectrum from one of the duplicate experiments.

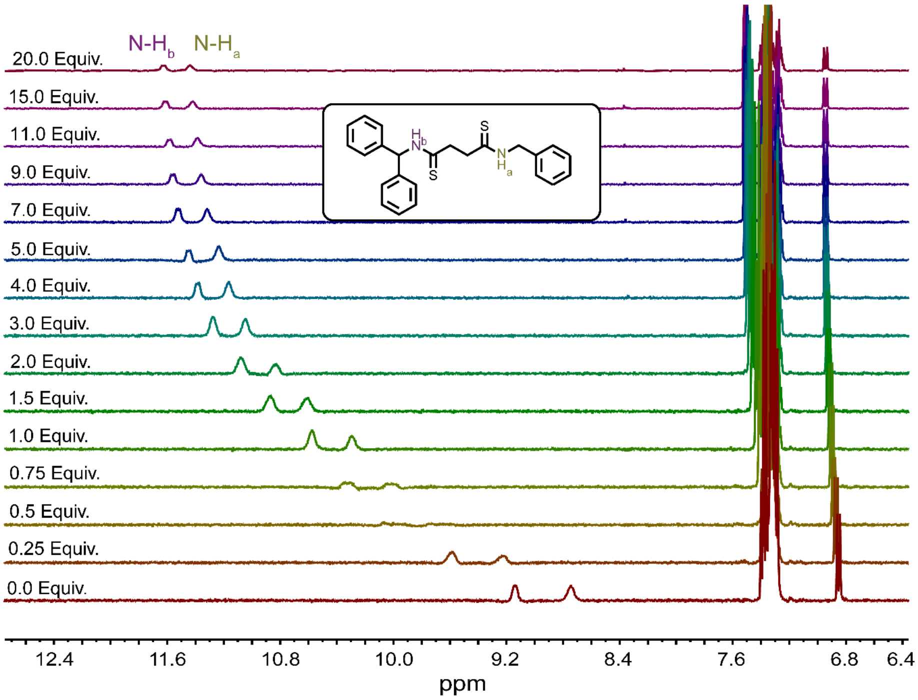

Figure S15. ^{1}H-NMR spectra showing the chemical shift (δ) of the N-H$_a$ and N-H$_b$ peaks during the titration of **6** with increasing equivalents of TBACl in acetonitrile-d_3. Representative titration spectrum from one of the duplicate experiments.

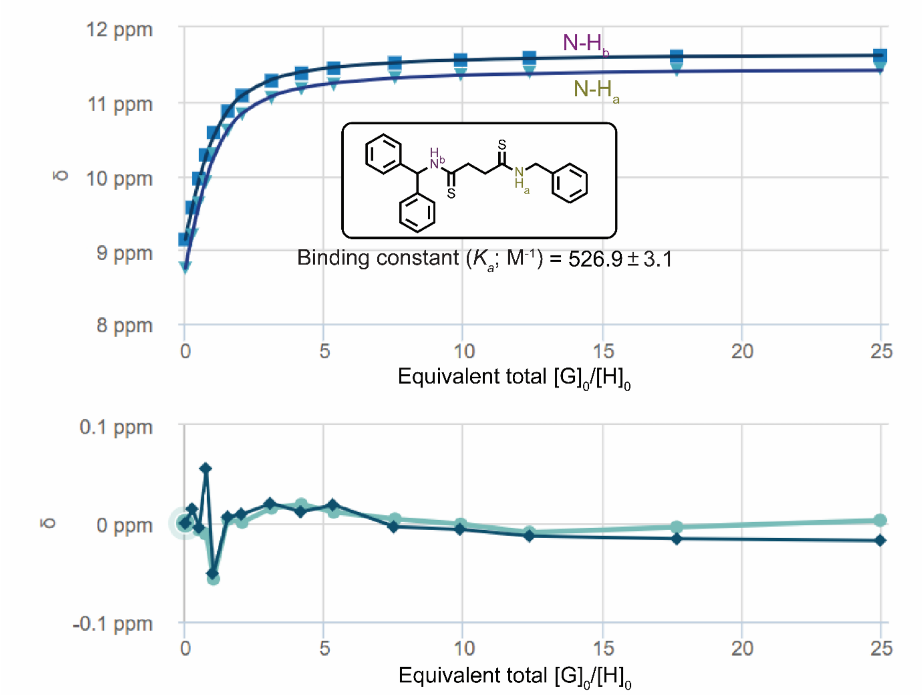

Figure S16. Chemical shift (δ) of N-H$_a$ and N-H$_b$ protons vs. equivalent total ([G]$_0$/[H]$_0$) were plotted, fitted to 1:1 binding model using BindFit v0.5 program (Nelder–Mead method). H = host (**6**) and G = guest (TBACl). Representative titration spectrum from one of the duplicate experiments.

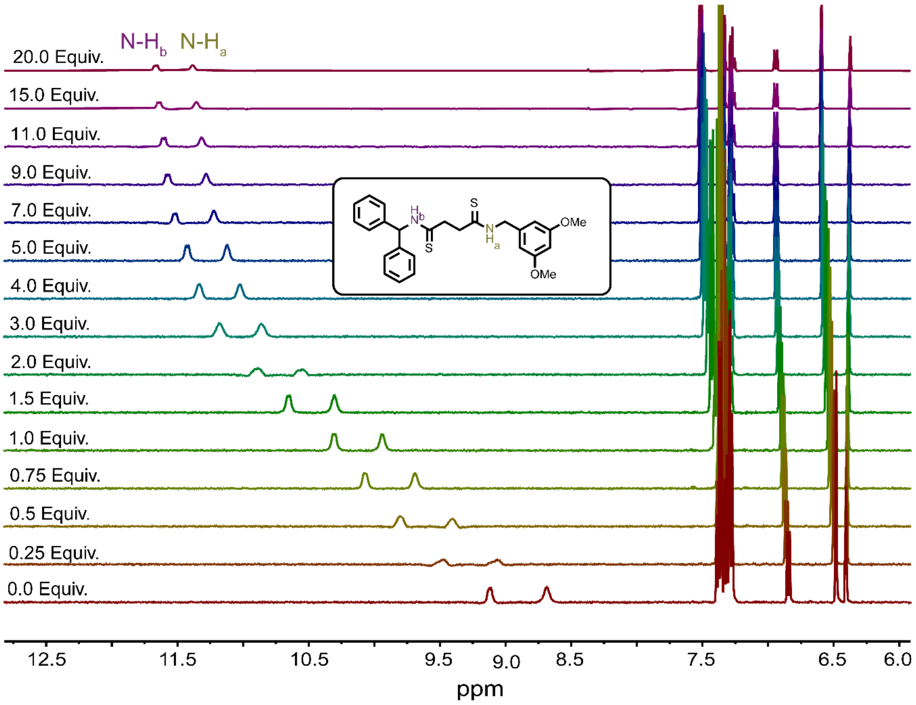

Figure S17. ^{1}H-NMR spectra showing the chemical shift (δ) of the N-H$_a$ and N-H$_b$ peaks during the titration of **7** with increasing equivalents of TBACl in acetonitrile-d_3. Representative titration spectrum from one of the duplicate experiments.

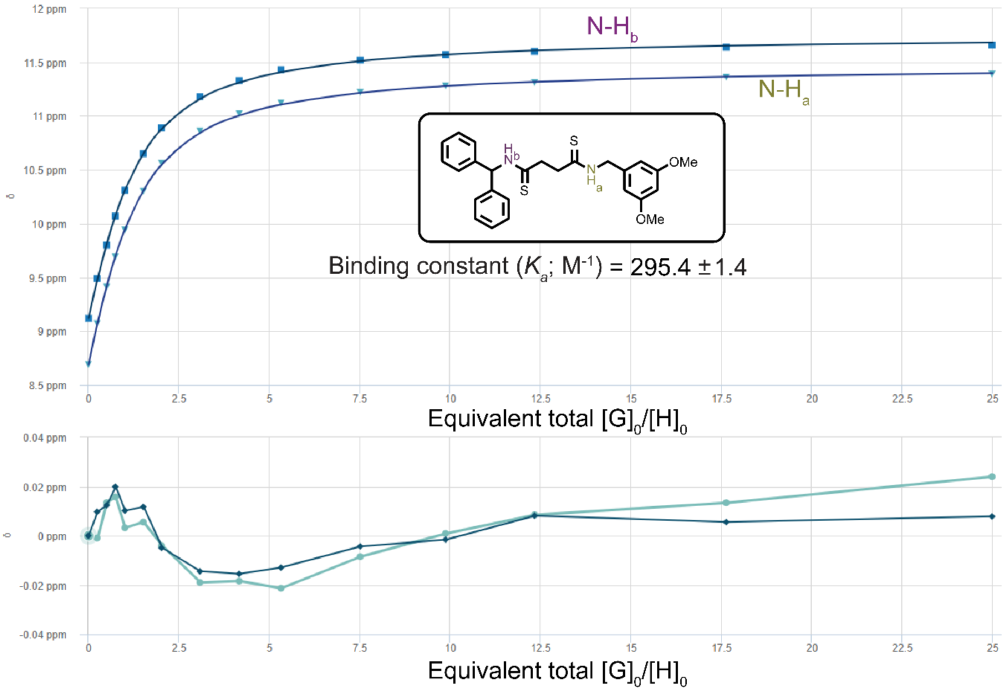

Figure S18. Chemical shift (δ) of N-H$_a$ and N-H$_b$ protons vs. equivalent total ([G]$_0$/[H]$_0$) were plotted, fitted to 1:1 binding model using BindFit v0.5 program (Nelder–Mead method). H = host (**7**) and G = guest (TBACl). Representative titration spectrum from one of the duplicate experiments.

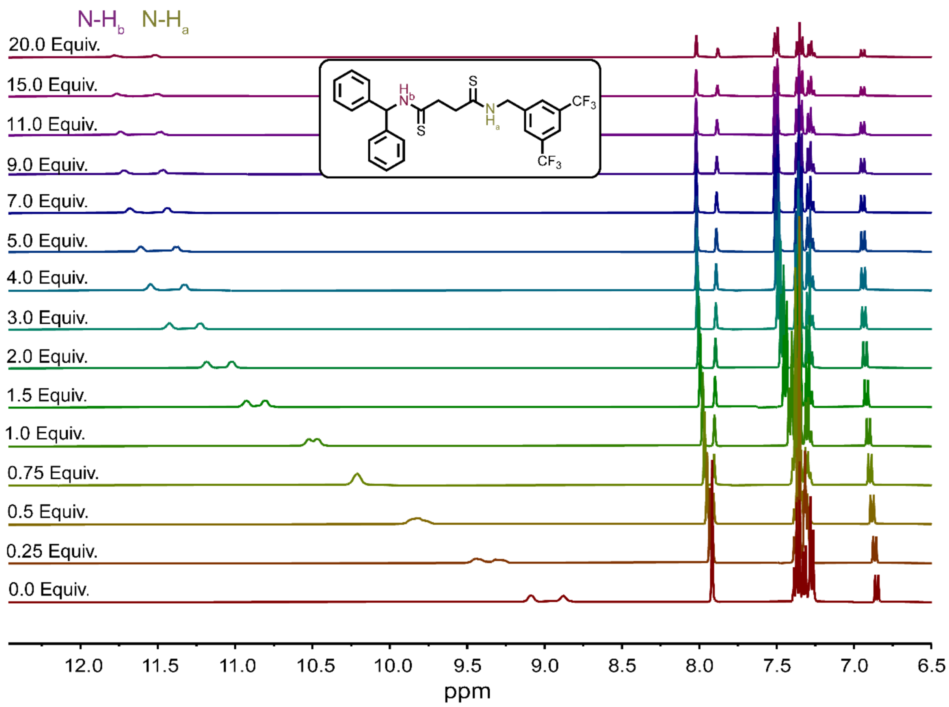

Figure S19. ^{1}H-NMR spectra showing the chemical shift (δ) of the N-H$_a$ and N-H$_b$ peaks during the titration of **8** with increasing equivalents of TBACl in acetonitrile-d_3. Representative titration spectrum from one of the duplicate experiments.

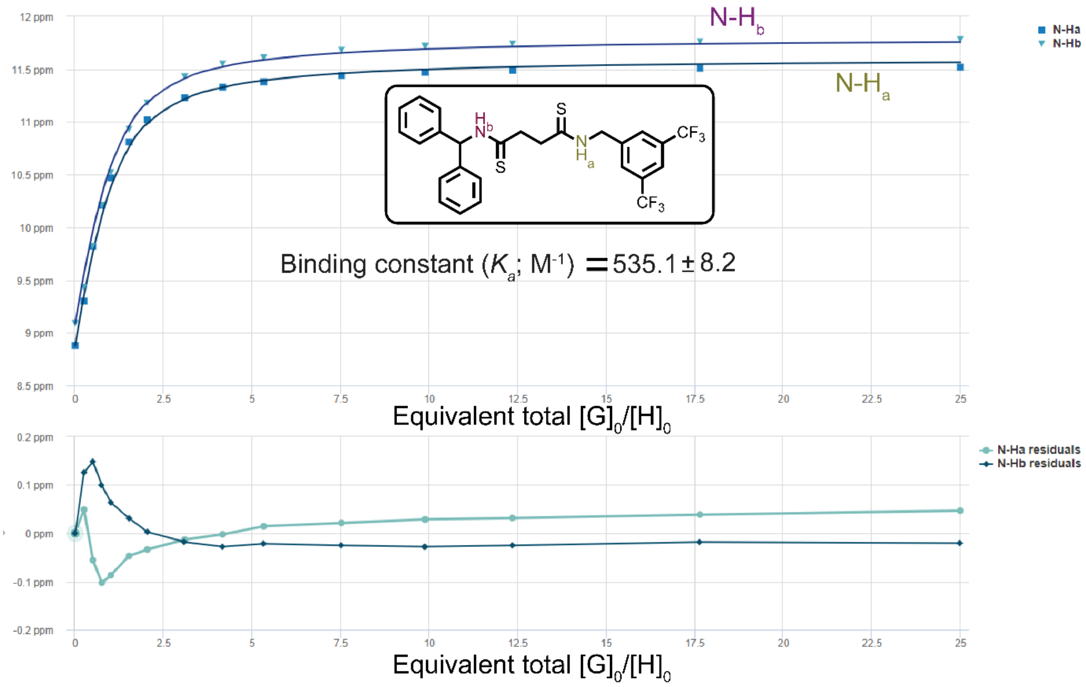

Figure S20. Chemical shift (δ) of N-H$_a$ and N-H$_b$ protons vs. equivalent total ([G]$_0$/[H]$_0$) were plotted, fitted to 1:1 binding model using BindFit v0.5 program (Nelder–Mead method). H = host (**8**) and G = guest (TBACl). Representative titration spectrum from one of the duplicate experiments.

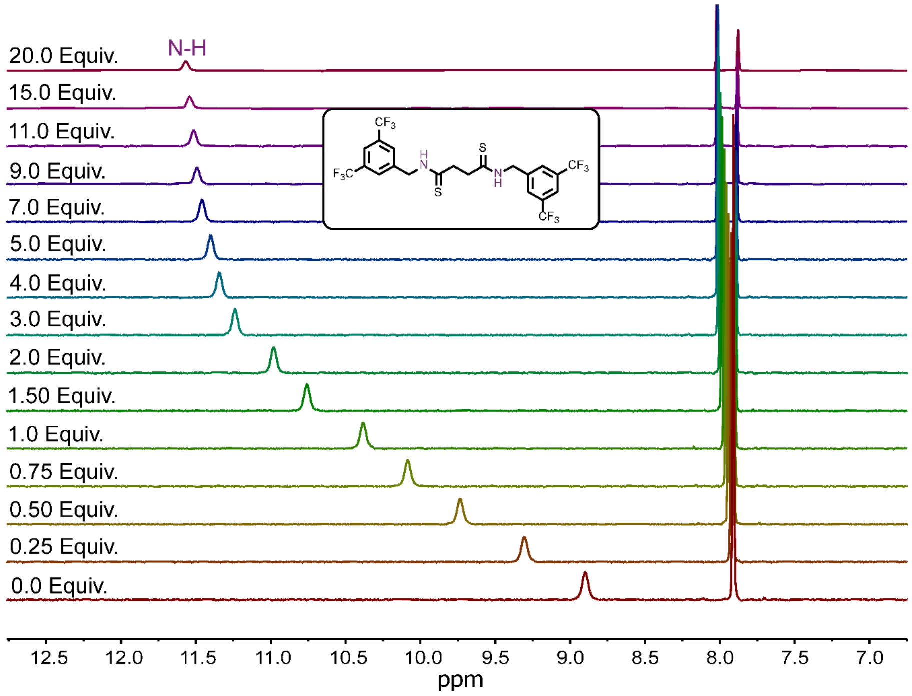

Figure S21. ^{1}H-NMR spectra showing the chemical shift (δ) of the N-H peaks during the titration of **10** with increasing equivalents of TBACl in acetonitrile-d_3. Representative titration spectrum from one of the duplicate experiments.

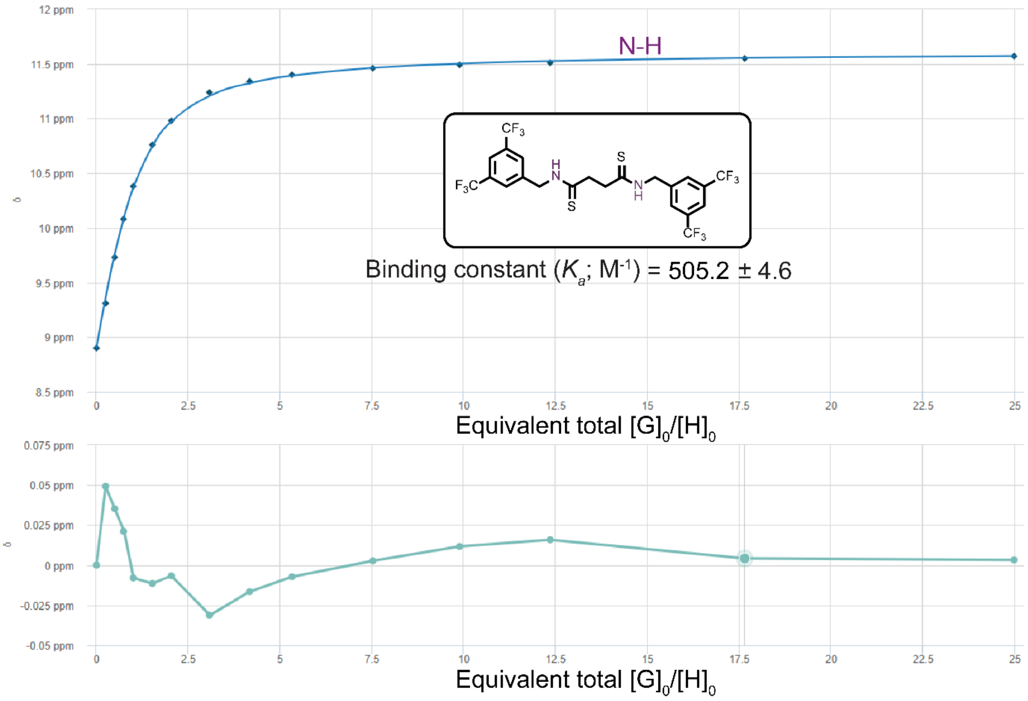

Figure S22. Chemical shift (δ) of N-H protons vs. equivalent total ($[G]_0/[H]_0$) were plotted, fitted to 1:1 binding model using BindFit v0.5 program (Nelder–Mead method). H = host (**10**) and G = guest (TBACl). Representative titration spectrum from one of the duplicate experiments.

Table S1. Binding constant (K_a; M^{-1}) values of receptors upon addition of TBACl using the 1 :1 non-cooperative model of BindFit program (Nelder–Mead method).

Receptors	K_a from Set 1 with Error	K_a from Set 2 with Error	Average K_a [b]
**1**	31.0 ± 2.4 %	29.9 ± 3.1 %	30 ± 3
**2**	38.9 ± 3.1 %	38.6 ± 3.1 %	39 ± 4
**3**	60.4 ± 7.1 %	58.3 ± 6.9 %	59 ± 6
**4**	32.8 ± 1.1 %	31.9 ± 2.1 %	32 ± 3
**5**	49.6 ± 2.0 %	49.4 ± 2.5 %	50 ± 5
**6**	526.9 ± 3.1 %	538.0 ± 4.7 %	530 ± 50
**7**	295.4 ± 1.4 %	299.4 ± 1.4 %	300 ± 30
**8**	535.1 ± 8.2 %	539.4 ± 8.0 %	540 ± 50
**9**	-[a]	-[a]	-[a]
**10**	505.2 ± 4.6 %	505.1 ± 4.8 %	510 ± 50

a. Precipitation hinders the determination of its *K* value.

b. The average binding constants (K_a) are rounded to reflect a ~10% experimental uncertainty in NMR titrations.

S5 Other fitting models for receptors 3 and 8.

Table S2. Binding constant (K_a; M^{-1}) values of receptors upon addition of TBACl using the 1 : 2 non-cooperative model of BindFit program (Nelder–Mead method).

Receptor	K_a from Set 1 with Error	K_a from Set 2 with Error	Average K_a [c]
**1**	K_{11} = 3956.4 ± 142.9 %<br>K_{12} = 31.9 ± 3.2 %	K_{11} = 4964.4 ± 260.6 %<br>K_{12} = 33.3 ± 4.2 %	K_{11} = 4500 ± 450<br>K_{12} = 33 ± 3
**2**	-[a]	-[a]	-[a]
**3**	K_{11} = 1388.9 ± 79.2 %<br>K_{12} = 65.5 ± 8.3 %	K_{11} = 2720.1 ± 148.5 %<br>K_{12} = 65.6 ± 8.1 %	K_{11} = 2100 ± 210<br>K_{12} = 66 ± 7
**4**	K_{11} = 2774.2 ± 32.6 %<br>K_{12} = 30.5 ± 1.2 %	K_{11} = 919.9 ± 37.2 %<br>K_{12} = 27.7 ± 3.6 %	K_{11} = 1800 ± 180<br>K_{12} = 29 ± 3
**5**	-[a]	-[a]	-[a]
**6**	K_{11} = 1000.3 ± 4.4 %<br>K_{12} = 40.1 ± 9.8 %	K_{11} = 1010.6 ± 4.8 %<br>K_{12} = 43.5 ± 10.3 %	K_{11} = 1000 ± 100<br>K_{12} = 42 ± 4
**7**	K_{11} = 445.3 ± 0.9 %<br>K_{12} = 60.2 ± 2.3 %	K_{11} = 538.4 ± 1.2 %<br>K_{12} = 75.7 ± 2.2 %	K_{11} = 490 ± 50<br>K_{12} = 68 ± 7
**8**	K_{11} = 17575.9 ± 38.9 %<br>K_{12} = 275.4 ± 9.0 %	K_{11} =14579.1 ± 35.1 %<br>K_{12} = 259.3 ± 9.2 %	K_{11} = 16000 ± 1600<br>K_{12} = 270 ± 27
**9**	-[b]	-[b]	-[b]
**10**	K_{11} = 4169.6 ± 10.4 %<br>K_{12} = 215.6 ± 6.0 %	K_{11} = 3756.8 ± 12.9 %<br>K_{12} = 211.7 ± 8.0 %	K_{11} = 4000 ± 400<br>K_{12} = 210 ± 21

a. The data does not fit with this model.

b. Precipitation hinders the determination of its *K* value.

c. The average binding constants (K_a) are rounded to reflect a ~10% experimental uncertainty in NMR titrations.

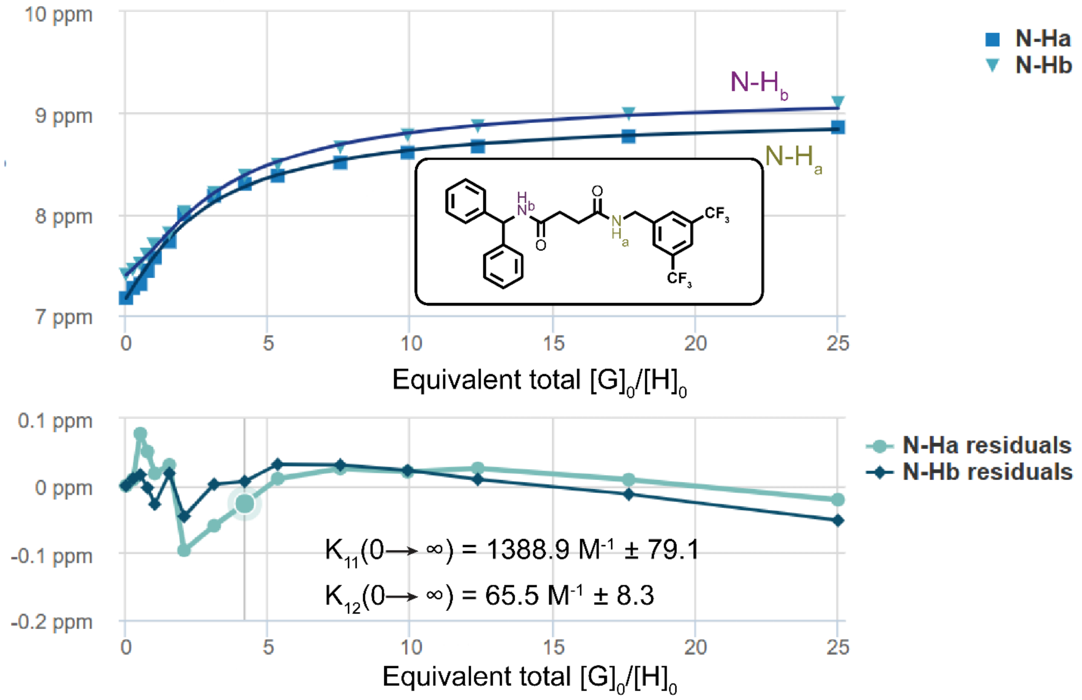

Figure S23. Chemical shift (δ) of N-H$_a$ and N-H$_b$ protons vs. equivalent total ($[G]_0/[H]_0$) were plotted, fitted to 1:2 binding model using BindFit v0.5 program (Nelder–Mead method). H = host (**3**) and G = guest (TBACl). Representative titration spectrum from one of the duplicate experiments.

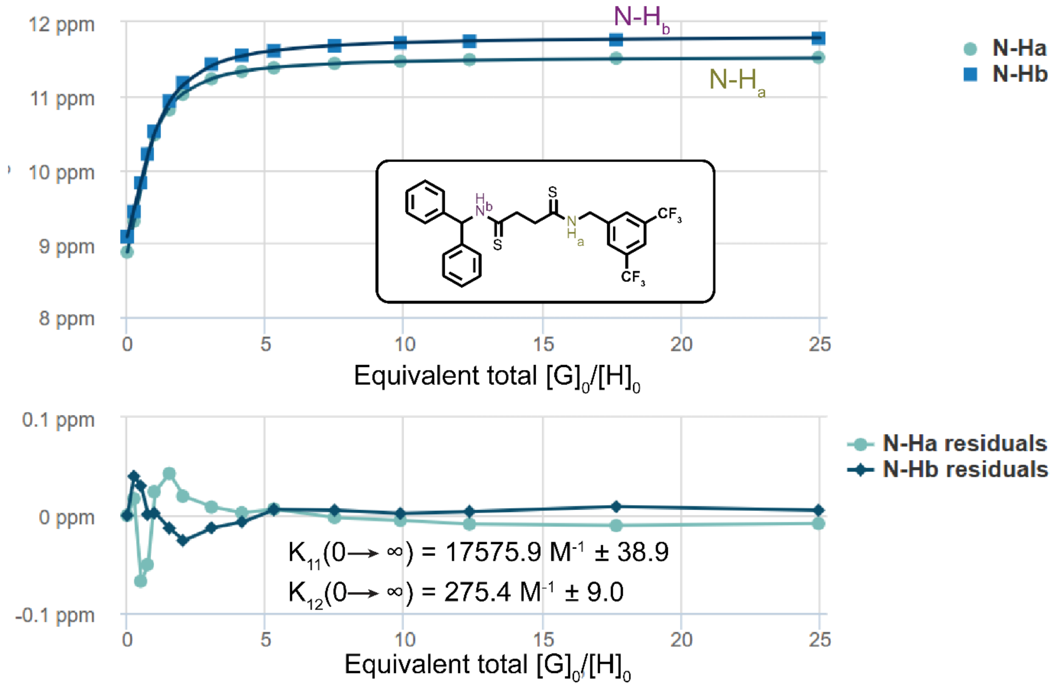

Figure S24. Chemical shift (δ) of N-H$_a$ and N-H$_b$ protons vs. equivalent total ($[G]_0/[H]_0$) were plotted, fitted to 1:2 binding model using BindFit v0.5 program (Nelder–Mead method). H = host (**8**) and G = guest (TBACl). Representative titration spectrum from one of the duplicate experiments.

Table S3. Binding constant (K_a; M^{-1}) values of receptors upon addition of TBACl using the 2 : 1 non-cooperative model of BindFit program (Nelder–Mead method).

Receptor	K_a from Set 1 with Error	K_a from Set 2 with Error	Average K_a [c]
**1**	K_{11} = 86.9 ± 11.4 %<br>K_{21} = 123.9 ± 16.2 %	K_{11} = 90.9 ± 11.9 %<br>K_{21} = 111.5 ± 16.2 %	K_{11} = 89 ± 9<br>K_{21} = 118 ± 12
**P2**	K_{11} = 0.3 ± 8.3 %<br>K_{21} = 3612.3 ± 4.6 %	K_{11} = 0.4 ± 7.7 %<br>K_{21} = 2923.0 ± 4.2 %	K_{11} = 0.3 ± 0.03<br>K_{21} = 3300 ± 330
**3**	K_{11} = 213.5 ± 16.9 %<br>K_{21} = 152.5 ± 21.1 %	K_{11} = 235.3 ± 17.9 %<br>K_{21} = 199.6 ± 21.5 %	K_{11} = 224 ± 22<br>K_{21} = 176 ± 18
**4**	K_{11} = 70.6 ± 5.1 %<br>K_{21} = 77.1 ± 8.8 %	K_{11} = 54.3 ± 18.8 %<br>K_{21} = 37.1 ± 43.5 %	K_{11} = 62 ± 6<br>K_{21} = 57 ± 6
**5**	K_{11} = 92.9 ± 39.9 %<br>K_{21} = 130.30 ± 73.7 %	K_{11} = 62.6 ± 27.9 %<br>K_{21} = 53.0 ± 99.7 %	K_{11} = 78 ± 8<br>K_{21} = 92 ± 9
**6**	K_{11} = 844.2 ± 6.8 %<br>K_{21} = 162.6 ± 17.7 %	K_{11} = 855.0 ± 10.2 %<br>K_{21} = 166.8 ± 25.65 %	K_{11} = 850 ± 85<br>K_{21} = 165 ± 17
**7**	-[a]	-[a]	-[a]
**8**	K_{11} = 1272.8 ± 8.8 %<br>K_{21} = 157.9 ± 13.9 %	K_{11} = 1254.3 ± 9.2 %<br>K_{21} = 162.9 ± 14.9 %	K_{11} = 1300 ± 130<br>K_{21} = 160 ± 16
**9**	-[b]	-[b]	-[b]
**10**	K_{11} = 762.6 ± 9.1 %<br>K_{21} = 29.4 ± 26.1 %	K_{11} = 759.7 ± 12.1 %<br>K_{21} = 29.7 ± 34.9 %	K_{11} = 761 ± 76<br>K_{21} = 30 ± 3

a. The data does not fit with this model.
b. Precipitation hinders the determination of its K value.
c. The average binding constants (K_a) are rounded to reflect a ~10% experimental uncertainty in NMR titrations.

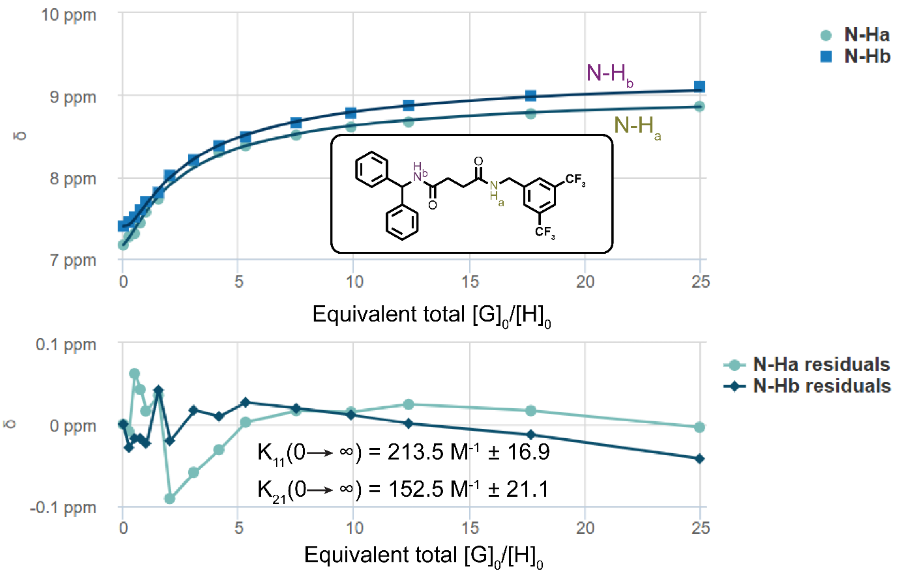

Figure S25. Chemical shift (δ) of N-H_a and N-H_b protons vs. equivalent total ($[G]_0/[H]_0$) were plotted, fitted to 2:1 binding model using BindFit v0.5 program (Nelder–Mead method). H = host (**3**) and G = guest (TBACl). Representative titration spectrum from one of the duplicate experiments.

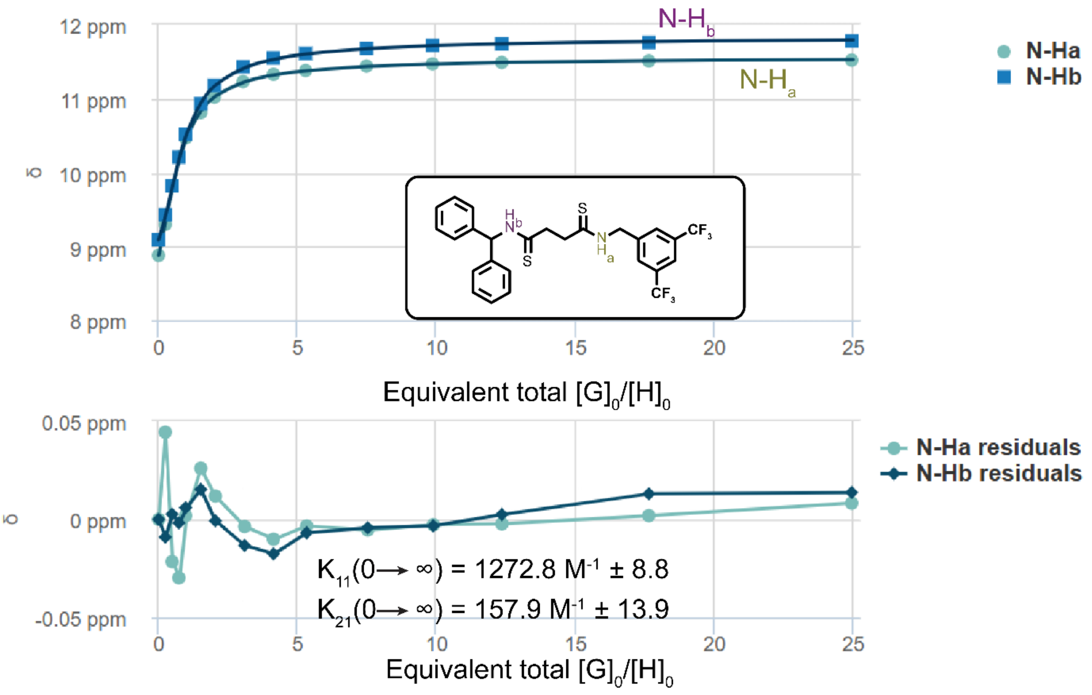

Figure S26. Chemical shift (δ) of N-H$_a$ and N-H$_b$ protons vs. equivalent total ($[G]_0/[H]_0$) were plotted, fitted to 2:1 binding model using BindFit v0.5 program (Nelder–Mead method). H = host (**8**) and G = guest (TBACl). Representative titration spectrum from one of the duplicate experiments.

S6 Anion Selectivity

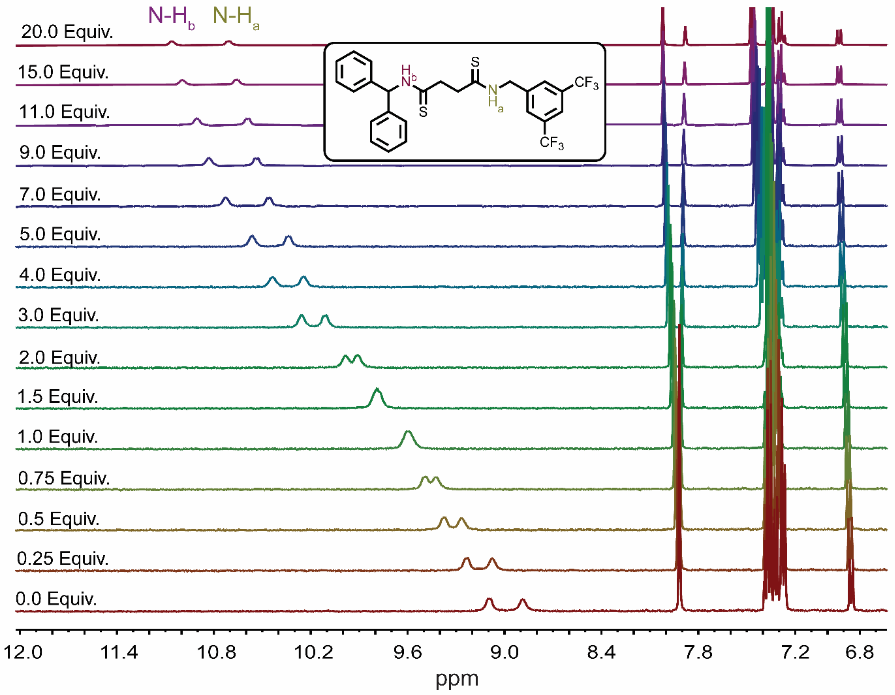

Figure S27. ^{1}H-NMR spectra showing the chemical shift (δ) of the N-H$_a$ and N-H$_b$ peaks during the titration of **8** with increasing equivalents of TBABr in acetonitrile-d_3. Representative titration spectrum from one of the duplicate experiments.

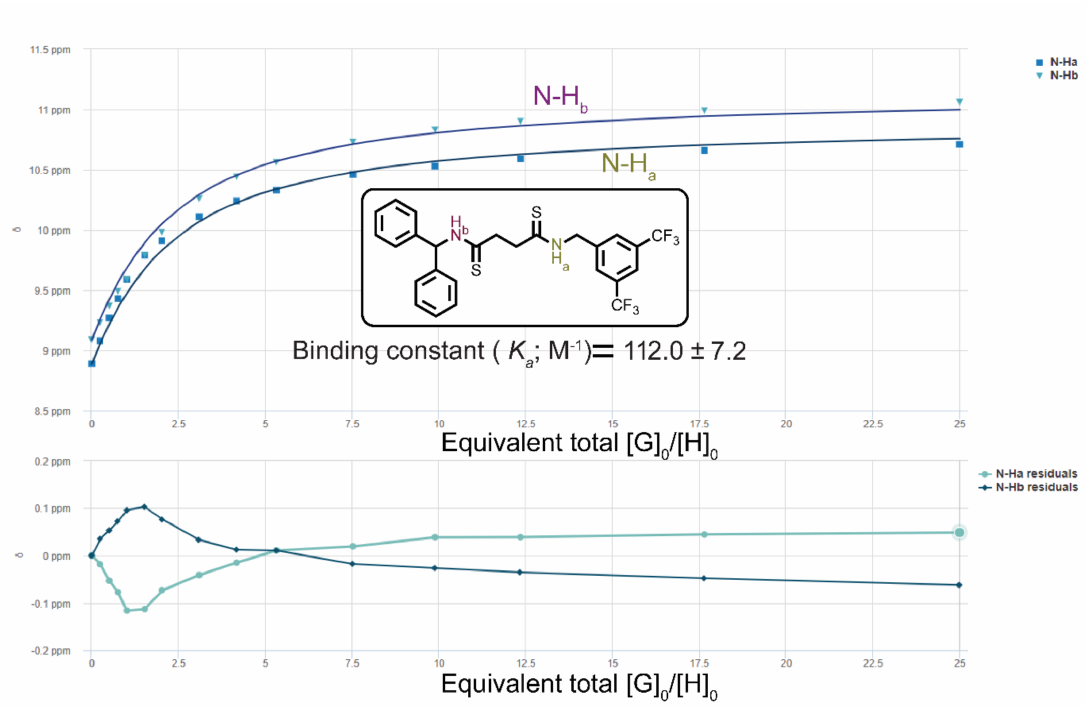

Figure S28. Chemical shift (δ) of N-H$_a$ and N-H$_b$ protons vs. equivalent total ($[G]_0/[H]_0$) were plotted, fitted to 1:1 binding model using BindFit v0.5 program (Nelder–Mead method). H = host (**8**) and G = guest (TBABr). Representative titration spectrum from one of the duplicate experiments.

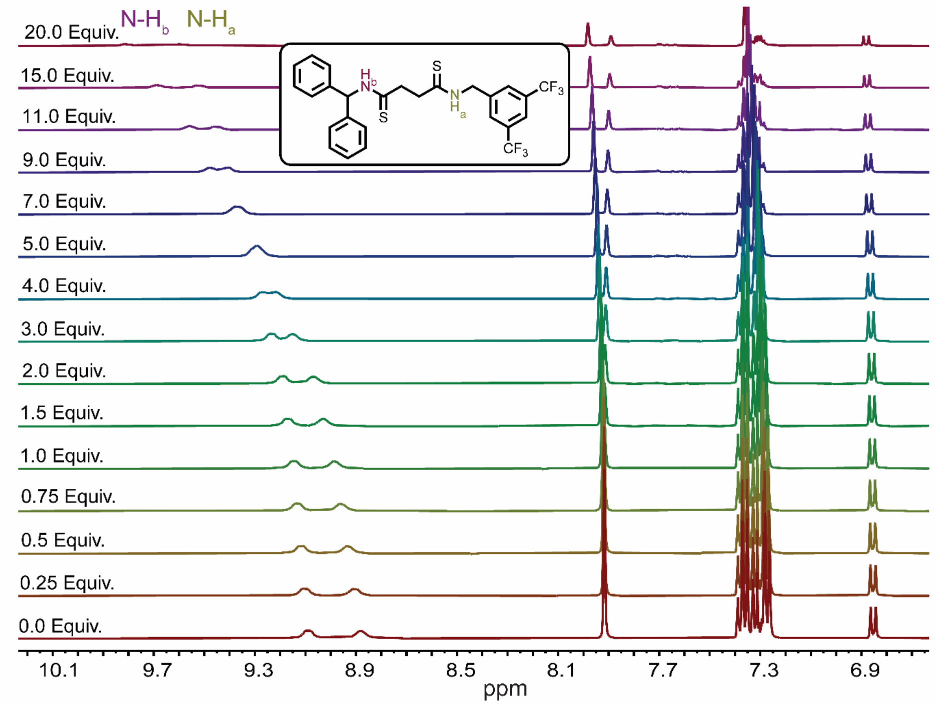

Figure S29. ^{1}H-NMR spectra showing the chemical shift (δ) of the N-H$_a$ and N-H$_b$ peaks during the titration of **8** with increasing equivalents of TBAI in acetonitrile-d_3. Representative titration spectrum from one of the duplicate experiments.

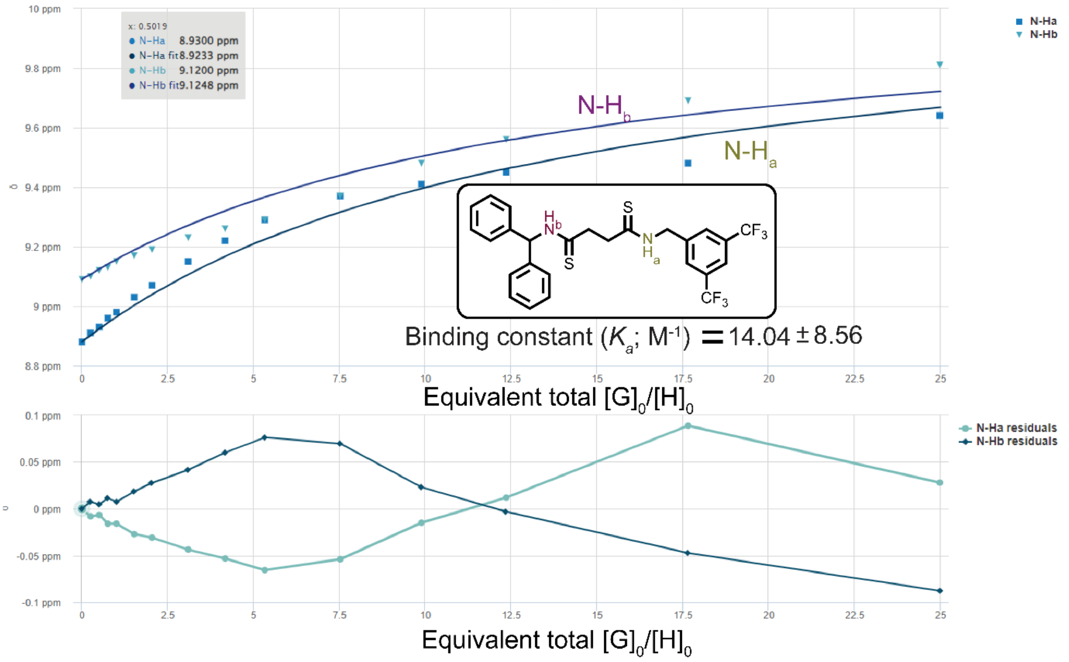

Figure S30. Chemical shift (δ) of N-H$_a$ and N-H$_b$ protons vs. equivalent total ($[G]_0/[H]_0$) were plotted, fitted to 1:1 binding model using BindFit v0.5 program (Nelder–Mead method). H = host (**8**) and G = guest (TBAI). Representative titration spectrum from one of the duplicate experiments.

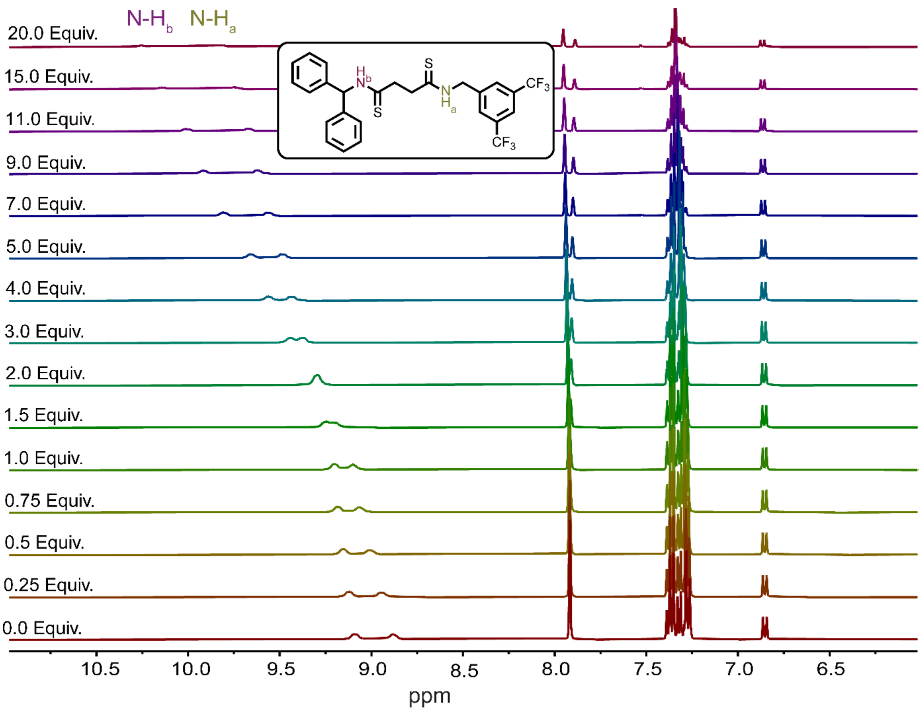

Figure S31. ^{1}H-NMR spectra showing the chemical shift (δ) of the N-H$_a$ and N-H$_b$ peaks during the titration of **8** with increasing equivalents of $TBANO_3$ in acetonitrile-*d*$_3$. Representative titration spectrum from one of the duplicate experiments.

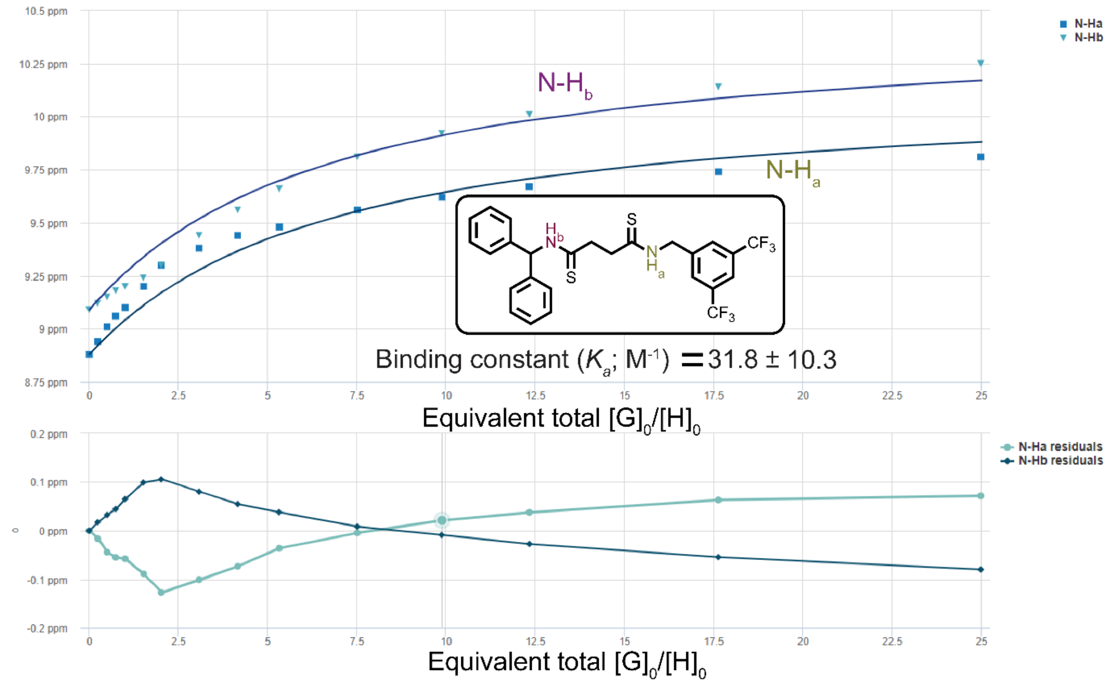

Figure S32. Chemical shift (δ) of N-H$_a$ and N-H$_b$ protons vs. equivalent total ($[G]_0/[H]_0$) were plotted, fitted to 1:1 binding model using BindFit v0.5 program (Nelder–Mead method). H = host (**8**) and G = guest ($TBANO_3$). Representative titration spectrum from one of the duplicate experiments.

Table S4. Binding constant value of compound **8** towards different anions (Cl^-, Br^-, I^- and NO_3^-) using the 1:1 binding model of BindFit v0.5 program. This is the average K_a calculated from two independent titrations for each anion.

**Receptors**	**K_a (M^{-1}) for TBACl**	**K_a (M^{-1}) for TBABr**	**K_a (M^{-1}) for TBAI**	**K_a (M^{-1}) for $TBANO_3$**
**8**	540 ± 54	112 ± 11	15 ± 2	32 ± 3

S7 Mass Spectrometric Study

Stock solutions (5 mM) of the receptors and TBACl (500 mM) were prepared in spectroscopy-grade acetonitrile. The solutions were mixed to provide a final TBACl concentration of approximately 10 equivalents relative/with respect to each receptor, followed by further dilution with spectroscopy-grade acetonitrile. The diluted samples were analyzed by mass spectroscopic analysis in negative ion mode, where it was

electro-sprayed at a flow rate of 400 µL/min with a capillary voltage of 1.0 kV. ESI-MS data confirms the existence of a chloride-encapsulated complex.[4]

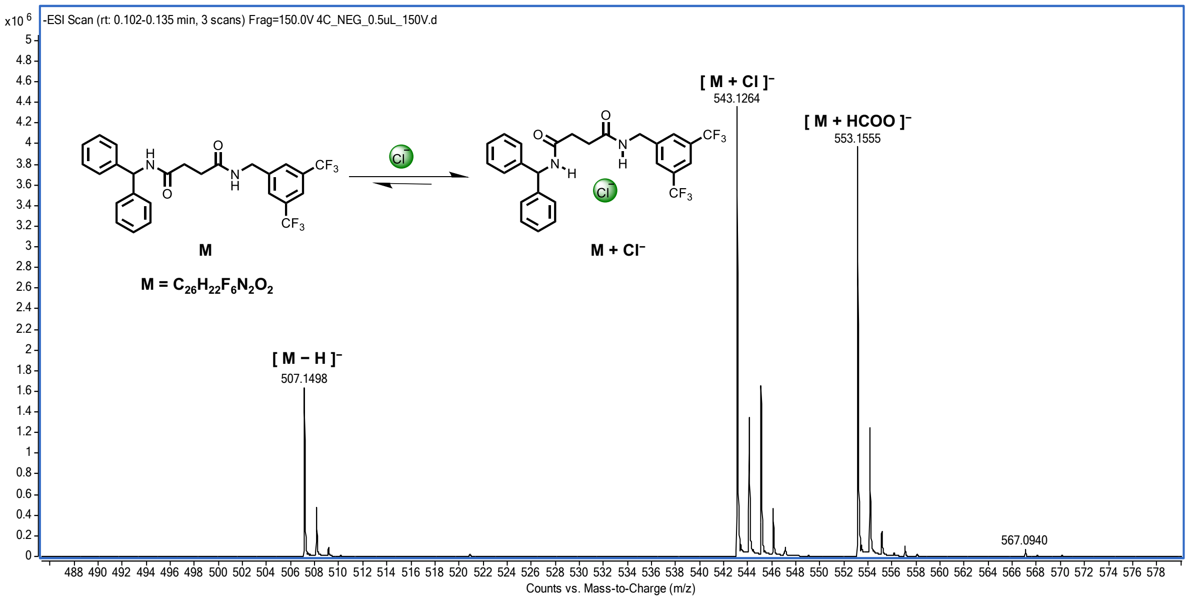

Figure S33. Negative mode ESI-MS spectrum of mixture of **3** and TBACl in acetonitrile.

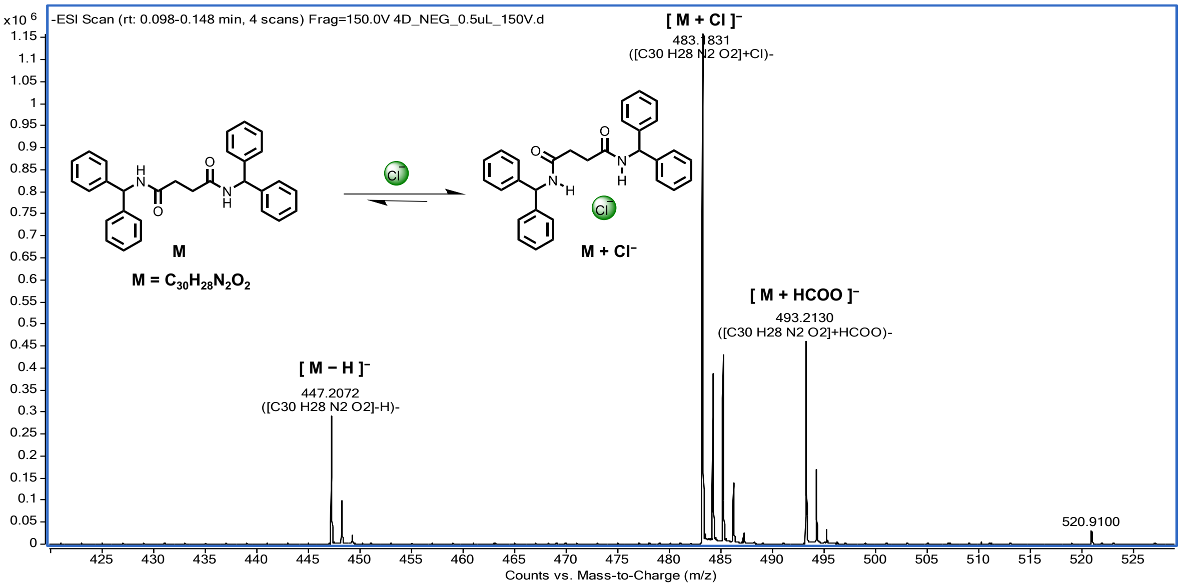

Figure S34. Negative mode ESI-MS spectrum of mixture of **4** and TBACl in acetonitrile.

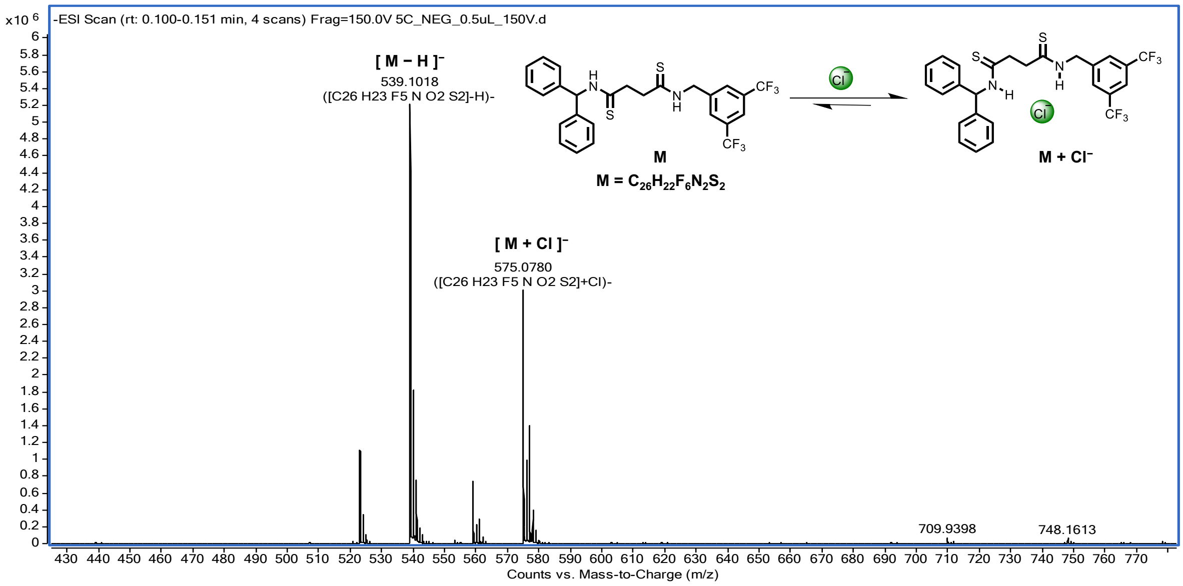

Figure S35. Negative mode ESI-MS spectrum of mixture of **8** and TBACl in acetonitrile.

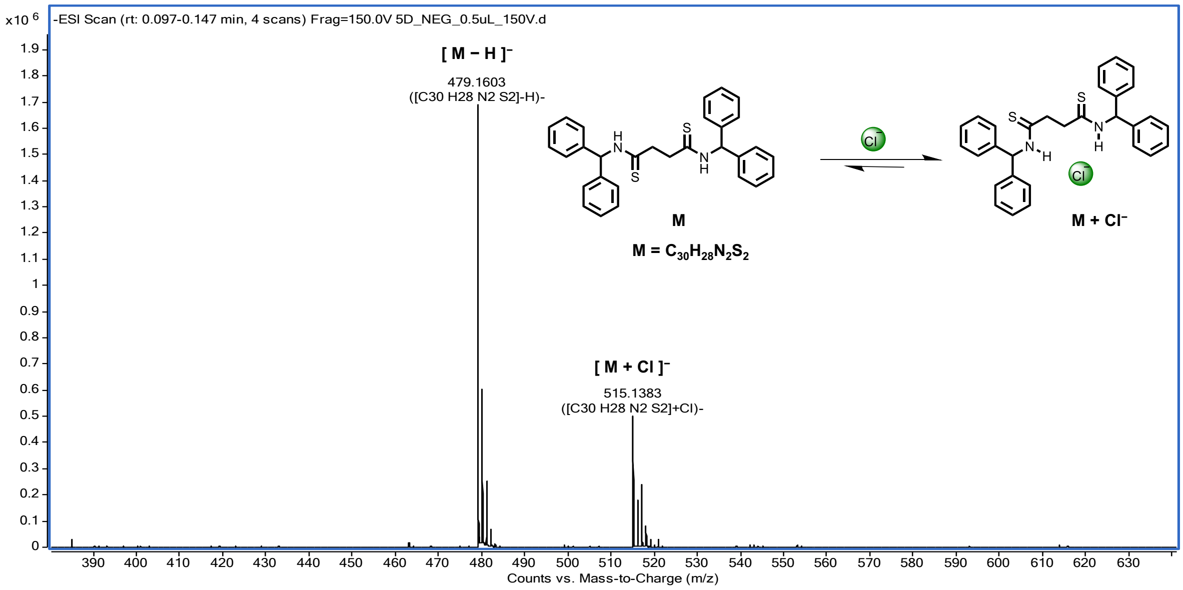

Figure S36. Negative mode ESI-MS spectrum of mixture of **9** and TBACl in acetonitrile.

S8 Crystal structure details

S8.1. Crystal structure of receptor 3

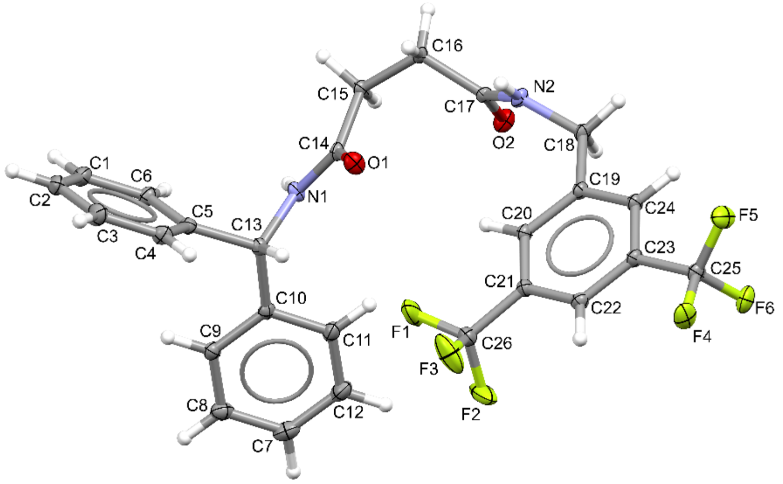

Figure S37. Single crystal structure of receptor **3**.

Computing details

Data collection: Bruker *APEX3*; cell refinement: Bruker *SAINT*; data reduction: Bruker *SAINT*; program(s) used to solve structure: SHELXT 2014/5 (Sheldrick, 2014); program(s) used to refine structure: *SHELXL2017*/1 (Sheldrick, 2017).

Crystal data

$C_{26}H_{22}F_6N_2O_2$	Z = 2
M_r = 508.45	F(000) = 524
Triclinic, $P\bar{1}$	D_x = 1.466 Mg m^{-3}
a = 9.5617 (12) Å	Mo $K\alpha$ radiation, λ = 0.71073 Å
b = 10.8330 (13) Å	Cell parameters from 9599 reflections
c = 11.9272 (14) Å	θ = 2.4–40.4°
α = 96.483 (5)°	μ = 0.13 mm^{-1}
β = 106.465 (5)°	T = 100 K
γ = 99.458 (5)°	Rectangle, colourless
V = 1151.9 (2) Å^3	0.69 × 0.29 × 0.18 mm

Data collection

Bruker Kappa APEX-II DUO diffractometer	R_{int} = 0.136
Radiation source: fine-focus sealed tube	θ_{max} = 40.5°, θ_{min} = 1.8°
φ and ω scans	h = -17→17
115043 measured reflections	k = -19→19
14688 independent reflections	l = -21→21
9942 reflections with $I > 2\sigma(I)$	

Refinement

Refinement on F^2	0 restraints
Least-squares matrix: full	Hydrogen site location: mixed
$R[F^2 > 2\sigma(F^2)]$ = 0.058	H atoms treated by a mixture of independent and constrained refinement
$wR(F^2)$ = 0.175	$w = 1/[\sigma^2(F_o^2) + (0.0922P)^2 + 0.2194P]$ where $P = (F_o^2 + 2F_c^2)/3$
S = 1.03	$(\Delta/\sigma)_{max}$ < 0.001
14688 reflections	$\Delta\rho_{max}$ = 0.92 e Å^{-3}
331 parameters	$\Delta\rho_{min}$ = -0.51 e Å^{-3}

Special details

Geometry. All e.s.d.'s (except the e.s.d. in the dihedral angle between two l.s. planes) are estimated using the full covariance matrix. The cell e.s.d.'s are taken into account individually in the estimation of e.s.d.'s in distances, angles and torsion angles; correlations between e.s.d.'s in cell parameters are only used when they are defined by crystal symmetry. An approximate (isotropic) treatment of cell e.s.d.'s is used for estimating e.s.d.'s involving l.s. planes.

Fractional atomic coordinates and isotropic or equivalent isotropic displacement parameters (Å^2)

	x	*y*	*z*	U_{iso}*/U_{eq}
C1	0.82010 (12)	0.84945 (9)	0.12801 (9)	0.01808 (16)
H1	0.904963	0.914202	0.138315	0.022*
C2	0.67882 (13)	0.86650 (10)	0.06761 (9)	0.01970 (17)
H2	0.667049	0.942946	0.037497	0.024*
C3	0.55527 (12)	0.77110 (10)	0.05167 (9)	0.02094 (18)
H3	0.458651	0.781845	0.009952	0.025*
C4	0.57305 (11)	0.65935 (9)	0.09698 (9)	0.01684 (15)
H4	0.488075	0.594475	0.086025	0.020*
C5	0.71367 (10)	0.64196 (8)	0.15793 (7)	0.01242 (13)
C6	0.83785 (10)	0.73821 (9)	0.17337 (8)	0.01536 (14)
H6	0.934586	0.727603	0.214942	0.018*
C7	0.89125 (12)	0.24100 (10)	0.01966 (10)	0.02216 (19)
H7	0.925550	0.178000	-0.021417	0.027*
C8	0.86894 (11)	0.35122 (10)	-0.02569 (9)	0.01898 (17)
H8	0.887521	0.363602	-0.098185	0.023*
C9	0.81916 (10)	0.44396 (9)	0.03529 (8)	0.01473 (14)
H9	0.804444	0.519368	0.004075	0.018*
C10	0.79095 (9)	0.42685 (8)	0.14130 (8)	0.01236 (13)
C11	0.81326 (11)	0.31523 (9)	0.18590 (9)	0.01751 (16)
H11	0.794136	0.302240	0.258072	0.021*
C12	0.86312 (12)	0.22332 (10)	0.12554 (10)	0.02168 (18)
H12	0.878113	0.147933	0.156711	0.026*
C13	0.72909 (9)	0.52107 (8)	0.20859 (7)	0.01182 (13)
H13	0.626691	0.477583	0.204629	0.014*
C14	0.75307 (9)	0.55007 (8)	0.42047 (7)	0.01123 (12)
Continued…..				

Continued…..				
C15	0.85551 (10)	0.60722 (8)	0.54424 (8)	0.01373 (14)
H15A	0.880447	0.700645	0.550588	0.016*
H15B	0.949427	0.575746	0.557369	0.016*
C16	0.78631 (10)	0.57491 (8)	0.64057 (8)	0.01351 (14)
H16A	0.849107	0.627389	0.717292	0.016*
H16B	0.686786	0.597102	0.621939	0.016*
C17	0.77009 (9)	0.43671 (8)	0.65319 (7)	0.01133 (13)
C18	0.60501 (10)	0.25233 (8)	0.68090 (8)	0.01418 (14)
H18A	0.583346	0.249397	0.757066	0.017*
H18B	0.693246	0.214257	0.684962	0.017*
C19	0.47371 (9)	0.17416 (8)	0.58183 (8)	0.01221 (13)
C20	0.45608 (10)	0.18816 (8)	0.46387 (8)	0.01322 (14)
H20	0.524086	0.250927	0.445478	0.016*
C21	0.33905 (10)	0.11020 (8)	0.37369 (8)	0.01359 (14)
C22	0.23734 (10)	0.01781 (8)	0.39875 (8)	0.01420 (14)
H22	0.157709	-0.035580	0.336574	0.017*
C23	0.25473 (10)	0.00551 (8)	0.51586 (8)	0.01332 (13)
C24	0.37156 (10)	0.08330 (8)	0.60755 (8)	0.01337 (14)
H24	0.381483	0.074315	0.687590	0.016*
C25	0.15285 (11)	-0.09710 (9)	0.54612 (9)	0.01660 (15)
C26	0.32463 (12)	0.11873 (10)	0.24653 (8)	0.01813 (16)
N1	0.81749 (8)	0.55057 (8)	0.33396 (7)	0.01290 (12)
H1N	0.9127 (17)	0.5741 (15)	0.3510 (14)	0.015*
N2	0.63989 (8)	0.38338 (7)	0.66624 (7)	0.01312 (12)
H2N	0.5698 (18)	0.4244 (15)	0.6534 (14)	0.016*
O1	0.61860 (7)	0.50861 (7)	0.40184 (6)	0.01664 (12)
O2	0.87230 (8)	0.37906 (7)	0.65612 (7)	0.01768 (13)
Continued…..				

Continued…..				
F1	0.40482 (12)	0.22417 (9)	0.23246 (7)	0.0411 (2)
F2	0.36550 (14)	0.02134 (9)	0.19445 (7)	0.0440 (3)
F3	0.18424 (10)	0.11483 (12)	0.18280 (8)	0.0440 (2)
F4	0.02699 (8)	-0.14398 (7)	0.45728 (6)	0.02471 (14)
F5	0.11317 (8)	-0.05710 (7)	0.64095 (7)	0.02608 (15)
F6	0.21837 (8)	-0.19500 (6)	0.57291 (7)	0.02423 (14)

Atomic displacement parameters (Å^2)

	U^{11}	U^{22}	U^{33}	U^{12}	U^{13}	U^{23}
C1	0.0228 (4)	0.0122 (3)	0.0182 (4)	0.0010 (3)	0.0064 (3)	0.0020 (3)
C2	0.0278 (5)	0.0144 (4)	0.0155 (4)	0.0050 (3)	0.0040 (3)	0.0031 (3)
C3	0.0212 (4)	0.0188 (4)	0.0195 (4)	0.0062 (3)	-0.0007 (3)	0.0042 (3)
C4	0.0146 (3)	0.0164 (4)	0.0160 (4)	0.0019 (3)	0.0003 (3)	0.0018 (3)
C5	0.0127 (3)	0.0126 (3)	0.0109 (3)	0.0016 (2)	0.0028 (2)	0.0011 (2)
C6	0.0149 (3)	0.0130 (3)	0.0171 (4)	0.0011 (3)	0.0043 (3)	0.0023 (3)
C7	0.0208 (4)	0.0162 (4)	0.0260 (5)	0.0029 (3)	0.0050 (4)	-0.0038 (3)
C8	0.0189 (4)	0.0169 (4)	0.0194 (4)	-0.0005 (3)	0.0073 (3)	-0.0021 (3)
C9	0.0155 (3)	0.0130 (3)	0.0146 (3)	-0.0005 (3)	0.0053 (3)	0.0015 (3)
C10	0.0111 (3)	0.0109 (3)	0.0128 (3)	-0.0006 (2)	0.0018 (2)	0.0017 (2)
C11	0.0203 (4)	0.0126 (3)	0.0167 (4)	0.0010 (3)	0.0019 (3)	0.0038 (3)
C12	0.0241 (5)	0.0122 (4)	0.0244 (4)	0.0033 (3)	0.0014 (4)	0.0018 (3)
C13	0.0097 (3)	0.0132 (3)	0.0113 (3)	-0.0001 (2)	0.0025 (2)	0.0027 (2)
C14	0.0104 (3)	0.0107 (3)	0.0128 (3)	0.0008 (2)	0.0041 (2)	0.0037 (2)
C15	0.0140 (3)	0.0129 (3)	0.0123 (3)	-0.0025 (3)	0.0042 (3)	0.0017 (2)
C16	0.0162 (3)	0.0105 (3)	0.0133 (3)	-0.0014 (3)	0.0065 (3)	0.0009 (2)
C17	0.0102 (3)	0.0114 (3)	0.0108 (3)	-0.0010 (2)	0.0030 (2)	0.0008 (2)
C18	0.0134 (3)	0.0120 (3)	0.0145 (3)	-0.0024 (3)	0.0026 (3)	0.0035 (3)
Continued…..						

Continued…..						
C19	0.0113 (3)	0.0109 (3)	0.0134 (3)	-0.0007 (2)	0.0039 (2)	0.0022 (2)
C20	0.0140 (3)	0.0117 (3)	0.0140 (3)	-0.0006 (2)	0.0059 (3)	0.0029 (3)
C21	0.0155 (3)	0.0129 (3)	0.0120 (3)	0.0010 (3)	0.0047 (3)	0.0021 (2)
C22	0.0143 (3)	0.0124 (3)	0.0140 (3)	-0.0008 (3)	0.0040 (3)	0.0006 (3)
C23	0.0126 (3)	0.0116 (3)	0.0147 (3)	-0.0018 (2)	0.0049 (3)	0.0018 (3)
C24	0.0136 (3)	0.0122 (3)	0.0131 (3)	-0.0017 (2)	0.0045 (3)	0.0026 (2)
C25	0.0159 (4)	0.0140 (3)	0.0179 (4)	-0.0033 (3)	0.0059 (3)	0.0021 (3)
C26	0.0229 (4)	0.0171 (4)	0.0135 (3)	0.0017 (3)	0.0053 (3)	0.0027 (3)
N1	0.0088 (3)	0.0179 (3)	0.0109 (3)	-0.0008 (2)	0.0031 (2)	0.0031 (2)
N2	0.0103 (3)	0.0106 (3)	0.0180 (3)	-0.0008 (2)	0.0053 (2)	0.0025 (2)
O1	0.0091 (2)	0.0229 (3)	0.0172 (3)	-0.0003 (2)	0.0047 (2)	0.0037 (2)
O2	0.0118 (3)	0.0164 (3)	0.0258 (3)	0.0025 (2)	0.0071 (2)	0.0048 (2)
F1	0.0640 (6)	0.0336 (4)	0.0186 (3)	-0.0169 (4)	0.0164 (4)	0.0051 (3)
F2	0.0861 (7)	0.0398 (5)	0.0179 (3)	0.0363 (5)	0.0208 (4)	0.0059 (3)
F3	0.0295 (4)	0.0799 (8)	0.0233 (4)	0.0129 (4)	0.0024 (3)	0.0245 (4)
F4	0.0179 (3)	0.0232 (3)	0.0247 (3)	-0.0103 (2)	0.0015 (2)	0.0045 (2)
F5	0.0298 (3)	0.0232 (3)	0.0265 (3)	-0.0059 (3)	0.0186 (3)	0.0002 (3)
F6	0.0271 (3)	0.0162 (3)	0.0311 (3)	0.0010 (2)	0.0110 (3)	0.0104 (2)

Geometric parameters (Å, º)

C1—C6	1.3914 (13)	C15—H15A	0.9900
C1—C2	1.3922 (15)	C15—H15B	0.9900
C1—H1	0.9500	C16—C17	1.5086 (12)
C2—C3	1.3881 (16)	C16—H16A	0.9900
C2—H2	0.9500	C16—H16B	0.9900
C3—C4	1.3964 (14)	C17—O2	1.2380 (11)
C3—H3	0.9500	C17—N2	1.3422 (11)
Continued…..			

Continued…..			
C4—C5	1.3899 (13)	C18—N2	1.4459 (12)
C4—H4	0.9500	C18—C19	1.5078 (12)
C5—C6	1.3989 (13)	C18—H18A	0.9900
C5—C13	1.5144 (12)	C18—H18B	0.9900
C6—H6	0.9500	C19—C24	1.3924 (12)
C7—C8	1.3886 (16)	C19—C20	1.3975 (12)
C7—C12	1.3907 (17)	C20—C21	1.3872 (13)
C7—H7	0.9500	C20—H20	0.9500
C8—C9	1.3977 (13)	C21—C22	1.3962 (13)
C8—H8	0.9500	C21—C26	1.4984 (13)
C9—C10	1.3912 (13)	C22—C23	1.3836 (13)
C9—H9	0.9500	C22—H22	0.9500
C10—C11	1.3987 (13)	C23—C24	1.3943 (12)
C10—C13	1.5225 (12)	C23—C25	1.4997 (12)
C11—C12	1.3876 (15)	C24—H24	0.9500
C11—H11	0.9500	C25—F4	1.3356 (12)
C12—H12	0.9500	C25—F5	1.3408 (12)
C13—N1	1.4626 (11)	C25—F6	1.3419 (12)
C13—H13	1.0000	C26—F1	1.3226 (13)
C14—O1	1.2370 (10)	C26—F2	1.3330 (13)
C14—N1	1.3433 (11)	C26—F3	1.3331 (13)
C14—C15	1.5155 (12)	N1—H1N	0.862 (16)
C15—C16	1.5251 (12)	N2—H2N	0.851 (16)
C6—C1—C2	120.45 (9)	C17—C16—C15	113.21 (7)
C6—C1—H1	119.8	C17—C16—H16A	108.9
C2—C1—H1	119.8	C15—C16—H16A	108.9
Continued…..			

Continued…..			
C3—C2—C1	119.59 (9)	C17—C16—H16B	108.9
C3—C2—H2	120.2	C15—C16—H16B	108.9
C1—C2—H2	120.2	H16A—C16—H16B	107.7
C2—C3—C4	119.98 (9)	O2—C17—N2	122.92 (8)
C2—C3—H3	120.0	O2—C17—C16	121.99 (8)
C4—C3—H3	120.0	N2—C17—C16	115.02 (7)
C5—C4—C3	120.74 (9)	N2—C18—C19	113.03 (7)
C5—C4—H4	119.6	N2—C18—H18A	109.0
C3—C4—H4	119.6	C19—C18—H18A	109.0
C4—C5—C6	119.04 (8)	N2—C18—H18B	109.0
C4—C5—C13	119.54 (8)	C19—C18—H18B	109.0
C6—C5—C13	121.41 (8)	H18A—C18—H18B	107.8
C1—C6—C5	120.20 (9)	C24—C19—C20	119.41 (8)
C1—C6—H6	119.9	C24—C19—C18	119.64 (8)
C5—C6—H6	119.9	C20—C19—C18	120.90 (8)
C8—C7—C12	119.65 (9)	C21—C20—C19	119.85 (8)
C8—C7—H7	120.2	C21—C20—H20	120.1
C12—C7—H7	120.2	C19—C20—H20	120.1
C7—C8—C9	120.01 (10)	C20—C21—C22	121.03 (8)
C7—C8—H8	120.0	C20—C21—C26	120.31 (8)
C9—C8—H8	120.0	C22—C21—C26	118.57 (8)
C10—C9—C8	120.60 (9)	C23—C22—C21	118.73 (8)
C10—C9—H9	119.7	C23—C22—H22	120.6
C8—C9—H9	119.7	C21—C22—H22	120.6
C9—C10—C11	118.88 (8)	C22—C23—C24	120.96 (8)
C9—C10—C13	123.10 (8)	C22—C23—C25	120.36 (8)
C11—C10—C13	117.94 (8)	C24—C23—C25	118.60 (8)
Continued…..			

Continued…..			
C12—C11—C10	120.52 (9)	C19—C24—C23	120.01 (8)
C12—C11—H11	119.7	C19—C24—H24	120.0
C10—C11—H11	119.7	C23—C24—H24	120.0
C11—C12—C7	120.34 (10)	F4—C25—F5	106.86 (8)
C11—C12—H12	119.8	F4—C25—F6	106.96 (8)
C7—C12—H12	119.8	F5—C25—F6	105.91 (8)
N1—C13—C5	110.50 (7)	F4—C25—C23	112.66 (8)
N1—C13—C10	110.31 (7)	F5—C25—C23	112.57 (8)
C5—C13—C10	114.99 (7)	F6—C25—C23	111.45 (8)
N1—C13—H13	106.9	F1—C26—F2	107.43 (10)
C5—C13—H13	106.9	F1—C26—F3	106.28 (10)
C10—C13—H13	106.9	F2—C26—F3	105.79 (10)
O1—C14—N1	123.12 (8)	F1—C26—C21	113.46 (8)
O1—C14—C15	121.25 (8)	F2—C26—C21	111.45 (8)
N1—C14—C15	115.62 (7)	F3—C26—C21	111.97 (9)
C14—C15—C16	112.67 (7)	C14—N1—C13	121.66 (7)
C14—C15—H15A	109.1	C14—N1—H1N	120.4 (10)
C16—C15—H15A	109.1	C13—N1—H1N	117.8 (10)
C14—C15—H15B	109.1	C17—N2—C18	122.87 (8)
C16—C15—H15B	109.1	C17—N2—H2N	118.2 (11)
H15A—C15—H15B	107.8	C18—N2—H2N	118.1 (11)
C6—C1—C2—C3	-0.66 (15)	C24—C19—C20—C21	-1.16 (13)
C1—C2—C3—C4	0.54 (16)	C18—C19—C20—C21	176.36 (8)
C2—C3—C4—C5	-0.19 (16)	C19—C20—C21—C22	0.35 (13)
C3—C4—C5—C6	-0.04 (14)	C19—C20—C21—C26	-176.11 (8)
C3—C4—C5—C13	178.55 (9)	C20—C21—C22—C23	0.32 (14)
Continued			

Continued…..			
C2—C1—C6—C5	0.44 (15)	C26—C21—C22—C23	176.84 (8)
C4—C5—C6—C1	-0.08 (14)	C21—C22—C23—C24	-0.18 (14)
C13—C5—C6—C1	-178.65 (8)	C21—C22—C23—C25	-176.70 (8)
C12—C7—C8—C9	0.31 (16)	C20—C19—C24—C23	1.30 (13)
C7—C8—C9—C10	-0.26 (15)	C18—C19—C24—C23	-176.25 (8)
C8—C9—C10—C11	0.01 (13)	C22—C23—C24—C19	-0.64 (14)
C8—C9—C10—C13	-176.78 (8)	C25—C23—C24—C19	175.94 (8)
C9—C10—C11—C12	0.19 (14)	C22—C23—C25—F4	-18.63 (13)
C13—C10—C11—C12	177.14 (9)	C24—C23—C25—F4	164.77 (8)
C10—C11—C12—C7	-0.14 (15)	C22—C23—C25—F5	-139.55 (9)
C8—C7—C12—C11	-0.11 (16)	C24—C23—C25—F5	43.85 (12)
C4—C5—C13—N1	-128.41 (9)	C22—C23—C25—F6	101.62 (10)
C6—C5—C13—N1	50.15 (11)	C24—C23—C25—F6	-74.98 (11)
C4—C5—C13—C10	105.92 (9)	C20—C21—C26—F1	-16.78 (14)
C6—C5—C13—C10	-75.52 (10)	C22—C21—C26—F1	166.67 (10)
C9—C10—C13—N1	-131.28 (9)	C20—C21—C26—F2	104.64 (11)
C11—C10—C13—N1	51.91 (10)	C22—C21—C26—F2	-71.91 (12)
C9—C10—C13—C5	-5.51 (12)	C20—C21—C26—F3	-137.08 (10)
C11—C10—C13—C5	177.67 (8)	C22—C21—C26—F3	46.37 (13)
O1—C14—C15—C16	15.57 (12)	O1—C14—N1—C13	10.17 (13)
N1—C14—C15—C16	-165.88 (8)	C15—C14—N1—C13	-168.35 (8)
C14—C15—C16—C17	69.32 (10)	C5—C13—N1—C14	98.82 (9)
C15—C16—C17—O2	45.94 (12)	C10—C13—N1—C14	-132.92 (8)
C15—C16—C17—N2	-136.90 (8)	O2—C17—N2—C18	-2.16 (14)
N2—C18—C19—C24	-140.99 (8)	C16—C17—N2—C18	-179.29 (8)
N2—C18—C19—C20	41.49 (12)	C19—C18—N2—C17	-117.51 (9)

Hydrogen-bond geometry (Å, º)

D—H···*A*	*D*—H	H···*A*	*D*···*A*	*D*—H···*A*
C15—H15*A*···F4^{i}	0.99	2.56	3.3424 (11)	136
N1—H1*N*···O2^{ii}	0.862 (16)	2.063 (16)	2.9036 (11)	164.9 (15)
N2—H2*N*···O1^{iii}	0.851 (16)	2.018 (16)	2.8573 (11)	168.6 (15)

Symmetry codes: (i) *x*+1, *y*+1, *z*; (ii) -*x*+2, -*y*+1, -*z*+1; (iii) -*x*+1, -*y*+1, -*z*+1.
Document origin: *publCIF* [Westrip, S. P. (2010). *J. Apply. Cryst.*, **43**, 920-925].

S8.2. Crystal structure of receptor 4

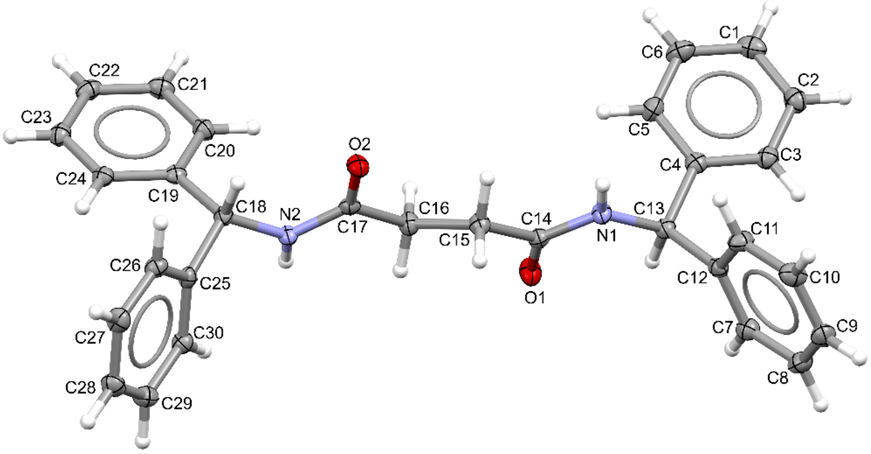

Figure S38. Crystal structure of receptor **4**.

Computing details

Data collection: Bruker *APEX3*; cell refinement: Bruker *SAINT*; data reduction: Bruker *SAINT*; program(s) used to solve structure: SHELXT 2014/5 (Sheldrick, 2014); program(s) used to refine structure: *SHELXL2017*/1 (Sheldrick, 2017).

Crystal data

$C_{30}H_{28}Cl_0N_2O_2$	*F*(000) = 952
M_r = 448.54	D_x = 1.254 Mg m^{-3}
Monoclinic, $P2_1/n$	Cu *Kα* radiation, l = 1.54184 Å
a = 9.5813 (14) Å	Cell parameters from 9949 reflections
b = 27.725 (4) Å	*θ* = 3.2–68.6°

c = 9.8074 (15) Å	μ = 0.62 mm^{-1}
β = 114.246 (6)°	T = 100 K
V = 2375.4 (6) $Å^3$	Rectangle, colourless
Z = 4	0.29 × 0.12 × 0.04 mm

Data collection

Bruker Kappa APEX-II DUO diffractometer	4339 independent reflections
Radiation source: fine-focus sealed tube	3960 reflections with $I > 2\sigma(I)$
TRIUMPH curved graphite monochromator	R_{int} = 0.037
φ and ω scans	θ_{max} = 68.7°, θ_{min} = 3.2°
Absorption correction: multi-scan *SADABS* (Krause *et al.*, 2015)	h = -10→11
T_{min} = 0.769, T_{max} = 0.929	k = -33→33
21807 measured reflections	l = -11→11

Refinement

Refinement on F^2	0 restraints
Least-squares matrix: full	Hydrogen site location: mixed
$R[F^2 > 2\sigma(F^2)]$ = 0.038	H atoms treated by a mixture of independent and constrained refinement
$wR(F^2)$ = 0.109	$w = 1/[\sigma^2(F_o^2) + (0.0558P)^2 + 0.8371P]$ where $P = (F_o^2 + 2F_c^2)/3$
S = 1.09	$(\Delta/\sigma)_{max}$ = 0.001
4339 reflections	$\Delta\rho_{max}$ = 0.34 e $Å^{-3}$
313 parameters	$\Delta\rho_{min}$ = -0.27 e $Å^{-3}$

Special details

Geometry. All e.s.d.'s (except the e.s.d. in the dihedral angle between two l.s. planes) are estimated using the full covariance matrix. The cell e.s.d.'s are taken into account individually in the estimation of e.s.d.'s in distances, angles and torsion angles; correlations between e.s.d.'s in cell parameters are only used when they are defined by crystal symmetry. An

approximate (isotropic) treatment of cell e.s.d.'s is used for estimating e.s.d.'s involving l.s. planes.

Fractional atomic coordinates and isotropic or equivalent isotropic displacement parameters (Å^2)

	x	*y*	*z*	U_{iso}*/U_{eq}
C1	0.65480 (16)	0.24366 (5)	0.17902 (14)	0.0238 (3)
H1	0.614173	0.212831	0.140858	0.029*
C2	0.81063 (16)	0.25018 (5)	0.25166 (15)	0.0226 (3)
H2	0.877807	0.223779	0.263299	0.027*
C3	0.86992 (15)	0.29518 (5)	0.30794 (14)	0.0194 (3)
H3	0.977587	0.299269	0.358291	0.023*
C4	0.77372 (14)	0.33434 (4)	0.29156 (13)	0.0167 (3)
C5	0.61638 (15)	0.32738 (5)	0.21786 (14)	0.0216 (3)
H5	0.548714	0.353669	0.205771	0.026*
C6	0.55775 (16)	0.28238 (5)	0.16199 (15)	0.0254 (3)
H6	0.450182	0.278051	0.111644	0.030*
C7	1.11993 (15)	0.38584 (4)	0.52896 (14)	0.0199 (3)
H7	1.094419	0.381363	0.612042	0.024*
C8	1.27259 (15)	0.38824 (5)	0.55129 (15)	0.0232 (3)
H8	1.350828	0.384894	0.649289	0.028*
C9	1.31139 (15)	0.39547 (5)	0.43159 (16)	0.0236 (3)
H9	1.415900	0.396855	0.447049	0.028*
C10	1.19716 (16)	0.40067 (5)	0.28947 (15)	0.0244 (3)
H10	1.222959	0.406068	0.206969	0.029*
C11	1.04462 (15)	0.39800 (5)	0.26731 (14)	0.0217 (3)
H11	0.966584	0.401756	0.169378	0.026*
C12	1.00456 (14)	0.38991 (4)	0.38644 (13)	0.0163 (3)
C13	0.83893 (14)	0.38299 (4)	0.36255 (13)	0.0163 (3)
H13	0.837582	0.383094	0.463964	0.020*
C14	0.66647 (14)	0.45080 (4)	0.33867 (13)	0.0164 (3)
C15	0.58686 (15)	0.49406 (4)	0.24576 (13)	0.0176 (3)
Continued…..				

Continued…..				
H15A	0.550294	0.485757	0.138476	0.021*
H15B	0.661245	0.520820	0.266847	0.021*
C16	0.45229 (14)	0.51081 (4)	0.27708 (14)	0.0176 (3)
H16A	0.380529	0.483547	0.261097	0.021*
H16B	0.489669	0.520723	0.383159	0.021*
C17	0.36749 (14)	0.55257 (4)	0.17837 (13)	0.0149 (3)
C18	0.19710 (13)	0.62081 (4)	0.15359 (13)	0.0148 (3)
H18	0.198430	0.620068	0.052189	0.018*
C19	0.03230 (14)	0.60936 (4)	0.13035 (13)	0.0158 (3)
C20	-0.01810 (15)	0.56165 (4)	0.11503 (14)	0.0197 (3)
H20	0.052259	0.536236	0.126132	0.024*
C21	-0.16938 (15)	0.55080 (5)	0.08385 (14)	0.0219 (3)
H21	-0.201636	0.518100	0.074383	0.026*
C22	-0.27392 (15)	0.58744 (5)	0.06640 (14)	0.0205 (3)
H22	-0.377928	0.580050	0.043722	0.025*
C23	-0.22490 (14)	0.63486 (5)	0.08242 (13)	0.0194 (3)
H23	-0.295681	0.660154	0.071161	0.023*
C24	-0.07324 (14)	0.64579 (4)	0.11481 (13)	0.0172 (3)
H24	-0.041019	0.678516	0.126507	0.021*
C25	0.24908 (13)	0.67092 (4)	0.21548 (13)	0.0156 (3)
C26	0.22720 (14)	0.70862 (5)	0.11515 (14)	0.0187 (3)
H26	0.187615	0.702087	0.010960	0.022*
C27	0.26259 (15)	0.75557 (5)	0.16573 (15)	0.0213 (3)
H27	0.244752	0.781166	0.096146	0.026*
C28	0.32378 (15)	0.76524 (5)	0.31717 (15)	0.0222 (3)
H28	0.348974	0.797402	0.352003	0.027*
C29	0.34833 (15)	0.72779 (5)	0.41831 (14)	0.0222 (3)
H29	0.391183	0.734293	0.522612	0.027*
C30	0.31044 (14)	0.68092 (4)	0.36742 (14)	0.0183 (3)
H30	0.326620	0.655454	0.437112	0.022*
Continued…..				

Continued…..				
N1	0.74273 (12)	0.42302 (4)	0.28014 (12)	0.0166 (2)
H1N	0.7288 (17)	0.4288 (5)	0.1868 (18)	0.020*
N2	0.30102 (12)	0.58298 (4)	0.24027 (11)	0.0153 (2)
H2N	0.3070 (17)	0.5756 (5)	0.3317 (18)	0.018*
O1	0.66426 (11)	0.44278 (3)	0.46155 (10)	0.0236 (2)
O2	0.35740 (10)	0.55702 (3)	0.04918 (9)	0.0194 (2)

Atomic displacement parameters (Å^2)

	U^{11}	U^{22}	U^{33}	U^{12}	U^{13}	U^{23}
C1	0.0330 (8)	0.0192 (6)	0.0182 (6)	-0.0058 (5)	0.0094 (6)	0.0001 (5)
C2	0.0280 (7)	0.0177 (6)	0.0228 (6)	0.0029 (5)	0.0111 (6)	0.0020 (5)
C3	0.0198 (6)	0.0202 (6)	0.0175 (6)	0.0014 (5)	0.0070 (5)	0.0022 (5)
C4	0.0195 (6)	0.0179 (6)	0.0138 (6)	0.0002 (5)	0.0078 (5)	0.0024 (5)
C5	0.0183 (6)	0.0224 (7)	0.0224 (6)	0.0026 (5)	0.0066 (5)	0.0028 (5)
C6	0.0215 (7)	0.0278 (7)	0.0230 (7)	-0.0056 (6)	0.0051 (6)	0.0017 (5)
C7	0.0223 (7)	0.0190 (6)	0.0181 (6)	-0.0004 (5)	0.0081 (5)	0.0010 (5)
C8	0.0191 (7)	0.0243 (7)	0.0208 (6)	-0.0015 (5)	0.0027 (5)	0.0007 (5)
C9	0.0184 (6)	0.0229 (7)	0.0289 (7)	-0.0036 (5)	0.0091 (6)	-0.0029 (5)
C10	0.0252 (7)	0.0292 (7)	0.0218 (7)	-0.0061 (6)	0.0128 (6)	-0.0034 (5)
C11	0.0198 (7)	0.0285 (7)	0.0155 (6)	-0.0027 (5)	0.0060 (5)	-0.0015 (5)
C12	0.0190 (6)	0.0133 (6)	0.0163 (6)	0.0002 (5)	0.0068 (5)	-0.0011 (4)
C13	0.0179 (6)	0.0164 (6)	0.0141 (6)	0.0020 (5)	0.0062 (5)	0.0018 (4)
C14	0.0162 (6)	0.0172 (6)	0.0150 (6)	-0.0002 (5)	0.0057 (5)	0.0003 (5)
C15	0.0213 (6)	0.0173 (6)	0.0154 (6)	0.0024 (5)	0.0089 (5)	0.0023 (5)
C16	0.0196 (6)	0.0173 (6)	0.0177 (6)	0.0027 (5)	0.0095 (5)	0.0024 (5)
C17	0.0144 (6)	0.0147 (6)	0.0147 (6)	-0.0028 (5)	0.0051 (5)	-0.0010 (4)
C18	0.0154 (6)	0.0155 (6)	0.0129 (6)	0.0020 (5)	0.0052 (5)	0.0015 (4)
C19	0.0175 (6)	0.0183 (6)	0.0113 (5)	-0.0006 (5)	0.0056 (5)	0.0007 (4)
C20	0.0222 (7)	0.0166 (6)	0.0201 (6)	0.0005 (5)	0.0087 (5)	-0.0008 (5)
C21	0.0247 (7)	0.0206 (6)	0.0209 (7)	-0.0058 (5)	0.0098 (6)	-0.0018 (5)
Continued…..						

Continued…..						
C22	0.0173 (6)	0.0289 (7)	0.0150 (6)	-0.0037 (5)	0.0064 (5)	-0.0001 (5)
C23	0.0184 (6)	0.0240 (6)	0.0158 (6)	0.0036 (5)	0.0070 (5)	0.0022 (5)
C24	0.0192 (6)	0.0163 (6)	0.0147 (6)	0.0000 (5)	0.0055 (5)	0.0013 (4)
C25	0.0130 (6)	0.0168 (6)	0.0177 (6)	0.0005 (5)	0.0070 (5)	0.0005 (5)
C26	0.0181 (6)	0.0206 (6)	0.0166 (6)	-0.0011 (5)	0.0064 (5)	0.0011 (5)
C27	0.0224 (7)	0.0176 (6)	0.0240 (7)	-0.0012 (5)	0.0096 (5)	0.0034 (5)
C28	0.0232 (7)	0.0176 (6)	0.0261 (7)	-0.0038 (5)	0.0107 (6)	-0.0033 (5)
C29	0.0235 (7)	0.0242 (7)	0.0182 (6)	-0.0029 (5)	0.0077 (5)	-0.0035 (5)
C30	0.0186 (6)	0.0184 (6)	0.0176 (6)	0.0003 (5)	0.0071 (5)	0.0019 (5)
N1	0.0204 (5)	0.0170 (5)	0.0130 (5)	0.0042 (4)	0.0075 (4)	0.0033 (4)
N2	0.0179 (5)	0.0162 (5)	0.0120 (5)	0.0030 (4)	0.0062 (4)	0.0019 (4)
O1	0.0296 (5)	0.0277 (5)	0.0178 (5)	0.0103 (4)	0.0140 (4)	0.0063 (4)
O2	0.0243 (5)	0.0207 (5)	0.0148 (4)	0.0040 (4)	0.0095 (4)	0.0019 (3)

Geometric parameters (Å, º)

C1—C2	1.377 (2)	C16—H16A	0.9900
C1—C6	1.385 (2)	C16—H16B	0.9900
C1—H1	0.9500	C17—O2	1.2366 (15)
C2—C3	1.3880 (18)	C17—N2	1.3425 (16)
C2—H2	0.9500	C18—N2	1.4558 (15)
C3—C4	1.3902 (18)	C18—C25	1.5160 (16)
C3—H3	0.9500	C18—C19	1.5331 (17)
C4—C5	1.3919 (18)	C18—H18	1.0000
C4—C13	1.5288 (17)	C19—C24	1.3926 (18)
C5—C6	1.3855 (19)	C19—C20	1.3950 (17)
C5—H5	0.9500	C20—C21	1.3860 (19)
C6—H6	0.9500	C20—H20	0.9500
C7—C12	1.3854 (18)	C21—C22	1.3868 (19)
C7—C8	1.3889 (19)	C21—H21	0.9500
C7—H7	0.9500	C22—C23	1.3832 (19)
Continued…..			

Continued…..			
C8—C9	1.384 (2)	C22—H22	0.9500
C8—H8	0.9500	C23—C24	1.3876 (18)
C9—C10	1.381 (2)	C23—H23	0.9500
C9—H9	0.9500	C24—H24	0.9500
C10—C11	1.3885 (19)	C25—C30	1.3869 (17)
C10—H10	0.9500	C25—C26	1.3911 (17)
C11—C12	1.3896 (18)	C26—C27	1.3848 (18)
C11—H11	0.9500	C26—H26	0.9500
C12—C13	1.5181 (17)	C27—C28	1.3807 (19)
C13—N1	1.4571 (15)	C27—H27	0.9500
C13—H13	1.0000	C28—C29	1.3876 (19)
C14—O1	1.2342 (16)	C28—H28	0.9500
C14—N1	1.3420 (16)	C29—C30	1.3860 (18)
C14—C15	1.5098 (17)	C29—H29	0.9500
C15—C16	1.5159 (17)	C30—H30	0.9500
C15—H15A	0.9900	N1—H1N	0.883 (16)
C15—H15B	0.9900	N2—H2N	0.898 (16)
C16—C17	1.5139 (16)		
C2—C1—C6	119.51 (12)	C17—C16—H16B	109.1
C2—C1—H1	120.2	C15—C16—H16B	109.1
C6—C1—H1	120.2	H16A—C16—H16B	107.8
C1—C2—C3	120.23 (12)	O2—C17—N2	123.28 (11)
C1—C2—H2	119.9	O2—C17—C16	121.81 (11)
C3—C2—H2	119.9	N2—C17—C16	114.88 (10)
C2—C3—C4	120.83 (12)	N2—C18—C25	113.14 (10)
C2—C3—H3	119.6	N2—C18—C19	110.69 (10)
C4—C3—H3	119.6	C25—C18—C19	113.47 (10)
C3—C4—C5	118.50 (12)	N2—C18—H18	106.3
C3—C4—C13	120.58 (11)	C25—C18—H18	106.3
Continued…..			

Continued…..			
C5—C4—C13	120.78 (11)	C19—C18—H18	106.3
C6—C5—C4	120.46 (12)	C24—C19—C20	118.18 (12)
C6—C5—H5	119.8	C24—C19—C18	121.55 (11)
C4—C5—H5	119.8	C20—C19—C18	120.15 (11)
C1—C6—C5	120.49 (12)	C21—C20—C19	120.93 (12)
C1—C6—H6	119.8	C21—C20—H20	119.5
C5—C6—H6	119.8	C19—C20—H20	119.5
C12—C7—C8	120.49 (12)	C20—C21—C22	120.34 (12)
C12—C7—H7	119.8	C20—C21—H21	119.8
C8—C7—H7	119.8	C22—C21—H21	119.8
C9—C8—C7	120.37 (12)	C23—C22—C21	119.20 (12)
C9—C8—H8	119.8	C23—C22—H22	120.4
C7—C8—H8	119.8	C21—C22—H22	120.4
C10—C9—C8	119.57 (12)	C22—C23—C24	120.56 (12)
C10—C9—H9	120.2	C22—C23—H23	119.7
C8—C9—H9	120.2	C24—C23—H23	119.7
C9—C10—C11	119.95 (13)	C23—C24—C19	120.77 (12)
C9—C10—H10	120.0	C23—C24—H24	119.6
C11—C10—H10	120.0	C19—C24—H24	119.6
C10—C11—C12	120.89 (12)	C30—C25—C26	118.96 (11)
C10—C11—H11	119.6	C30—C25—C18	122.54 (11)
C12—C11—H11	119.6	C26—C25—C18	118.42 (11)
C7—C12—C11	118.69 (12)	C27—C26—C25	120.62 (11)
C7—C12—C13	119.72 (11)	C27—C26—H26	119.7
C11—C12—C13	121.52 (11)	C25—C26—H26	119.7
N1—C13—C12	111.86 (10)	C28—C27—C26	120.07 (12)
N1—C13—C4	111.86 (10)	C28—C27—H27	120.0
C12—C13—C4	112.82 (10)	C26—C27—H27	120.0
N1—C13—H13	106.6	C27—C28—C29	119.75 (12)
C12—C13—H13	106.6	C27—C28—H28	120.1
Continued…..			

Continued…..			
C4—C13—H13	106.6	C29—C28—H28	120.1
O1—C14—N1	123.02 (11)	C30—C29—C28	120.11 (12)
O1—C14—C15	121.70 (11)	C30—C29—H29	119.9
N1—C14—C15	115.25 (10)	C28—C29—H29	119.9
C14—C15—C16	112.46 (10)	C29—C30—C25	120.47 (12)
C14—C15—H15A	109.1	C29—C30—H30	119.8
C16—C15—H15A	109.1	C25—C30—H30	119.8
C14—C15—H15B	109.1	C14—N1—C13	122.10 (10)
C16—C15—H15B	109.1	C14—N1—H1N	117.6 (10)
H15A—C15—H15B	107.8	C13—N1—H1N	120.3 (10)
C17—C16—C15	112.49 (10)	C17—N2—C18	121.74 (10)
C17—C16—H16A	109.1	C17—N2—H2N	117.4 (10)
C15—C16—H16A	109.1	C18—N2—H2N	119.7 (10)
C6—C1—C2—C3	0.3 (2)	N2—C18—C19—C20	-33.53 (15)
C1—C2—C3—C4	-0.29 (19)	C25—C18—C19—C20	-162.00 (11)
C2—C3—C4—C5	0.23 (18)	C24—C19—C20—C21	0.57 (18)
C2—C3—C4—C13	175.99 (11)	C18—C19—C20—C21	-175.58 (11)
C3—C4—C5—C6	-0.17 (19)	C19—C20—C21—C22	0.38 (19)
C13—C4—C5—C6	-175.92 (12)	C20—C21—C22—C23	-0.84 (19)
C2—C1—C6—C5	-0.2 (2)	C21—C22—C23—C24	0.33 (19)
C4—C5—C6—C1	0.2 (2)	C22—C23—C24—C19	0.65 (18)
C12—C7—C8—C9	0.98 (19)	C20—C19—C24—C23	-1.08 (18)
C7—C8—C9—C10	0.5 (2)	C18—C19—C24—C23	175.01 (11)
C8—C9—C10—C11	-0.8 (2)	N2—C18—C25—C30	-41.41 (16)
C9—C10—C11—C12	-0.3 (2)	C19—C18—C25—C30	85.78 (14)
C8—C7—C12—C11	-2.06 (18)	N2—C18—C25—C26	141.81 (11)
C8—C7—C12—C13	174.85 (11)	C19—C18—C25—C26	-91.00 (13)
C10—C11—C12—C7	1.72 (19)	C30—C25—C26—C27	-1.55 (19)
Continued…..			

Continued…..			
C10—C11—C12—C13	-175.13 (12)	C18—C25—C26—C27	175.35 (11)
C7—C12—C13—N1	128.17 (12)	C25—C26—C27—C28	1.6 (2)
C11—C12—C13—N1	-55.01 (15)	C26—C27—C28—C29	-0.6 (2)
C7—C12—C13—C4	-104.67 (13)	C27—C28—C29—C30	-0.5 (2)
C11—C12—C13—C4	72.15 (14)	C28—C29—C30—C25	0.6 (2)
C3—C4—C13—N1	152.39 (11)	C26—C25—C30—C29	0.45 (19)
C5—C4—C13—N1	-31.95 (16)	C18—C25—C30—C29	-176.32 (12)
C3—C4—C13—C12	25.24 (16)	O1—C14—N1—C13	-4.69 (19)
C5—C4—C13—C12	-159.10 (11)	C15—C14—N1—C13	173.29 (11)
O1—C14—C15—C16	-27.02 (17)	C12—C13—N1—C14	-119.05 (12)
N1—C14—C15—C16	154.97 (11)	C4—C13—N1—C14	113.28 (12)
C14—C15—C16—C17	-177.07 (10)	O2—C17—N2—C18	6.93 (18)
C15—C16—C17—O2	32.32 (16)	C16—C17—N2—C18	-170.97 (10)
C15—C16—C17—N2	-149.74 (11)	C25—C18—N2—C17	-121.69 (12)
N2—C18—C19—C24	150.44 (11)	C19—C18—N2—C17	109.67 (12)
C25—C18—C19—C24	21.98 (15)		

Hydrogen-bond geometry (Å, º)

D—H···*A*	*D*—H	H···*A*	*D*···*A*	*D*—H···*A*
C15—H15*A*···O2^{i}	0.99	2.63	3.4536 (15)	141
N1—H1*N*···O2^{i}	0.883 (16)	2.150 (16)	3.0189 (14)	167.4 (14)
N2—H2*N*···O1^{ii}	0.898 (16)	1.997 (16)	2.8937 (14)	175.8 (14)

Symmetry codes: (i) -*x*+1, -*y*+1, -*z*; (ii) -*x*+1, -*y*+1, -*z*+1.

Document origin: *publCIF* [Westrip, S. P. (2010). *J. Apply. Cryst.*, **43**, 920-925].

S9 Computational Details

All calculations were carried out using the Amsterdam Density Functional (ADF) 2023.101 module of the Amsterdam Modeling Suite.[5–7] All stationary points and energies were calculated at the BLYP level of the generalized gradient approximation (GGA) exchange functional developed by Becke (B[4]), and the GGA correlation functional developed by Lee, Yang, and Parr (LYP[5]) (see Tables S4-S6 for Cartesian coordinates). The DFT-D3(BJ) method developed by Grimme and co-workers,[8] which contains the damping function proposed by Becke and Johnson,[9] is used to describe non-local dispersion interactions. In addition, relativistic effects are accounted for through the zeroth-order regular approximation (ZORA) method.[10] This level of theory is referred to as ZORA-BLYP-D3(BJ)/TZ2P and has been proven to accurately describe weak interactions.[11,12] A large uncontracted relativistically-optimized TZ2P basis set was used with no frozen-core approximation consisting of Slater type orbitals (STOs) which is of triple- ζ quality for all atoms and has been augmented with the following sets of polarization functions: *p* and *d* functions on H, *d* and *f* functions on N, C, O, F, S, Cl, and I.[13] The molecular density is fitted by the systematically improvable Zlm fitting scheme and numerical integration is performed on a Becke grid.[14,15] Both are specified with the "VeryGood" option.

Conformational analyses of the receptor were performed using CREST[16] at a temperature of 100°C. Conformers with an energy of +2.0 kcal mol^{-1} relative to the lowest one were neglected and duplicates were removed based on their RSMD (<0.05). The conformers were optimized at the above DFT level in acetonitrile (using the implicit solvation model COSMO[17–20] with Allinger's atom radii[21]) for which the atom radii of Cl and I were changed to match the experimental solvation energies of Cl^- and I^-[22] as closely as possible, resulting in a radius 1.482 Å for Cl (1.735 is default) and 2.010 for I Å (1.967 is default), see earlier work[23] for the procedure.

To confirm that the stationary points are in their lowest energy conformation, frequency analyses have been carried out with zero imaginary frequencies for equilibrium geometries.[24–26] Thermostatistical corrections were applied to calculate the enthalpy H, entropy S, and Gibbs free energy for which the low frequency interpolation scheme of Grimme and co-workers was considered, using their default parameters.[27]

S9.1. Bond Energy Analysis

The complexation energy, ΔE, of an anion-receptor complex is defined as shown in Equation (1), where $E_{complex}$ represents the energy of the interacting anion bonded to the receptor, E_{anion} represents the energy of the anion, and $E_{receptor}$ the energy of the receptor.

$$\Delta E = E_{complex} - (E_{anion} + E_{receptor}) \quad (1)$$

The complexation energy between the anion receptor and anion in solution can be partitioned as formulated in Equation (2), which is based on the activation strain model (ASM)[28–31] for gas-phase calculations, and extended to the solvated phase[32]. In this model, ΔE comprises four components, ΔE_{desolv}, ΔE_{strain}, ΔE_{int} and ΔE_{desolv}:

$$\Delta E = \Delta E_{desolv} + \Delta E_{strain} + \Delta E_{int} + \Delta E_{solv} \quad (2)$$

The first term is the desolvation energy that stems from desolvating the anion and receptor separately, which yields a positive term. The second term is the strain energy (ΔE_{strain}) which indicates how much energy is required to deform the equilibrium geometry of the receptor to the geometry it acquires when it interacts in the complex. The deformed receptor and anion interact and form a complex (in the geometry it adopts in acetonitrile), resulting in the interaction energy ΔE_{int}. Finally, the solvation term ΔE_{solv} accounts for solvating the complex in acetonitrile. Note that during all these steps, equilibrium geometries have been used for the receptor and complex that are obtained in acetonitrile. The above energy terms are calculated by Equations (3–6):

$$\Delta E_{desolv} = (E_{receptor,\,gas} + E_{anion,\,gas}) - (E_{receptor,\,ACN} + E_{anion,\,ACN}) \quad (3)$$

$$\Delta E_{strain} = (E_{deformed\_receptor,\,gas} + E_{anion,\,gas}) - (E_{receptor,\,gas} + E_{anion,\,gas}) \quad (4)$$

$$\Delta E_{int} = E_{complex,\,gas} - (E_{deformed\_receptor,\,gas} + E_{anion,\,gas}) \quad (5)$$

$$\Delta E_{solv} = E_{complex,\,ACN} - E_{complex,\,gas} \quad (6)$$

In the framework of the Kohn-Sham molecular orbital model using quantitative canonical energy decomposition analysis (EDA),[33,34] the latter term, $\Delta E_{int,}$ can be further decomposed into electrostatic interactions (ΔV_{elstat}), Pauli repulsion (ΔE_{Pauli}), orbital interactions (ΔE_{oi}), and an additional term that accounts for dispersion interactions ΔE_{disp}, as shown in Equation (7).

$\Delta E_{int} = \Delta V_{elstat} + \Delta E_{Pauli} + \Delta E_{oi} + \Delta E_{disp}$ (7)

The term ΔV_{elstat} represents the quasi-classical Coulomb interaction between the unperturbed charge distributions of the deformed fragments. ΔE_{Pauli} comprises destabilizing interactions between occupied orbitals on each fragment and is responsible for steric repulsion. The orbital interaction energy (ΔE_{oi}) accounts for donor-acceptor interactions and polarization effects, including interactions between the highest occupied and lowest unoccupied MOs (HOMO–LUMO). Finally, ΔE_{disp} accounts for dispersion corrections as introduced by Grimme *et al.*[8]

For a closer connection to experimental results, the coordination strength can also be expressed in enthalpy, entropy, and Gibbs free energy:

$\Delta H = E_{complex} - (H_{anion} + H_{receptor})$ (8)

$\Delta S = S_{complex} - (S_{anion} + S_{receptor})$ (9)

$\Delta G = G_{complex} - (G_{anion} + G_{receptor})$ (10)

where $H_{complex}$, $S_{complex}$ and $G_{complex}$, are the enthalpies, entropies and Gibbs free energies of the interacting complex, respectively, and $H_{anion/receptor}$, $S_{anion/receptor}$ and $G_{anion/receptor}$ those of the anion and receptor.

S9.2. Voronoi deformation density charge analysis

The Voronoi deformation density (VDD) method was considered for computing atomic charges and analyzing the electron density distribution.[35] The VDD atomic charge on atom A in a molecule (Q_A^{VDD}) is computed as the (numerical) integral of the deformation density in the volume of the Voronoi cell of A [Equation (11)]. The Voronoi cell of A is defined as the compartment of space bounded by the bond midplanes on and perpendicular to all bond axes between nucleus A and its neighbouring nuclei.

$$Q_A^{\mathrm{VDD}} = -\int_{Voronoi\ cell\ of\ A} \left[\rho(\boldsymbol{r}) - \rho_{promolecule}(\boldsymbol{r})\right] d\boldsymbol{r} \quad (11)$$

Here, the deformation density is the difference between ρ(r), *i.e.*, the electron density of the overall molecule or complex, and $\rho_{promolecule}(r) = \sum_Y \rho_Y(r)$, *i.e.*, the superposition of spherical average-of-configuration atomic densities $\rho_Y(r)$ of each atom Y in the fictitious promolecule without chemical interactions, in which all atoms are considered neutral. The terms $\rho_{promolecule}(r)$ and $\rho_Y(r)$ on itself do not have a clear and distinct meaning. Instead, the deformation density, that is the difference

$\rho(r) - \rho_{promolecule}(r)$, measures the charge flow to or from a nucleus A. The interpretation of the VDD charge Q_A^{VDD} is now straightforward and transparent: instead of measuring the amount of charge associated with A, Q_A^{VDD} directly monitors how much charge flows out of ($Q_A^{VDD} > 0$) or into ($Q_A^{VDD} < 0$) the Voronoi cell of A due to interactions with neighboring nuclei and electrons.

S10 Figures and Tables from Computational Study

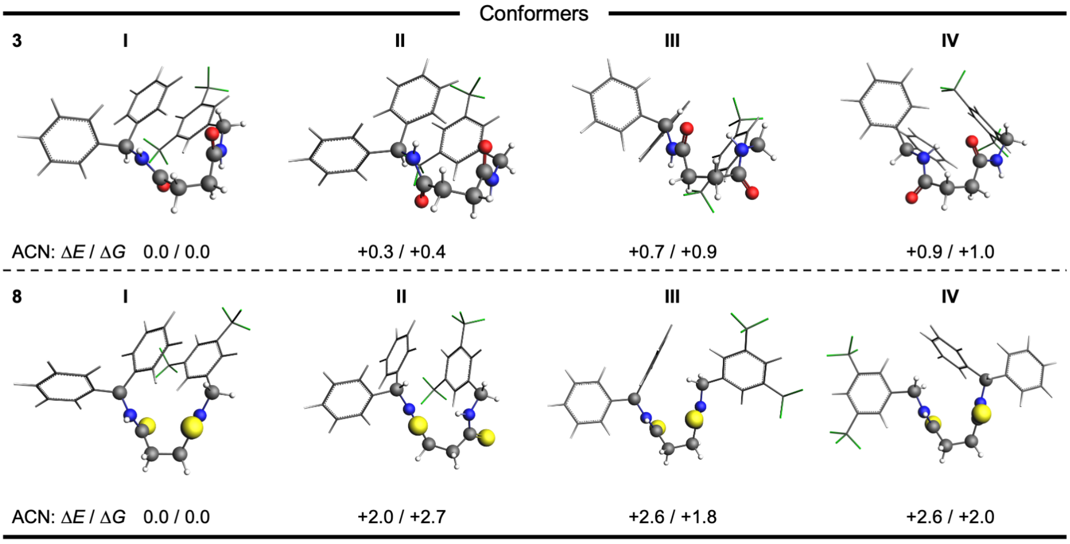

Figure S39. Conformers of receptors 3 and 8 ordered by relative energies Δ*E* with (Gibbs free) energies (in kcal mol^{-1}), optimized at COSMO(ACN)-ZORA-BLYP-D3(BJ)/TZ2P.

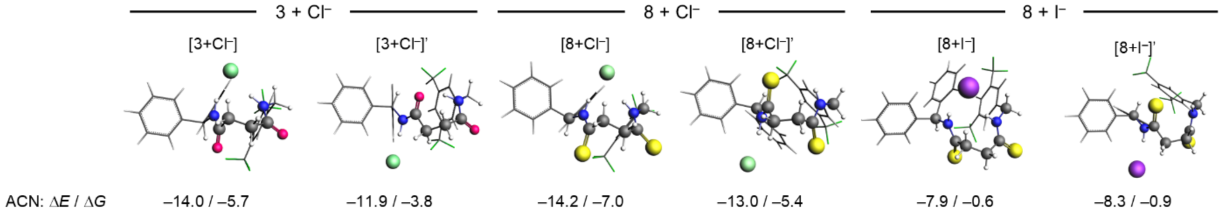

Figure S40. Overview of all relevant receptor – anion complexes with (Gibbs free) energies (in kcal mol^{-1}), optimized at COSMO(ACN)-ZORA-BLYP-D3(BJ)/TZ2P.

Table S5. Molecular orbital (MO) interaction analysis for the interaction between receptor 8 and anions Cl^- and I^-. The distances (in Å) between the interacting N(H) groups and anion are tabulated in addition to the orbital overlap S (*i.e.*, ⟨n*p* anion | σ^*_{NH} receptor⟩ in arbitrary units), energy gap (eV), and stabilization S^2 / $\Delta\varepsilon$ (eV^{-1}) – a measure for the strength of the orbital interaction as illustrated by Albright.[36] Computed at COSMO(ACN)-ZORA-BLYP-D3(BJ)/TZ2P.

System	Schematic MOs	DFT MOs	S	$\Delta\varepsilon$	S^2 / $\Delta\varepsilon$ * 10^2
**[8 + Cl]**	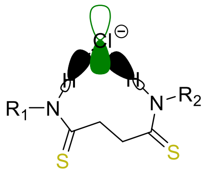	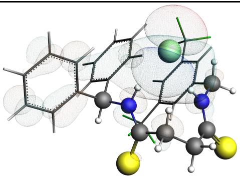	0.163	1.60	1.66
**[8 + I]**	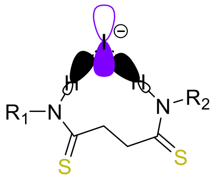	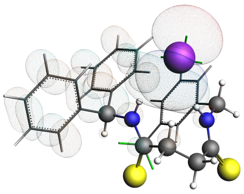	0.135	1.94	0.94

S11 Cartesian Coordinates

Table S6. Cartesian coordinates (in Å), absolute energies, enthalpies, and Gibbs free energies (in kcal mol^{-1}) for the anions Cl^- and X^-, computed at COSMO(ACN)-ZORA-BLYP-D3(BJ)/TZ2P.

Cl^- (ACN)
E = –148.30 kcal mol^{-1}
H = –146.82 kcal mol^{-1}
G = –157.73 kcal mol^{-1}
N_{imag} = 0

```
Cl       0.00000000      0.00000000      0.00000000
```

I^- (ACN)
E = –130.87 kcal mol^{-1}
H = –129.39 kcal mol^{-1}
G = –141.44 kcal mol^{-1}
N_{imag} = 0

```
I        0.00000000      0.00000000      0.00000000
```

Table S7. Cartesian coordinates (in Å), absolute energies, enthalpies, and Gibbs free energies (in kcal mol^{-1}) for the conformers of receptors 3 and 8, computed at COSMO(ACN)-ZORA-BLYP-D3(BJ)/TZ2P.

3-I (ACN)

E = –8223.71 kcal mol^{-1}

H = –7940.21 kcal mol^{-1}

G = –7999.21 kcal mol^{-1}

N_{imag} = 0

C	9.21332578	11.26031846	6.22030709
H	6.96989226	9.96078216	14.33681095
H	10.24898626	11.10662983	5.92553439
C	3.14607528	6.75987493	8.58465672
C	8.47209411	12.30848329	5.66020823
H	5.39783922	9.58367490	13.60634157
H	8.92989181	12.97116335	4.92935567
F	3.47922200	8.07718733	8.69660116
C	7.13761912	12.49571370	6.04014060
C	7.03631410	8.29125725	12.99983924
H	6.55400358	13.30377919	5.60477560
N	7.01096703	10.18772450	9.93933872
C	6.55051695	11.64115250	6.97987426
C	6.43961416	5.96106165	12.37780178
H	5.51397511	11.78960672	7.27664408
F	3.73598397	1.70800066	8.89682260
C	7.28719162	10.58979550	7.54393974
H	6.37007918	5.24569996	13.20722422
C	8.62167681	10.40242531	7.15335887
H	7.97536298	10.45750680	10.09880180
H	9.19593326	9.57865174	7.57117656
H	7.48686631	5.98559069	12.05813032
C	7.50345403	5.48539975	7.96447690
H	7.69194771	4.42678502	7.80485621
C	5.56405235	5.48008343	11.23259626
N	6.11646658	7.30404862	12.84008976
C	4.79805260	6.35145695	10.44898433
F	5.06478245	1.36026825	10.61920654
H	4.79974173	7.41805242	10.65035943
H	5.15062320	7.51249533	13.06783525
C	6.43136968	7.55236234	7.29431608
C	4.03879475	5.84706231	9.38766125
H	5.79078751	8.09920206	6.60561606
H	3.45253205	4.10272713	8.25426665
C	6.97579961	8.21358636	8.40616103
C	4.04017666	4.48394647	9.08182265
C	7.79387214	7.49934044	9.28768769
O	4.94283798	9.92973186	10.87921791
H	8.20403952	7.98367679	10.16867358

C	4.87140328	2.15563981	9.51958671
C	8.05964195	6.14219533	9.06410656
O	8.25158913	8.12749969	12.77952206
H	8.68929444	5.59605871	9.76277707
C	4.80858742	3.62124203	9.86574106
C	6.64594067	9.69529565	8.60247173
H	6.23637900	5.69303986	6.22505295
H	5.55882515	9.80370132	8.54117218
C	5.55941328	4.11207280	10.93701770
C	6.14122541	10.25102843	10.98080348
F	3.14952870	6.45382391	7.24549290
C	6.74234771	10.70293875	12.31091646
H	6.14745347	3.42926723	11.54386838
H	6.26896909	11.64871540	12.59772266
F	5.91729666	1.87976199	8.65763760
H	7.81837619	10.87626352	12.21602889
F	1.82515431	6.65706270	8.99079548
C	6.47234819	9.64850675	13.41253822
C	6.68560646	6.19745924	7.07720376
H	3.19153672	0.82418312	1.40561384
C	-1.92515272	1.07682799	3.91306099
C	0.41865224	-1.66080138	-1.46945840
H	-1.64090903	0.03217785	4.00794562
H	-0.95323973	-1.20030173	-3.07300267
C	-1.51839684	1.81356945	2.79402444
C	-0.03145530	-0.93300992	-2.57311335
C	-1.88231042	3.16434065	2.69538602
O	1.69407278	1.26657317	3.02176733
H	-1.56792140	3.74428535	1.82953051
C	0.28184452	0.98039988	-4.18749997
C	-2.64379086	3.77029383	3.69908354
O	4.30981117	-2.12554980	1.04233936
H	-2.91489295	4.82015952	3.61251507
C	0.72720992	0.15530065	-3.00658425
C	-0.73533326	1.17535178	1.64070014
H	-2.99666240	1.09788405	5.78462379
H	-0.02091549	1.91923862	1.27409077
C	1.91076268	0.51609589	-2.34957360
C	1.21151671	0.14623960	2.76107328
F	-1.57192701	-2.99968476	-1.54119156
C	1.89903006	-1.14491282	3.20520925
H	2.48413679	1.37184571	-2.69635077
H	1.49388358	-2.01066988	2.66925164
F	-0.87001783	0.52253426	-4.76068304
H	1.65609318	-1.28409592	4.26637938
F	0.34184671	-4.03879880	-1.22950551
C	3.43958818	-1.07570086	3.05546330
C	-2.68881371	1.68087810	4.91935808

3-II (ACN)

E = –8223.42 kcal mol^{-1}

H = –7939.81 kcal mol^{-1}

G = –7998.80 kcal mol^{-1}

N_{imag} = 0

C	6.48286324	12.51952020	6.96056314
H	7.99349286	9.10464973	14.46311121
H	7.26156633	13.16199265	6.55551681
C	6.86153735	3.04989284	8.09850200
C	5.13303490	12.82089749	6.72936659
H	6.25975097	8.93060250	14.13958531
H	4.86224356	13.69818957	6.14627524
F	6.38851937	1.78731612	8.40687944
C	4.13745628	11.98872566	7.25143403
C	7.55120266	7.58935439	13.02098337
H	3.08814175	12.21586790	7.07674660
N	6.95493571	9.68922104	10.26633976
C	4.49042030	10.86034358	8.00341555
C	6.70171993	5.30363834	12.58701330
H	3.71559636	10.21507964	8.41123229
F	3.61172376	7.11410267	7.65748433
C	5.83626815	10.55326880	8.23558442
H	6.14000127	4.61457040	13.22700948
C	6.83059877	11.39083746	7.70679263
H	7.96007078	9.80058991	10.19662090
H	7.88033597	11.15654534	7.87247546
H	7.75221259	5.00184522	12.61097233
C	8.29190939	6.25509225	6.77371581
H	8.79880001	5.47994204	6.20445857
C	6.17994672	5.19640502	11.15783407
N	6.59278317	6.63752846	13.17026013
C	6.73934919	4.24848034	10.29786610
F	3.12717479	7.66761275	9.73218149
H	7.56860748	3.63497470	10.63937028
H	5.76436193	6.86676361	13.70614855
C	6.71010049	8.08954486	6.88413054
C	6.25427726	4.10111836	8.99318492
H	5.99259371	8.73766025	6.38937557
H	4.82501099	4.77297600	7.52240527
C	6.97394443	8.26002277	8.25250705
C	5.19696643	4.88486785	8.53420847
C	7.89743400	7.40568778	8.87409980
O	5.14930408	9.70318442	11.66737910
H	8.09801372	7.49302146	9.93792126
C	3.43567662	6.60986453	8.92238771
C	8.55586122	6.41602605	8.13668299
O	8.59318408	7.39013356	12.36955725
H	9.26533140	5.76063643	8.63557129
C	4.63156479	5.82498360	9.40191750
C	6.21116212	9.32042047	9.05385128
H	7.13781957	6.97198819	5.09300212

H	5.28009869	8.87998185	9.42465611
C	5.12174842	5.99171849	10.69993521
C	6.37443822	9.82932647	11.48512052
F	6.58837379	3.25126205	6.77455090
C	7.33941761	10.08423184	12.64133869
H	4.68902439	6.74693764	11.34755120
H	7.05790548	11.02531857	13.12593638
F	2.30335253	5.82054443	8.86244611
H	8.36750155	10.17961185	12.27997840
F	8.22679734	2.98075803	8.22131293
C	7.25032123	8.93633661	13.67539079
C	7.35925837	7.09234320	6.15116412

3-III (ACN)

E = –8223.03 kcal mol^{-1}

H = –7939.51 kcal mol^{-1}

G = –7998.34 kcal mol^{-1}

N_{imag} = 0

C	-3.49802980	-0.52814474	-0.39684354
H	3.80454272	-0.16453578	3.54052006
H	-4.21834512	-1.33354101	-0.27633347
C	-0.33971783	-2.86687699	-0.97822649
C	-3.45971972	0.20851246	-1.58724808
H	3.89353388	-1.94000944	3.54438565
H	-4.15039833	-0.02278860	-2.39457290
F	-0.52472716	-2.83393713	0.39307561
C	-2.52824211	1.24071340	-1.73328976
C	3.84890163	-1.10289360	1.58503694
H	-2.48988438	1.81435574	-2.65525879
N	0.05314746	0.00880781	2.07283426
C	-1.63197449	1.52882254	-0.69852192
C	3.67486243	0.14025794	-0.56254733
H	-0.89738731	2.32097190	-0.82254488
F	1.23608720	1.01179062	-5.18050738
C	-1.67019171	0.80106224	0.49701499
H	4.46164240	-0.54229488	-0.90071134
C	-2.61635585	-0.22521732	0.64337765
H	-0.27661422	-0.91935919	1.83169954
H	-2.67644316	-0.78488102	1.57421493
H	3.97032820	1.15713436	-0.83181470
C	-3.05184369	3.02828717	4.81539455
H	-3.64272930	3.49803301	5.59830651
C	2.36256977	-0.22021993	-1.25026852
N	3.60065967	0.04469929	0.89586983
C	1.60432197	-1.31387803	-0.81660517
F	0.05688664	2.29612967	-3.84001693
H	1.94574430	-1.90767706	0.02354599
H	3.19153672	0.82418312	1.40561384

C	-1.92515272	1.07682799	3.91306099
C	0.41865224	-1.66080138	-1.46945840
H	-1.64090903	0.03217785	4.00794562
H	-0.95323973	-1.20030173	-3.07300267
C	-1.51839684	1.81356945	2.79402444
C	-0.03145530	-0.93300992	-2.57311335
C	-1.88231042	3.16434065	2.69538602
O	1.69407278	1.26657317	3.02176733
H	-1.56792140	3.74428535	1.82953051
C	0.28184452	0.98039988	-4.18749997
C	-2.64379086	3.77029383	3.69908354
O	4.30981117	-2.12554980	1.04233936
H	-2.91489295	4.82015952	3.61251507
C	0.72720992	0.15530065	-3.00658425
C	-0.73533326	1.17535178	1.64070014
H	-2.99666240	1.09788405	5.78462379
H	-0.02091549	1.91923862	1.27409077
C	1.91076268	0.51609589	-2.34957360
C	1.21151671	0.14623960	2.76107328
F	-1.57192701	-2.99968476	-1.54119156
C	1.89903006	-1.14491282	3.20520925
H	2.48413679	1.37184571	-2.69635077
H	1.49388358	-2.01066988	2.66925164
F	-0.87001783	0.52253426	-4.76068304
H	1.65609318	-1.28409592	4.26637938
F	0.34184671	-4.03879880	-1.22950551
C	3.43958818	-1.07570086	3.05546330
C	-2.68881371	1.68087810	4.91935808

3-IV (ACN)

E = –8222.80 kcal mol^{-1}

H = –7939.27 kcal mol^{-1}

G = –7998.22 kcal mol^{-1}

N_{imag} = 0

C	4.50789558	1.65360266	-1.45122843
H	0.67637346	-5.09098177	-1.08640583
H	4.30323864	2.01918170	-2.45488091
C	-4.61528360	1.06575801	-0.24012596
C	5.70146484	2.00412812	-0.80517046
H	0.12516720	-4.13336272	0.27802330
H	6.42674584	2.64063807	-1.30688566
F	-5.39971801	-0.05177985	-0.27369038
C	5.95438932	1.53363896	0.48777199
C	0.52337460	-3.01754049	-1.53338824
H	6.87735239	1.80304465	0.99632693
N	2.65907089	-1.78195378	0.42762159
C	5.01873269	0.71299192	1.13120305
C	-1.31584481	-1.84774200	-2.73145613

H	5.21753213	0.34693099	2.13653216
F	-0.41688249	3.62810107	-2.94436544
C	3.82386565	0.36120889	0.49167361
H	-2.19580778	-2.29287052	-3.20396473
C	3.57460391	0.84126449	-0.80323029
H	2.48163063	-1.72231312	-0.57685796
H	2.64187531	0.59025362	-1.30210677
H	-0.54651322	-1.70436677	-3.49269346
C	-0.98237027	1.44304780	2.02951385
H	-1.92420230	1.93832840	2.24986826
C	-1.68670805	-0.51209221	-2.10707945
N	-0.79743984	-2.81597762	-1.75352960
C	-2.93286852	-0.35054373	-1.48674967
F	1.07780658	2.63216933	-1.67416354
H	-3.62928674	-1.18256572	-1.45043475
H	-1.46611704	-3.38805596	-1.24897643
C	1.43256720	1.51286887	1.82011819
C	-3.28553719	0.87800194	-0.92591469
H	2.36241961	2.07207017	1.88287231
H	-2.68540289	2.92072084	-0.54980601
C	1.45752199	0.16317464	1.43406676
C	-2.40810727	1.96587154	-0.98220827
C	0.24424133	-0.53212394	1.34381551
O	2.65797281	-3.22035067	2.21300239
H	0.23703211	-1.57348532	1.04105833
C	-0.24247657	2.98583177	-1.73028500
C	-0.96689587	0.10106111	1.64044113
O	1.40524406	-2.34925299	-2.11180732
H	-1.89726898	-0.45419625	1.55663178
C	-1.16780548	1.80227709	-1.59777974
C	2.80653014	-0.53511372	1.18726015
H	0.22254050	3.19641796	2.40575775
H	3.20986269	-0.82838962	2.16290491
C	-0.80213451	0.56941338	-2.15288526
C	2.56072311	-3.01280835	0.98613439
F	-4.46096771	1.40100294	1.08926387
C	2.29305557	-4.14186482	-0.01267552
H	0.16562539	0.45039561	-2.63036389
H	2.48207189	-5.08459330	0.50591689
F	-0.44778917	3.93271215	-0.76459889
H	2.98956955	-4.06534943	-0.85458602
F	-5.34854126	2.08600620	-0.80334185
C	0.84570850	-4.14876446	-0.54897534
C	0.22331787	2.14817457	2.11615045

8-I (ACN)

E = –8070.02 kcal mol^{-1}

H = –7788.68 kcal mol^{-1}

G = –7848.89 kcal mol^{-1}

N_{imag} = 0

C	9.36222731	11.23325541	6.32024094
H	7.01832424	10.01194713	14.23707810
H	10.36601920	10.97085487	5.99403559
C	2.90051413	6.36259236	8.68870314
C	8.77515398	12.42970384	5.89192748
H	5.46193342	9.51128201	13.54413717
H	9.32087586	13.09980491	5.23169804
F	3.11555344	7.70647713	8.70461939
C	7.47989194	12.75776860	6.31174149
C	7.17095344	8.38022568	12.86202448
H	7.01543230	13.68296924	5.97832240
N	6.90881400	10.19763237	9.93531166
C	6.77938604	11.89376227	7.15904560
C	6.67803997	6.06606777	12.09405449
H	5.77394864	12.15018877	7.48691205
F	4.45960366	1.53121783	8.62115037
C	7.36231101	10.69208856	7.59038713
H	6.79227468	5.40292152	12.96157706
C	8.65749022	10.36618412	7.16270085
H	7.88586486	10.39964012	10.13307448
H	9.11304542	9.43111864	7.47785842
H	7.66635417	6.15128438	11.62938959
C	7.21637752	5.58397015	7.72548214
H	7.36353326	4.52873387	7.50988027
C	5.69480316	5.45696428	11.11647965
N	6.30972068	7.40291828	12.54845451
C	4.77803915	6.21313236	10.37486656
F	4.36769781	1.28394314	10.80750918
H	4.71864466	7.29079659	10.49555667
H	5.32330596	7.57879267	12.72376444
C	6.08220606	7.67976734	7.28580098
C	3.94155984	5.57923291	9.45033831
H	5.34833507	8.25235440	6.72280562
H	3.36614055	3.71910032	8.51160141
C	6.80981955	8.30473576	8.30918284
C	4.01197058	4.19983921	9.23738371
C	7.74310313	7.55648477	9.03599135
S	4.37022950	10.11174071	10.78586940
H	8.30151980	8.01267666	9.84886995
C	5.01821365	1.95927430	9.79415873
C	7.94704395	6.20310979	8.74257612
S	8.84767733	8.24929905	12.70551541
H	8.66990116	5.63103081	9.31962032
C	4.93409653	3.45450189	9.97259436
C	6.58569719	9.79379626	8.55213909
H	5.69370111	5.85127895	6.21541414
H	5.51648126	9.99648159	8.43780572
C	5.76077737	4.07385045	10.91383186
C	6.03051205	10.37599742	10.93090781
F	2.82146838	5.98009409	7.37028941
C	6.65057346	10.79090251	12.25387421

H	6.47018045	3.48105483	11.48479737
H	6.13061489	11.67971564	12.62340775
F	6.31524863	1.50672532	9.80799531
H	7.70917984	11.03880783	12.12089033
F	1.63566066	6.16085085	9.21559048
C	6.52246891	9.67281671	13.32358534
C	6.27962303	6.32771505	6.99738503

8-II (ACN)

E = –8068.06 kcal mol^{-1}

H = –7786.86 kcal mol^{-1}

G = –7846.24 kcal mol^{-1}

N_{imag} = 0

C	-3.36745100	-0.29306665	-0.48065994
H	3.93551937	-0.14453265	3.43003054
H	-4.14019973	-1.05471921	-0.41423441
C	-0.48234182	-2.82084835	-0.87694702
C	-3.18277486	0.41842438	-1.67245010
H	3.92000631	-1.92021846	3.46470781
H	-3.81262201	0.21122997	-2.53410890
F	-0.64501433	-2.69593521	0.49179123
C	-2.18391250	1.39329346	-1.75113539
C	3.82763883	-1.09804917	1.49610676
H	-2.03383569	1.94767114	-2.67341488
N	0.14398053	0.17129562	2.16199321
C	-1.36840320	1.65245482	-0.64489508
C	3.55510374	0.17991306	-0.63581124
H	-0.58553821	2.40445938	-0.71113187
F	1.22241067	0.39086063	-5.46088094
C	-1.55735388	0.95335520	0.55403316
H	4.37457032	-0.43634533	-1.02345725
C	-2.56647235	-0.01885861	0.63033907
H	-0.15225193	-0.75627823	1.86652302
H	-2.73986059	-0.55268340	1.56195430
H	3.77586609	1.22702912	-0.85623226
C	-3.27029516	2.76598798	4.96093960
H	-3.92181863	3.13896092	5.74773404
C	2.25308610	-0.23380333	-1.31183324
N	3.52324134	0.02033011	0.81727801
C	1.45915471	-1.25996610	-0.79113928
F	0.28373656	2.01876006	-4.31733576
H	1.75333520	-1.75942529	0.12306120
H	3.18749024	0.81847186	1.36011425
C	-1.94302321	0.97569154	4.00012194
C	0.30001591	-1.67014597	-1.45523798
H	-1.56416879	-0.04128915	4.05600930
H	-0.99108179	-1.38339284	-3.16246106
C	-1.58114499	1.80294663	2.93087301
C	-0.09031759	-1.06810057	-2.65213499

C	-2.06820348	3.11699465	2.88465070
S	1.89925089	1.77950753	3.35942811
H	-1.78749546	3.76579154	2.05705619
C	0.31674650	0.64868531	-4.45389502
C	-2.90779871	3.59775364	3.89297592
S	4.43977323	-2.49757011	0.78118536
H	-3.27520590	4.62052692	3.84834017
C	0.69872574	-0.03687796	-3.16605183
C	-0.71303886	1.30831731	1.77106602
H	-3.05635023	0.80343913	5.83892584
H	-0.03039026	2.11958747	1.49881168
C	1.85968113	0.38144081	-2.50467349
C	1.27523980	0.28840223	2.87035296
F	-1.72495843	-2.95809597	-1.41489096
C	1.96392998	-1.02301665	3.22644012
H	2.45810558	1.18909108	-2.91782648
H	1.48973785	-1.85885581	2.69656580
F	-0.90941101	0.26865102	-4.92110001
H	1.79729872	-1.18913652	4.29764151
F	0.16603189	-4.02358166	-1.06251658
C	3.49631297	-1.04168511	2.97818427
C	-2.78438519	1.45513080	5.01163937

8-III (ACN)

E = –8067.42 kcal mol^{-1}

H = –7786.15 kcal mol^{-1}

G = –7846.91 kcal mol^{-1}

N_{imag} = 0

C	7.05380588	-1.44465331	0.05506520
H	-0.11374554	-4.33608611	-1.51936610
H	7.65536411	-2.31524925	-0.19640613
C	-5.33751177	-1.07861246	-2.27035586
C	7.65541358	-0.18752766	0.18374097
H	-0.33200762	-4.00424780	0.21285919
H	8.72606771	-0.07421900	0.03027542
F	-6.29984582	-0.47667286	-3.03335718
C	6.86830085	0.92274374	0.51027419
C	-0.17003698	-2.24663559	-1.03219245
H	7.32492197	1.90452770	0.61363924
N	2.83416772	-1.77568196	0.07181556
C	5.48992942	0.77847657	0.70069476
C	-0.91518877	-0.10767081	-0.00564917
H	4.88912763	1.64852478	0.94892615
F	-5.55519370	3.83082521	-0.85669155
C	4.87917010	-0.47846247	0.56967283
H	-0.14108088	0.35447835	-0.62627189
C	5.67600570	-1.58926193	0.24990028
H	2.88767437	-1.69537368	-0.94171902
H	5.22271339	-2.57177252	0.15128733

H	-0.77961390	0.24507446	1.01927120
C	1.05574256	2.95177549	0.21803185
H	0.47915227	3.86054206	0.06275185
C	-2.27765837	0.32363075	-0.52011052
N	-0.67653966	-1.54659489	-0.00558072
C	-3.16002561	-0.55738195	-1.14705463
F	-3.58755669	4.34484810	-1.70035121
H	-2.89138196	-1.60161471	-1.26240695
H	-0.80962554	-2.03529151	0.87599638
C	2.44721545	1.17310118	-0.66376958
C	-4.38989805	-0.09460642	-1.63157208
H	2.95324656	0.71084270	-1.50813529
H	-5.71137591	1.59772950	-1.86976619
C	2.55567127	0.60892386	0.61693742
C	-4.75825512	1.24480840	-1.49500586
C	1.90585643	1.22609543	1.69289505
S	1.98100845	-3.15438216	2.21160819
H	1.98504355	0.79247088	2.68717985
C	-4.21636229	3.58387773	-0.73237012
C	1.16064416	2.39555448	1.49680093
S	0.15651962	-1.57844184	-2.54340289
H	0.66291958	2.86692242	2.34080468
C	-3.87153737	2.12160175	-0.86273659
C	3.38285059	-0.64959756	0.85013138
H	1.61783215	2.76129838	-1.86100378
H	3.25787998	-0.95056018	1.89658830
C	-2.64047634	1.66880362	-0.38091898
C	2.23600608	-2.86692334	0.56760211
F	-4.68764582	-1.97766310	-3.07955712
C	1.72192303	-3.83131867	-0.48736010
H	-1.95911659	2.36030885	0.10726653
H	1.92765521	-4.85502020	-0.16598708
F	-3.82083846	4.10714999	0.47426152
H	2.22826507	-3.65661091	-1.44320096
F	-6.00577210	-1.83277481	-1.32391839
C	0.18616196	-3.68227793	-0.69718324
C	1.69858229	2.33548285	-0.86396973

8-IV (ACN)

E = –8067.44 kcal mol^{-1}

H = –7786.16 kcal mol^{-1}

G = –7847.07 kcal mol^{-1}

N_{imag} = 0

C	7.04203833	-1.27185344	-0.14031666
H	-0.12375703	-4.25747218	-1.54128473
H	7.64968852	-2.11177860	-0.46946985
F	-5.46727624	-1.77201223	-2.76258145
C	7.62599135	-0.01486397	0.05371770
H	-0.22235564	-4.04481352	0.22025308

H	8.68888522	0.12912029	-0.12582727
C	-5.60155175	-1.24573901	-1.49133364
C	6.83131287	1.05575070	0.47969763
C	-0.20298211	-2.20670557	-0.91501896
H	7.27421270	2.03707546	0.63437725
N	2.83119066	-1.69756927	0.01165433
C	5.46281975	0.87228091	0.70396874
C	-0.97339940	-0.16682630	0.27141997
H	4.85565363	1.71187363	1.02981060
F	-4.69431983	4.08913993	-0.72467370
C	4.86976663	-0.38478660	0.50791315
H	-0.15892597	0.38973523	-0.19823533
C	5.67399009	-1.45591017	0.08818917
H	2.79602685	-1.54228454	-0.99403699
H	5.23446159	-2.43800445	-0.06228594
H	-0.99210290	0.09086410	1.33423301
C	0.98381648	2.98548032	0.44130401
H	0.39020418	3.89195680	0.34983657
C	-2.28562220	0.23953730	-0.37886331
N	-0.66305531	-1.59139014	0.18515162
C	-3.31702250	-0.67182482	-0.61110007
F	-4.48754591	3.62285443	-2.86476741
H	-3.18356076	-1.71684847	-0.35197510
H	-0.70805297	-2.13100897	1.04512475
C	2.36927927	1.27193333	-0.56937890
C	-4.51809609	-0.24125341	-1.18674154
H	2.85501848	0.85952812	-1.45073245
H	-5.63613268	1.42582775	-1.98698205
C	2.52959559	0.65060968	0.67906175
C	-4.70581854	1.09740219	-1.53664774
C	1.90576208	1.20524441	1.80361418
S	2.21939468	-3.27015728	2.10096076
H	2.02478130	0.72708676	2.77320646
C	-3.87894449	3.45880875	-1.64493084
C	1.13777752	2.37028962	1.68780251
S	0.00611745	-1.42525192	-2.39099173
H	0.66086491	2.79323206	2.56857613
C	-3.66831681	2.00547593	-1.30002299
C	3.38640047	-0.60097871	0.82600465
H	1.47895692	2.90173692	-1.66197660
H	3.30297072	-0.95432666	1.86009072
C	-2.46735230	1.58341870	-0.72659728
C	2.32637324	-2.85108973	0.46885472
F	-6.85794584	-0.69965411	-1.43386946
C	1.76834683	-3.75944012	-0.61285695
H	-1.66554826	2.29498765	-0.55715901
H	2.02755745	-4.79480001	-0.38029785
F	-2.71218661	4.17272036	-1.68057004
H	2.19786477	-3.50130155	-1.58730031
F	-5.58933189	-2.31185219	-0.63166327

C	0.21801984	-3.64807009	-0.70126828
C	1.59832351	2.43083027	-0.68940521

Table S8. Cartesian coordinates (in Å), absolute energies, enthalpies, and Gibbs free energies (in kcal mol^{-1}) for the complexes of receptors 3 and 8 with anions Cl^- and I^-, computed at COSMO(ACN)-ZORA-BLYP-D3(BJ)/TZ2P.

[3+Cl⁻] (ACN)

E = –8385.88 kcal mol^{-1}

H = –8101.23 kcal mol^{-1}

G = –8162.97 kcal mol^{-1}

N_{imag} = 0

C	3.36260585	4.48354068	-6.78920281
H	1.01817019	2.17850847	0.73017169
H	4.39190476	4.78608287	-6.96950291
C	1.94918245	-4.70670745	-5.13840478
C	2.30529790	5.27587958	-7.25930788
H	-0.45927483	2.24221955	-0.22876976
H	2.51206981	6.19441201	-7.80393451
F	1.74661787	-4.71811376	-6.49080919
C	0.98434854	4.87780200	-7.02738660
C	0.53701470	0.38332753	-0.33057373
H	0.15877432	5.48577150	-7.39112529
N	1.78642040	1.83722774	-3.63810243
C	0.72138398	3.69392493	-6.32540332
C	1.66176437	-1.66819357	-1.13459403
H	-0.30567995	3.38682589	-6.14031442
F	-2.48927012	-2.03702470	-5.46535084
C	1.77355344	2.89850848	-5.85473853
H	1.05164620	-2.16582588	-0.37280687
C	3.09680550	3.30071892	-6.09466620
H	2.75331868	1.77258962	-3.31209342
H	3.92069866	2.68280267	-5.74298227
H	2.69739465	-1.99626443	-1.00838555
C	3.45101525	-1.77265793	-6.92072504
H	3.93561783	-2.61812614	-7.40276014
C	1.16476824	-2.06854385	-2.51907098
N	1.60565781	-0.22918375	-0.89476045
C	1.76690995	-3.13421066	-3.19053248
F	-1.19754062	-0.38654258	-6.12653314
H	2.60705453	-3.65173328	-2.73596314
H	2.42707571	0.30780516	-1.19296748
C	1.87469463	0.06559633	-6.99041230
C	1.30915232	-3.52512812	-4.45543601
H	1.13997992	0.65265572	-7.53666265
H	-0.11247057	-3.17094718	-6.03967929
C	2.18590544	0.40907911	-5.66276350

C	0.23907437	-2.86799051	-5.05998930
C	3.13130607	-0.35659910	-4.97334261
O	-0.36155846	2.36561857	-3.04834097
H	3.39028444	-0.12521950	-3.94507363
C	-1.55445070	-1.12688705	-5.01929321
C	3.76318829	-1.43803684	-5.60221958
O	-0.45358641	-0.25153201	0.09174257
H	4.49343100	-2.02392781	-5.04923806
C	-0.37254584	-1.81042338	-4.37697637
C	1.48059046	1.62180134	-5.06038437
H	2.23411001	-1.27322082	-8.63820348
H	0.40112736	1.44931608	-5.09850998
C	0.08925261	-1.40378016	-3.12443252
C	0.84594070	2.26519831	-2.75611182
F	3.30411101	-4.76554154	-4.93384822
C	1.36124835	2.62754601	-1.36215450
H	-0.38899813	-0.57146779	-2.62249713
H	1.21676524	3.70882941	-1.24327371
F	-2.20430284	-0.27488510	-4.17383820
H	2.43540569	2.43739089	-1.28487459
F	1.44161786	-5.90351464	-4.66132561
C	0.58223641	1.90969971	-0.24080874
C	2.49512650	-1.01720651	-7.61364665
Cl	4.55401373	0.98594046	-1.83417143

[3+Cl⁻]' (ACN)

E = –8383.84 kcal mol^{-1}

H = –8099.04 kcal mol^{-1}

G = –8161.12 kcal mol^{-1}

N_{imag} = 0

C	-7.04682093	0.43339562	-1.94655990
H	0.10224692	1.50101724	1.92152542
H	-7.44711149	-0.57752921	-1.94674513
C	-4.05575515	-1.71010080	-2.85620900
C	-7.44607715	1.34464108	-2.92934510
H	0.08491470	-0.24130401	2.26720824
H	-8.15955319	1.04841373	-3.69492466
F	-4.29139616	-1.93927935	-1.52204356
C	-6.91150356	2.63861777	-2.92640390
C	0.04813957	0.21009962	0.18448995
H	-7.20177637	3.35169798	-3.69398046
N	-3.81443629	1.53078184	0.70747078
C	-5.99069625	3.01229961	-1.94462215
C	0.03485299	1.11783183	-2.13458852
H	-5.57629300	4.01800490	-1.95069729
F	-2.88355859	3.10128708	-6.07218810
C	-5.59075715	2.10407727	-0.95234002
H	0.66231885	0.24889344	-2.34939656
C	-6.12424433	0.80857385	-0.96321253

H	-4.26366008	0.66481312	1.03840170
H	-5.82512423	0.08212154	-0.21289783
H	0.51990201	2.00629672	-2.54776444
C	-6.90866778	4.72344219	3.18278852
H	-7.48043624	5.25652682	3.93910830
C	-1.34204415	0.93862350	-2.75483652
N	-0.03889664	1.26007970	-0.67628001
C	-2.06991970	-0.23727337	-2.52622786
F	-3.84121961	4.05374832	-4.33771655
H	-1.63671379	-1.02200544	-1.91253014
H	-0.46434417	2.11095194	-0.30421544
C	-6.21473764	2.63555932	2.15536577
C	-3.33598175	-0.40552003	-3.09316837
H	-6.24455735	1.54789993	2.12473729
H	-4.88943767	0.46305282	-4.31860831
C	-5.42981098	3.34151721	1.22977711
C	-3.90473498	0.59341944	-3.88892838
C	-5.38952040	4.74031139	1.29248530
O	-1.94200525	2.83691642	0.88322295
H	-4.77695762	5.29282882	0.58263770
C	-3.72189945	2.85301976	-5.00272361
C	-6.12670427	5.43090466	2.26314516
O	0.37698244	-0.93931306	-0.17198442
H	-6.08470909	6.51728131	2.30264764
C	-3.17724591	1.76224025	-4.11566499
C	-4.64621096	2.60087742	0.13977408
H	-7.55046700	2.76682242	3.84197038
H	-3.94809927	3.31287202	-0.30756039
C	-1.90605898	1.93796464	-3.55180042
C	-2.54027907	1.75576354	1.09362437
F	-5.25999772	-1.78733476	-3.49480180
C	-1.86497281	0.58842774	1.82048106
H	-1.34870195	2.85132563	-3.74293813
H	-2.33118120	-0.35696325	1.52491022
F	-4.94779402	2.56501590	-5.52682725
H	-2.07887837	0.71532792	2.89028244
F	-3.30766401	-2.78591438	-3.29638176
C	-0.33201141	0.54003903	1.62715510
C	-6.94772979	3.32312186	3.12707107
Cl	-5.19788399	-1.10641047	2.09009187

[8+Cl⁻] (ACN)

E = –8233.37 kcal mol^{-1}

H = –7950.84 kcal mol^{-1}

G = –8013.50 kcal mol^{-1}

N_{imag} = 0

C	3.42746947	4.13421785	-6.25879289
H	0.32193682	1.79056347	0.88920488
H	4.51164144	4.21239201	-6.29962547

C	1.76237498	-5.45123548	-4.48462834
C	2.62926741	5.14683821	-6.80901280
H	-1.17403597	1.74098136	-0.03805474
H	3.09221012	6.01193810	-7.27825107
F	1.08583790	-6.06888832	-5.49973258
C	1.23540232	5.03902868	-6.75363248
C	0.05419984	0.02318592	-0.25987670
H	0.60947202	5.82015544	-7.17944784
N	0.97738177	1.81903763	-3.46010449
C	0.64171873	3.92463452	-6.14748925
C	1.53971625	-1.79315521	-1.07578002
H	-0.44179346	3.84341524	-6.09837984
F	-1.95977630	-2.33361398	-6.14493260
C	1.43571610	2.90810095	-5.59930081
H	1.20028557	-2.37363931	-0.20966473
C	2.83378227	3.01945258	-5.66070589
H	1.94377280	1.73093163	-3.11087578
H	3.45941441	2.23479780	-5.24122163
H	2.63248656	-1.82747855	-1.11087491
C	2.08194781	-1.99036988	-6.76091727
H	2.40092982	-2.91362807	-7.23860204
C	0.97298181	-2.42157227	-2.34191531
N	1.16617021	-0.39729408	-0.86985475
C	1.56296524	-3.59463979	-2.82151755
F	-1.97546335	-0.63446628	-4.74272889
H	2.42421918	-4.01342882	-2.30711349
H	1.88886725	0.27590371	-1.16152035
C	0.65393896	-0.03462661	-6.71070217
C	1.06031065	-4.22374747	-3.96514985
H	-0.13820026	0.56313180	-7.15720179
H	-0.42921841	-4.18442685	-5.52789731
C	1.23973809	0.38370605	-5.50556005
C	-0.04152125	-3.69852191	-4.64114317
C	2.24990341	-0.40012339	-4.93406270
S	-1.60090373	2.38449240	-2.93860312
H	2.71730985	-0.10504560	-3.99906458
C	-1.87267276	-1.99409270	-4.82113504
C	2.66764364	-1.57975850	-5.56046144
S	-1.13270754	-1.02643188	0.33778050
H	3.44400833	-2.18454285	-5.09896140
C	-0.62985842	-2.52861793	-4.15600477
C	0.77210817	1.70977965	-4.91727096
H	0.59484588	-1.52959670	-8.26115111
H	-0.30807477	1.78815471	-5.06155062
C	-0.12506522	-1.88517621	-3.02142989
C	0.03941162	2.17981328	-2.57595991
F	3.01866828	-5.15362324	-4.97649719
C	0.55398240	2.42223114	-1.16172435
H	-0.58449860	-0.96638396	-2.67654938
H	0.31713860	3.46088880	-0.90548290
F	-3.01809204	-2.49921061	-4.22265572

H	1.64441130	2.33051922	-1.13636444
F	1.95802866	-6.39457662	-3.50226468
C	-0.10493351	1.52800446	-0.08859918
C	1.06913981	-1.21272540	-7.33522043
Cl	3.83672703	1.12646405	-1.99580938

[8+Cl⁻]' (ACN)

E = –8232.13 kcal mol^{-1}

H = –7949.56 kcal mol^{-1}

G = –8011.94 kcal mol^{-1}

N_{imag} = 0

C	-5.64319505	-0.61596002	-1.63754176
H	0.30649531	1.27779613	1.66721929
H	-5.29222354	-1.63431541	-1.78347178
C	-4.24659592	-1.12417809	-5.00513525
C	-6.82049503	-0.18329548	-2.25549871
H	-0.10382515	-0.25436546	2.46298655
H	-7.39328015	-0.86260387	-2.88211410
F	-4.36468834	-2.31779318	-4.33629235
C	-7.24896904	1.13574938	-2.06926975
C	-0.76063623	-0.13067702	0.43443655
H	-8.15882095	1.48808217	-2.55029937
N	-3.65381098	1.81786822	1.14432064
C	-6.51340516	2.00614845	-1.25933432
C	-0.41416270	0.15352777	-2.01999467
H	-6.86020734	3.02513715	-1.11320930
F	-3.21244812	4.25877514	-4.67973735
C	-5.33294938	1.57613833	-0.63456235
H	-0.28740406	-0.93143014	-2.05606093
C	-4.89986893	0.25822840	-0.84040613
H	-4.09839705	1.02607479	1.64787813
H	-3.97984559	-0.09604350	-0.38588342
H	0.42674754	0.62061160	-2.54319034
C	-6.90997018	5.53288986	2.24487632
H	-7.51507375	6.27989157	2.75390448
C	-1.71064875	0.53612194	-2.71981693
N	-0.32189003	0.57096164	-0.62032683
C	-2.40884519	-0.42688572	-3.45071689
F	-3.32485726	4.40246750	-2.48706467
H	-2.06571831	-1.45721587	-3.44431460
H	0.10104321	1.47612886	-0.42900120
C	-6.20114493	3.22651482	1.98167188
C	-3.54717034	-0.07083322	-4.18403205
H	-6.25220957	2.18324461	2.28793552
H	-4.88453046	1.52029184	-4.76317267
C	-5.34723651	3.60117176	0.93257563
C	-4.00165060	1.24682715	-4.19800881
C	-5.27896006	4.94592332	0.54917803
S	-1.58010632	3.49737871	0.85421264

H	-4.61212797	5.23878093	-0.25774457
C	-3.73718165	3.64917632	-3.55344203
C	-6.06007967	5.91001415	1.19954883
S	-1.59878095	-1.58733255	0.30188743
H	-6.00008286	6.95194475	0.89270108
C	-3.30439816	2.20731705	-3.45837275
C	-4.50765061	2.55648123	0.20262334
H	-7.63377949	3.88977600	3.44971214
H	-3.81621256	3.09322295	-0.45550852
C	-2.17024708	1.85986696	-2.71836790
C	-2.40492001	2.14479325	1.46649526
F	-5.50403919	-0.75646783	-5.39282237
C	-1.73088205	1.16393011	2.41279134
H	-1.64923838	2.61667345	-2.13953773
H	-2.44105312	0.38175359	2.69996546
F	-5.09568680	3.79158440	-3.64422834
H	-1.41831840	1.69686347	3.31721812
F	-3.54811352	-1.40839564	-6.16482059
C	-0.47171176	0.51576458	1.77929116
C	-6.97618776	4.18807772	2.63589698
Cl	-5.17884732	-0.36218247	2.93541640

[8+I⁻] (ACN)

E = –8209.64 kcal mol^{-1}

H = –7927.58 kcal mol^{-1}

G = –7990.85 kcal mol^{-1}

N_{imag} = 0

C	3.17734282	4.18439178	-6.41280850
H	0.50404206	1.89831862	0.88955626
H	4.24449425	4.35333922	-6.53843699
C	1.67006093	-5.20882798	-4.66987849
C	2.25703709	5.14259873	-6.86022204
H	-1.04212961	1.91124918	0.04478275
H	2.60835452	6.05641824	-7.33396704
F	1.42175685	-5.34348850	-6.00849971
C	0.88552771	4.91735376	-6.69854562
C	0.02665382	0.08718744	-0.12913726
H	0.16608526	5.65528909	-7.04652318
N	1.08059758	1.66753741	-3.48989528
C	0.43501106	3.74016079	-6.08792859
C	1.33374008	-1.87553000	-0.93206982
H	-0.63039476	3.56629323	-5.95632364
F	-2.66737927	-2.69728936	-5.54109358
C	1.35150124	2.77846241	-5.64170846
H	0.85344924	-2.42093706	-0.11162591
C	2.72613708	3.00709635	-5.81020533
H	2.05621120	1.68647800	-3.17135932

H	3.44336799	2.26097600	-5.47450002
H	2.41430875	-2.03426854	-0.87303046
C	2.55958790	-2.01862439	-6.79047725
H	2.98050059	-2.89761865	-7.27246157
C	0.81089387	-2.39955142	-2.26194814
N	1.09350297	-0.44910702	-0.73051568
C	1.44588445	-3.49618645	-2.85249840
F	-1.73972252	-0.70629996	-5.51248847
H	2.32304407	-3.92950885	-2.38017220
H	1.86866165	0.15626755	-1.02793888
C	1.09443153	-0.08967162	-6.84764152
C	0.97248175	-4.02063020	-4.05890679
H	0.38034426	0.53317584	-7.38242780
H	-0.51941956	-3.88830845	-5.61349912
C	1.45942342	0.24836386	-5.53331681
C	-0.15214011	-3.47626117	-4.68155272
C	2.37393672	-0.56330025	-4.85634289
S	-1.51913518	2.01540221	-2.91450525
H	2.67704813	-0.32905863	-3.84126317
C	-2.02265341	-1.79850004	-4.72611634
C	2.92555424	-1.68691047	-5.48467920
S	-1.23845837	-0.82851088	0.52061892
H	3.63602987	-2.30578895	-4.94282199
C	-0.79165679	-2.38966605	-4.08114390
C	0.85190038	1.51423808	-4.94033844
H	1.33284130	-1.46814089	-8.48510310
H	-0.23452174	1.46242669	-5.05078883
C	-0.30806017	-1.83991557	-2.88898706
C	0.13748329	1.98759147	-2.59091849
F	3.03384442	-5.14889671	-4.52078095
C	0.67312848	2.35739597	-1.21333106
H	-0.80823240	-0.98241777	-2.45296163
H	0.48289489	3.42794940	-1.06981308
F	-2.94086133	-1.37717797	-3.79827376
H	1.76125251	2.23152768	-1.18800274
F	1.27532455	-6.39554180	-4.08030030
C	0.00142966	1.60708007	-0.04299559
C	1.63408939	-1.21548215	-7.47085788
I	4.31808001	0.91139707	-1.76897785

[8+I⁻]' (ACN)

E = –8209.98 kcal mol^{-1}

H = –7927.60 kcal mol^{-1}

G = –7991.06 kcal mol^{-1}

N_{imag} = 0

C	-5.65033856	-0.61337804	-1.63256596
H	0.31371362	1.30463957	1.65679491
H	-5.30270736	-1.63308833	-1.77632321
C	-4.25389083	-1.11977611	-4.99529368

C	-6.82958446	-0.18007069	-2.24546532
H	-0.08663705	-0.23040225	2.45255589
H	-7.40723324	-0.85992651	-2.86704201
F	-4.36891140	-2.31160444	-4.32291251
C	-7.25443273	1.14053355	-2.06080444
C	-0.75672933	-0.10659779	0.42801063
H	-8.16585704	1.49357505	-2.53830640
N	-3.64334114	1.82585100	1.13606588
C	-6.51322772	2.01110459	-1.25660596
C	-0.41273261	0.16994655	-2.02717959
H	-6.85722303	3.03110958	-1.11086909
F	-3.19683911	4.27338595	-4.67510865
C	-5.33081860	1.57981410	-0.63633115
H	-0.28615224	-0.91519450	-2.06081955
C	-4.90111811	0.26115754	-0.84171865
H	-4.07250919	1.01907938	1.61586400
H	-3.97937339	-0.09561179	-0.39285569
H	0.42637048	0.63576103	-2.55426923
C	-6.89123142	5.53901540	2.25491752
H	-7.49170348	6.28647060	2.76870655
C	-1.71159141	0.54999295	-2.72389167
N	-0.31602761	0.59121784	-0.62890912
C	-2.41222818	-0.41609589	-3.44853888
F	-3.34868722	4.40958959	-2.48471727
H	-2.06869162	-1.44625645	-3.43922300
H	0.12050917	1.49016982	-0.44047830
C	-6.22601586	3.22541982	1.95005819
C	-3.55297830	-0.06330935	-4.17937627
H	-6.30913838	2.17637132	2.22892076
H	-4.89352895	1.52499032	-4.75908662
C	-5.34051948	3.60626921	0.93005768
C	-4.00823310	1.25413245	-4.19656164
C	-5.23561761	4.95793664	0.58097505
S	-1.58727589	3.52561700	0.87094843
H	-4.54495227	5.25547521	-0.20364052
C	-3.74158724	3.65926887	-3.56098919
C	-6.01062613	5.92262552	1.23777629
S	-1.60571534	-1.55665874	0.30080400
H	-5.92237346	6.96999403	0.95749658
C	-3.30899161	2.21757647	-3.46289167
C	-4.50388241	2.56155204	0.19654319
H	-7.67697118	3.88296932	3.40230437
H	-3.81413289	3.09797885	-0.46334587
C	-2.17129461	1.87355743	-2.72634544
C	-2.39830945	2.16270103	1.47037871
F	-5.51268260	-0.75439173	-5.38054322
C	-1.71908900	1.18171374	2.41194563
H	-1.64750711	2.63303767	-2.15344391
H	-2.42351974	0.39402908	2.69992654
F	-5.09812730	3.80094174	-3.67529210
H	-1.40647617	1.71297299	3.31710727

```
F     -3.55824837    -1.40654447    -6.15614545
C     -0.46074951     0.53918049     1.77162362
C     -6.99477416     4.18691919     2.61120085
I     -5.39654781    -0.67454226     3.09555261
```

Table S9. Gas phase absolute energies, enthalpies, and Gibbs free energies (in kcal mol^{-1}) for the receptors 3 and 8, anions Cl^- and I^-, and complexes of the stationary points given in Table S4 - S6, computed at COSMO(ACN)-ZORA-BLYP-D3(BJ)/TZ2P.

System	*E* (kcal mol^{-1})	*H* (kcal mol^{-1})	*G* (kcal mol^{-1})
Cl^-	–83.82	–82.34	–93.25
I^-	–74.97	–73.49	–85.54
**3**-I	–8210.98	–7926.81	–7985.20
**3**-II	–8210.11	–7925.90	–7984.22
**3**-III	–8210.10	–7925.97	–7984.13
**3**-IV	–8207.76	–7923.61	–7982.03
**8**-I	–8060.12	–7778.18	–7837.50
**8**-II	–8058.61	–7776.84	–7835.77
**8**-III	–8056.87	–7774.99	–7835.32
**8**-IV	–8057.03	–7775.15	–7835.56
[**3**+Cl^-]	–8337.92	–8052.95	–8114.58
[**3**+Cl^-]’	–8330.65	–8045.54	–8107.06
[**3**+I^-]	–8319.59	–8035.25	–8096.28
[**8**+Cl^-]	–8191.35	–7908.79	–7969.74
[**8**+Cl^-]’	–8178.43	–7895.79	–7957.95
[**8**+I^-]	–8169.92	–7887.52	–7950.70

S12 NMR spectra of synthesized compounds

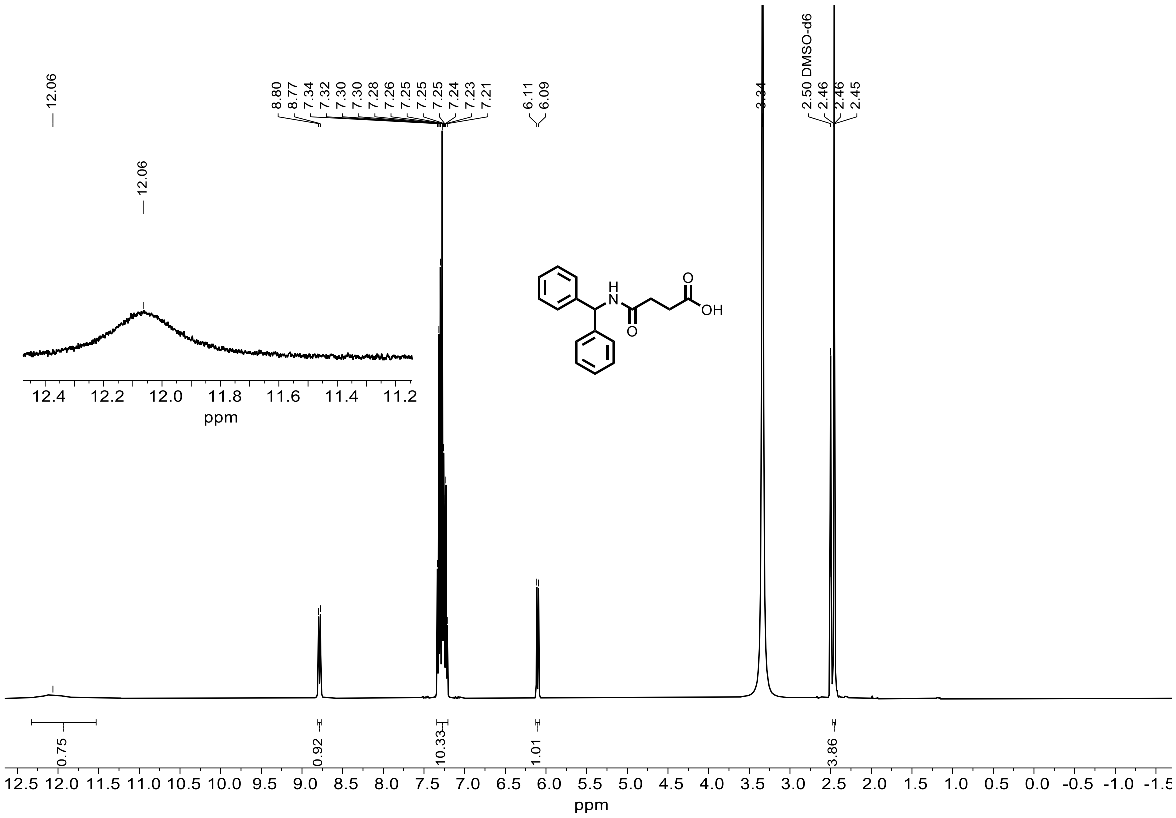

Figure S41. ^{1}H NMR spectrum (400 MHz, T = 298 K) of **12** in dimethyl sulfoxide-d_6.

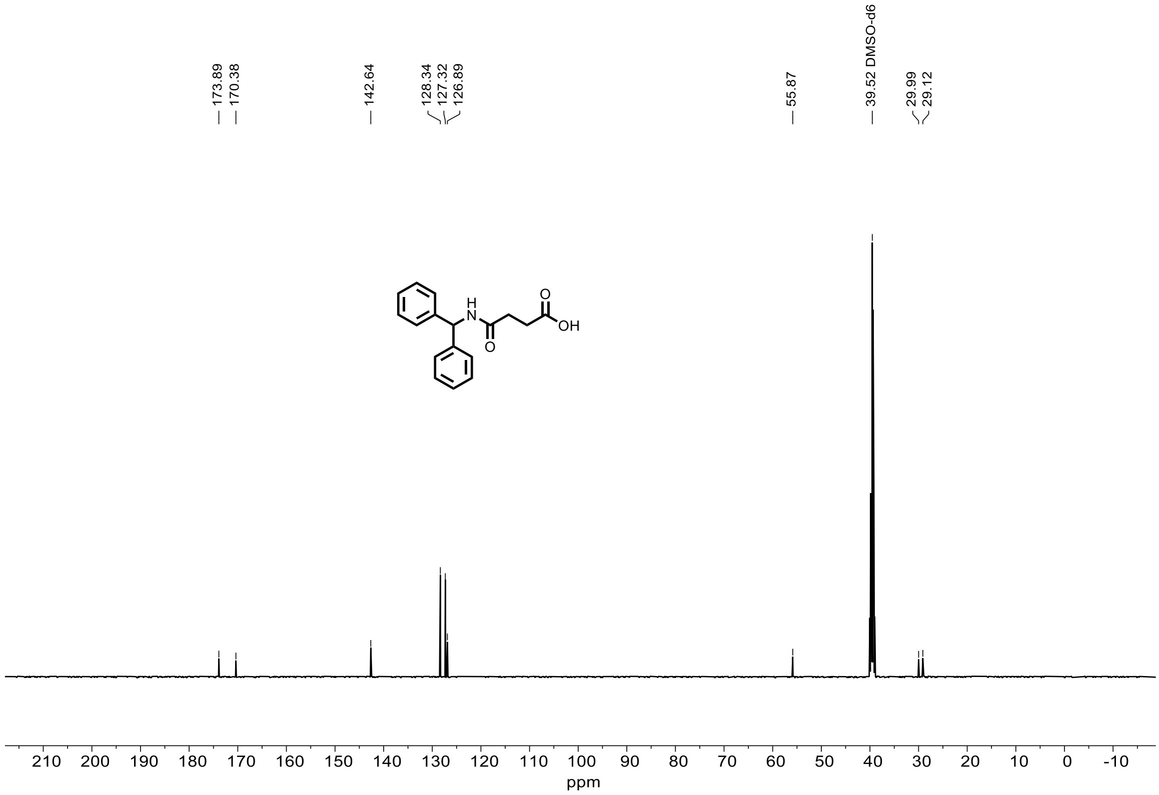

Figure S42. ^{13}C NMR spectrum (125 MHz, T = 298 K) of **12** in dimethyl sulfoxide-d_6.

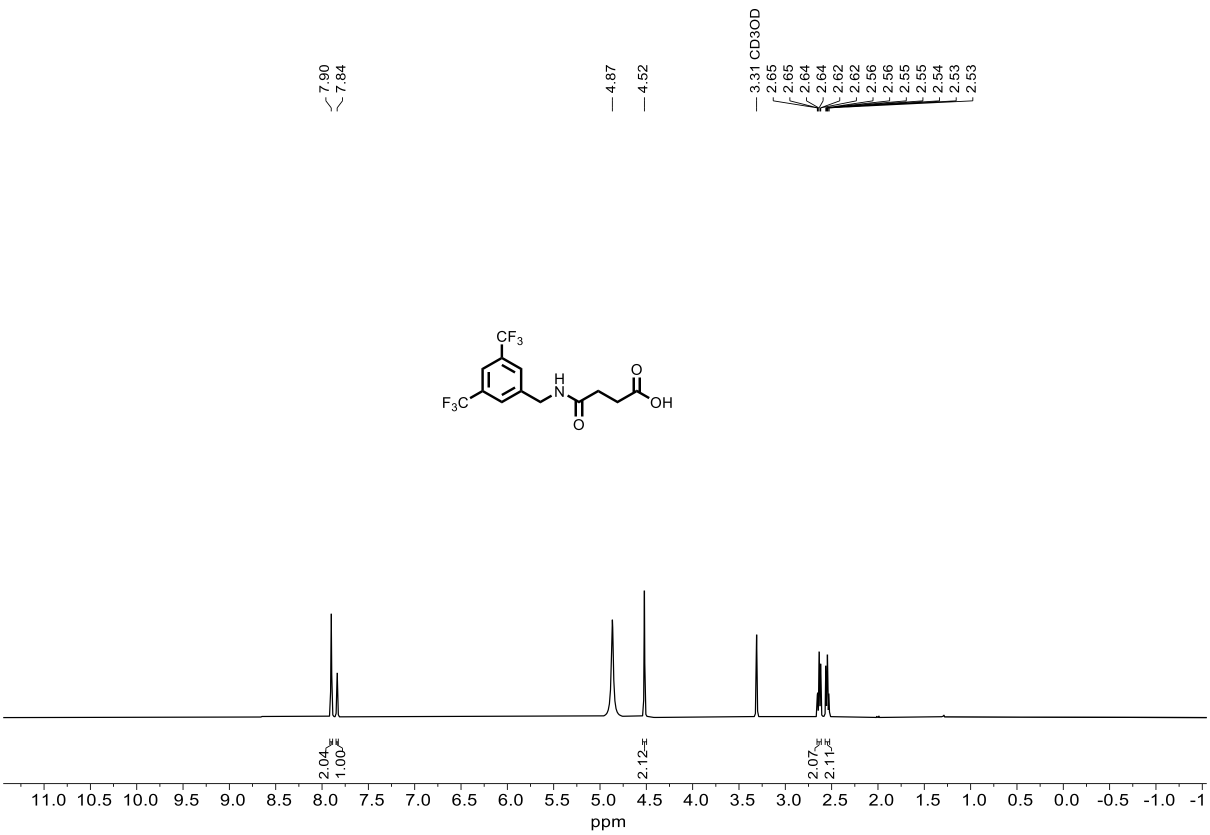

Figure S43. ^{1}H NMR spectrum (400 MHz, T = 298 K) of **14** in methanol-d_4.

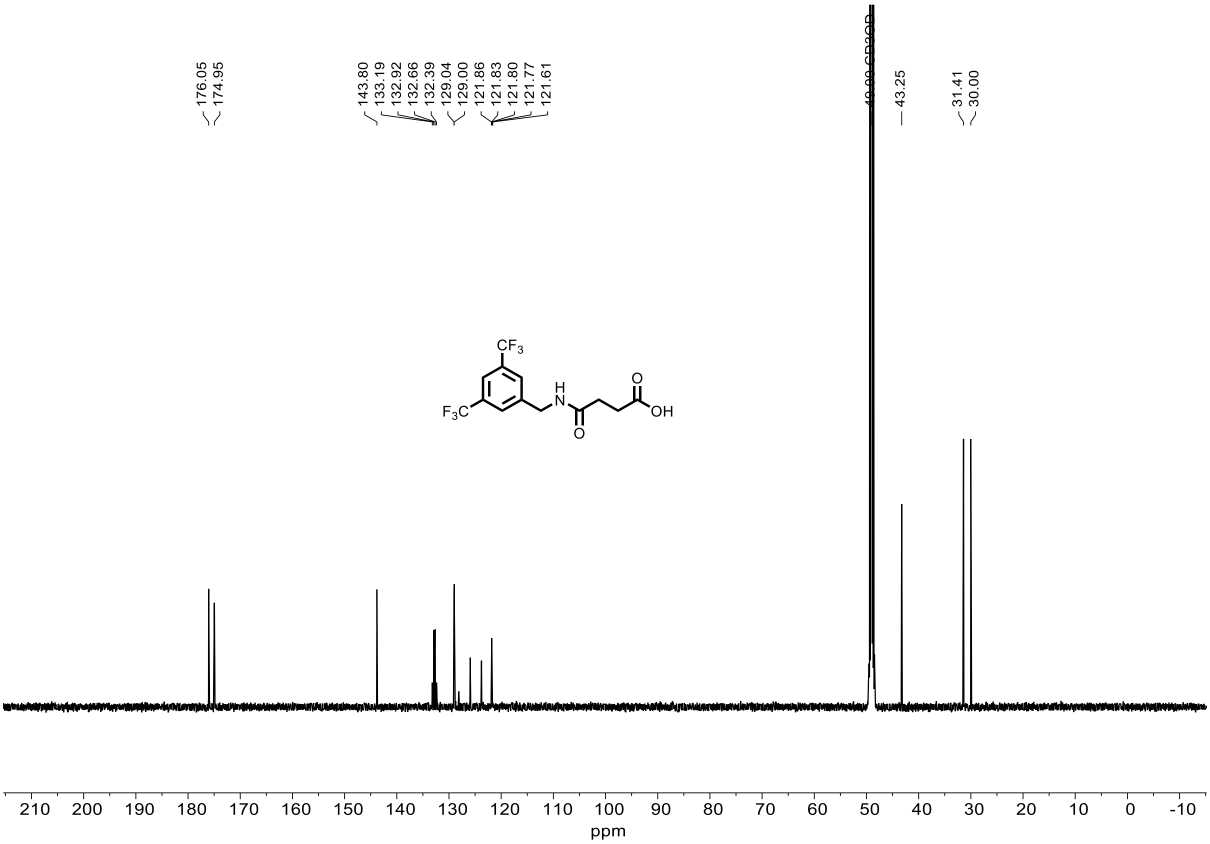

Figure S44. ^{13}C NMR spectrum (125 MHz, T = 298 K) of **14** in methanol-d_4.

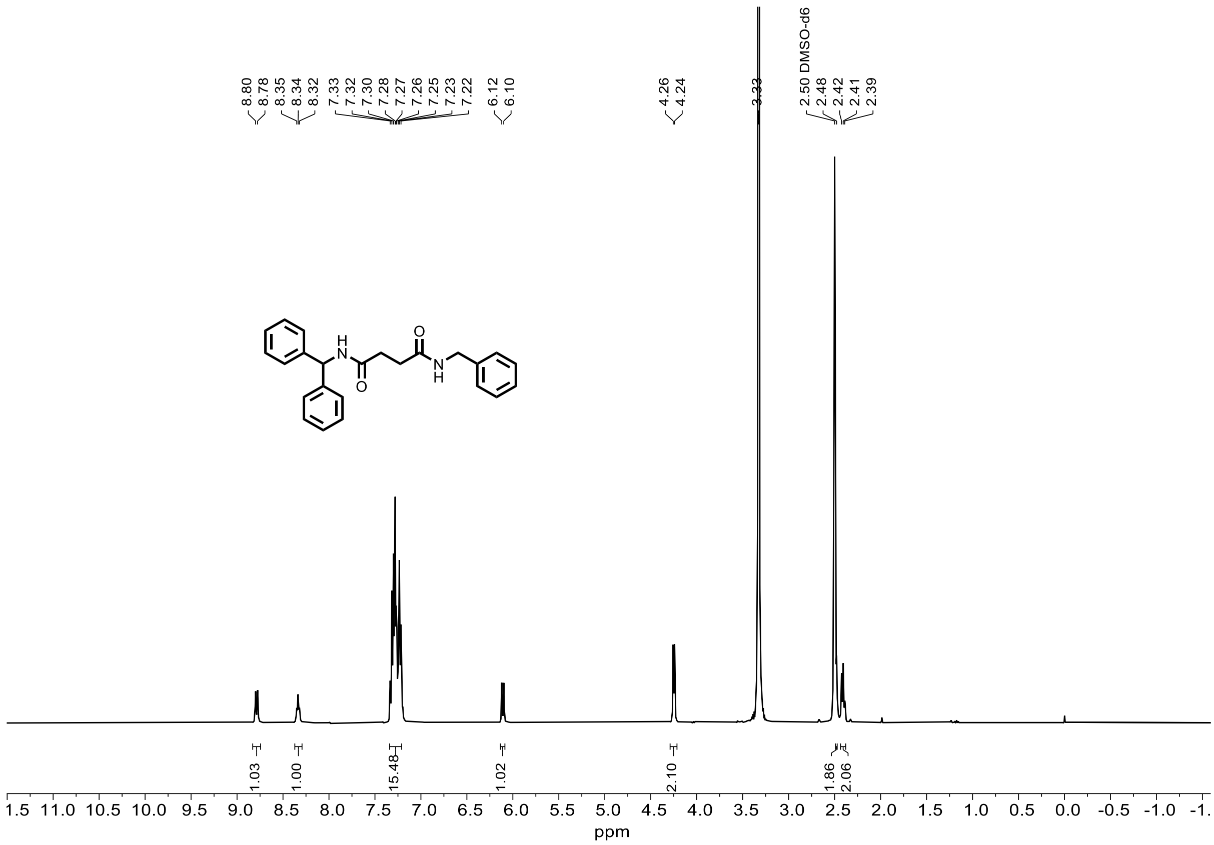

Figure S45. ^{1}H NMR spectrum (400 MHz, T = 298 K) of **1** in dimethyl sulfoxide-d_6.

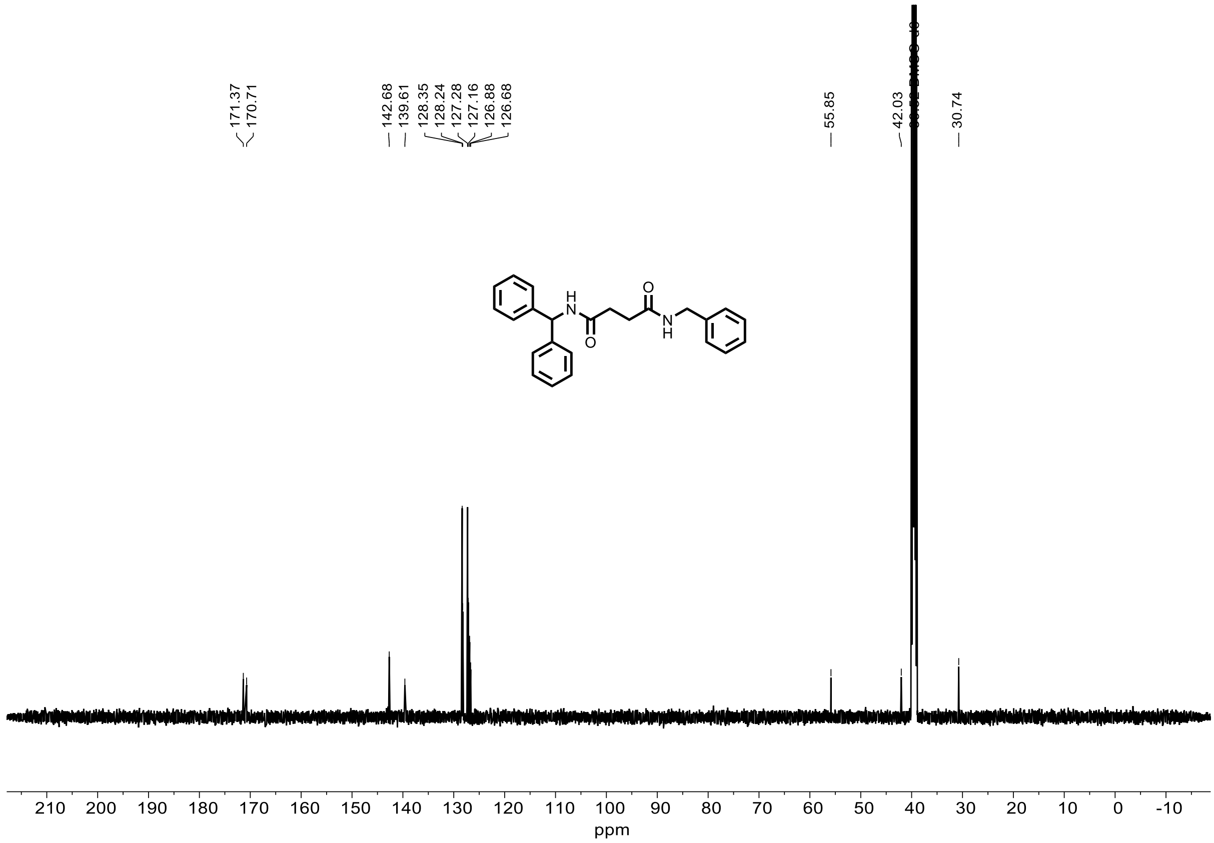

Figure S46. ^{13}C NMR spectrum (125 MHz, T = 298 K) of **1** in dimethyl sulfoxide-d_6.

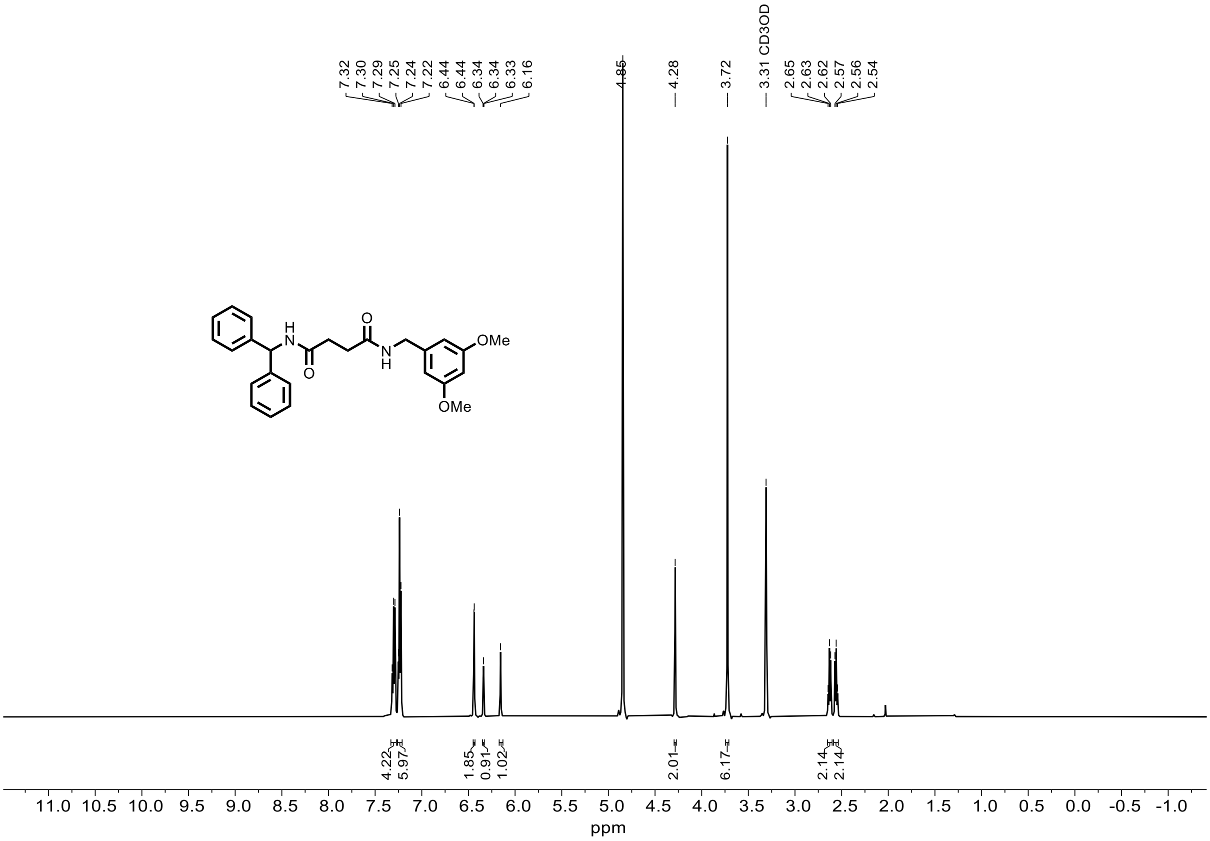

Figure S47. ^{1}H NMR spectrum (500 MHz, T = 298 K) of **2** in methanol-d_4.

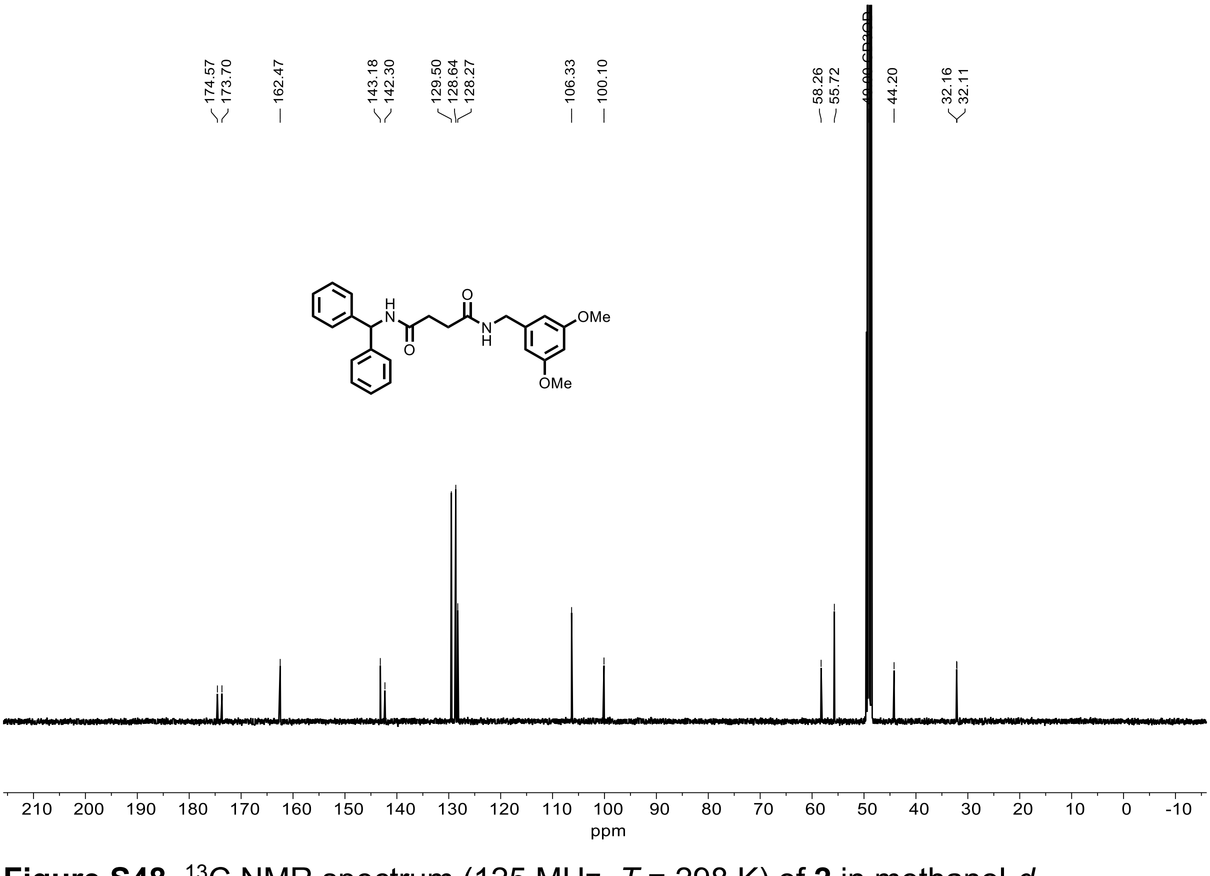

Figure S48. ^{13}C NMR spectrum (125 MHz, T = 298 K) of **2** in methanol-d_4.

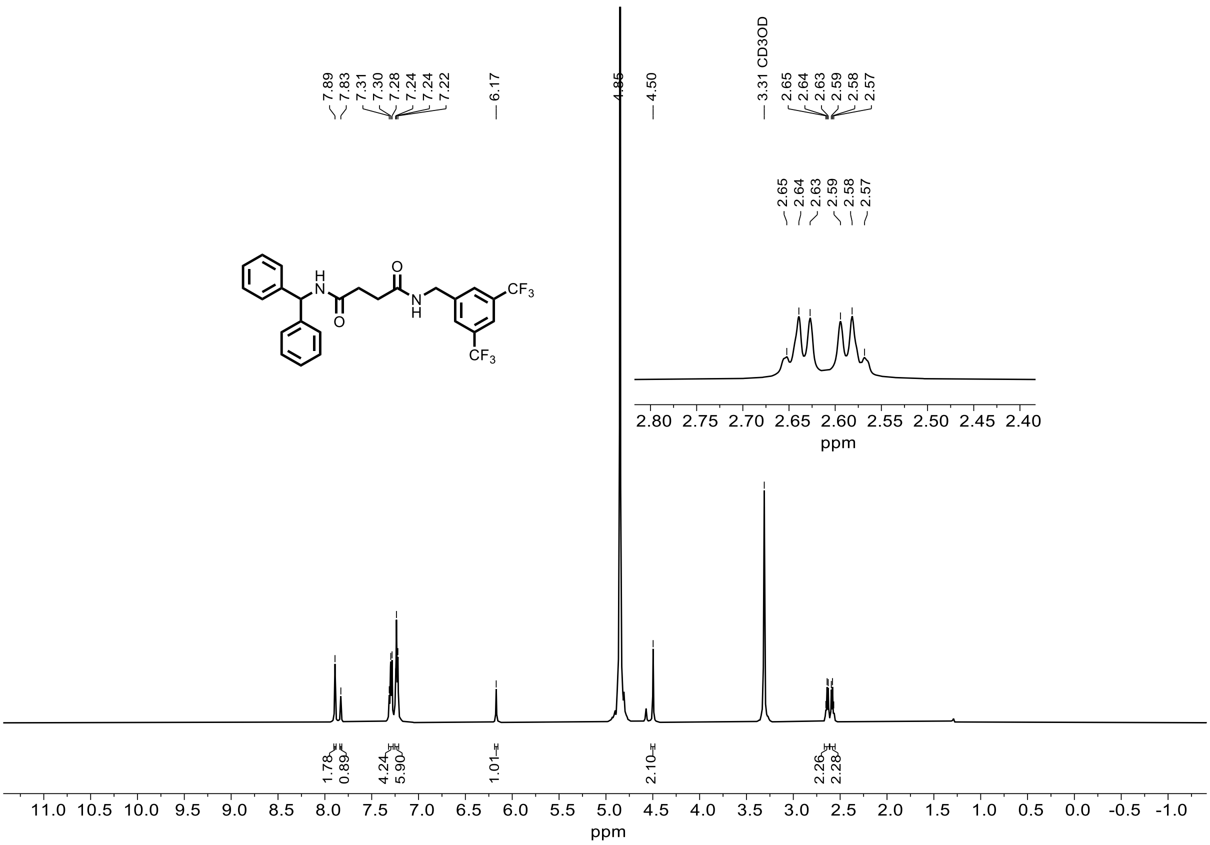

Figure S49. ^{1}H NMR spectrum (500 MHz, T = 298 K) of **3** in methanol-d_4.

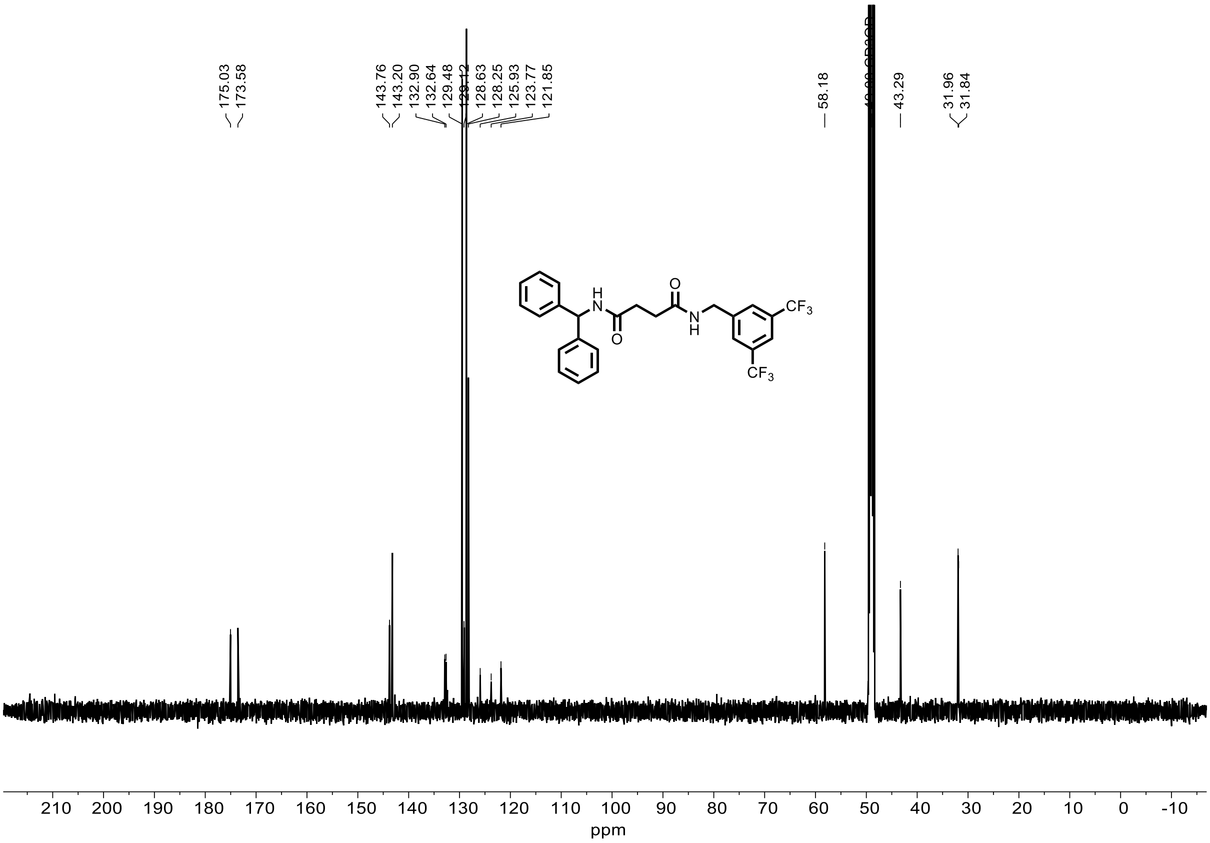

Figure S50. ^{13}C NMR spectrum (125 MHz, T = 298 K) of **3** in methanol-d_4.

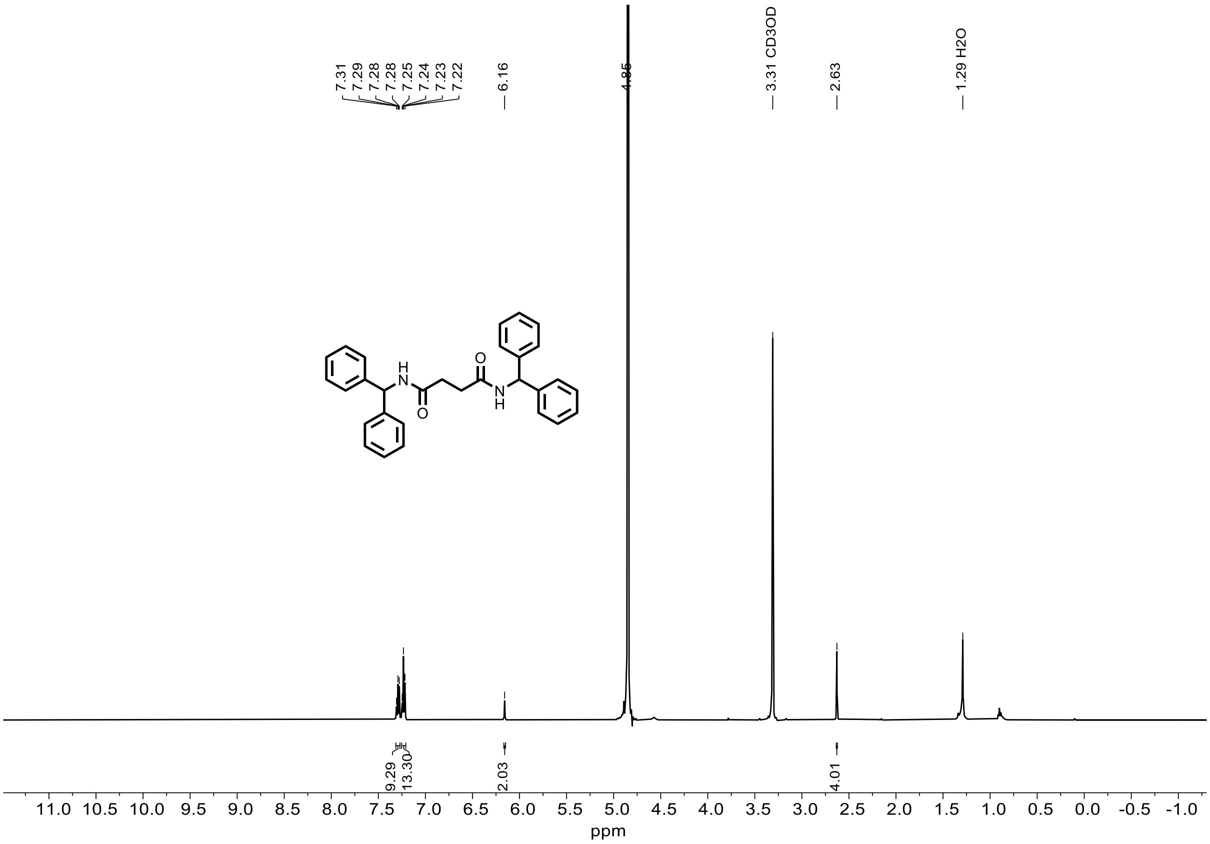

Figure S51. ^{1}H NMR spectrum (500 MHz, T = 298 K) of **4** in methanol-d_4.

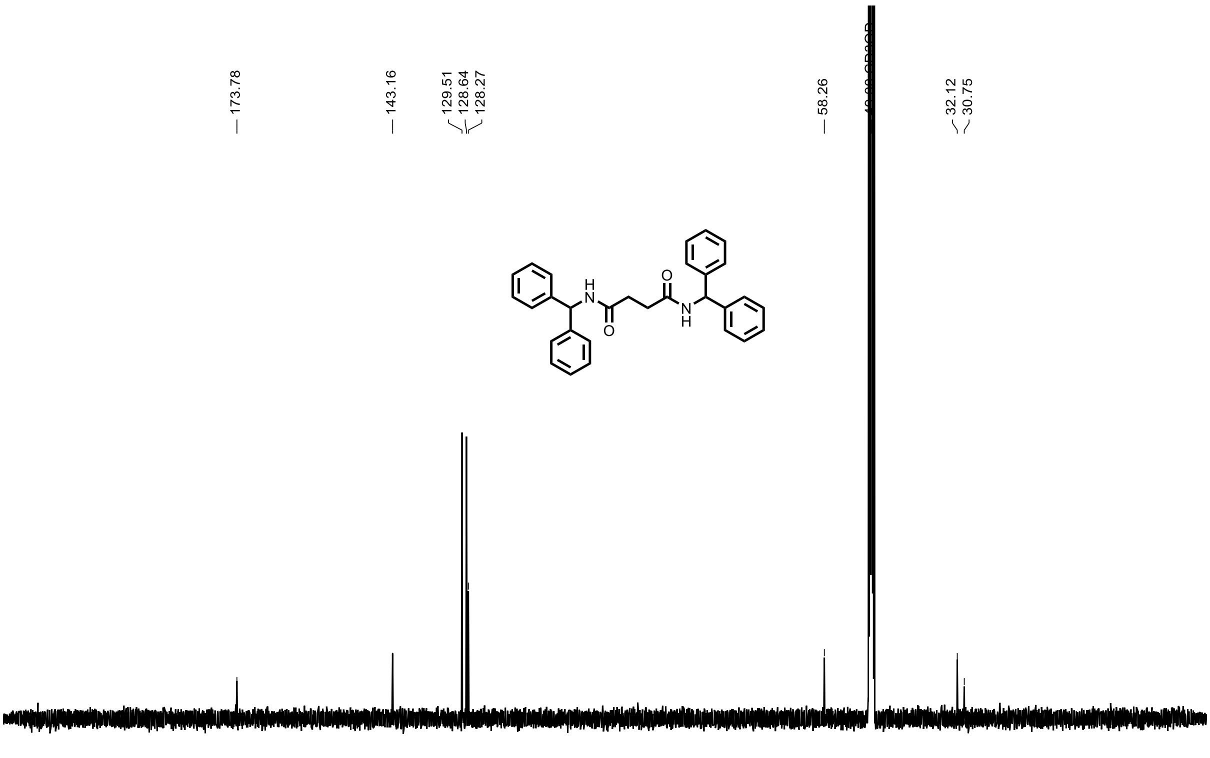

Figure S52. ^{13}C NMR spectrum (125 MHz, T = 298 K) of **4** in methanol-d_4.

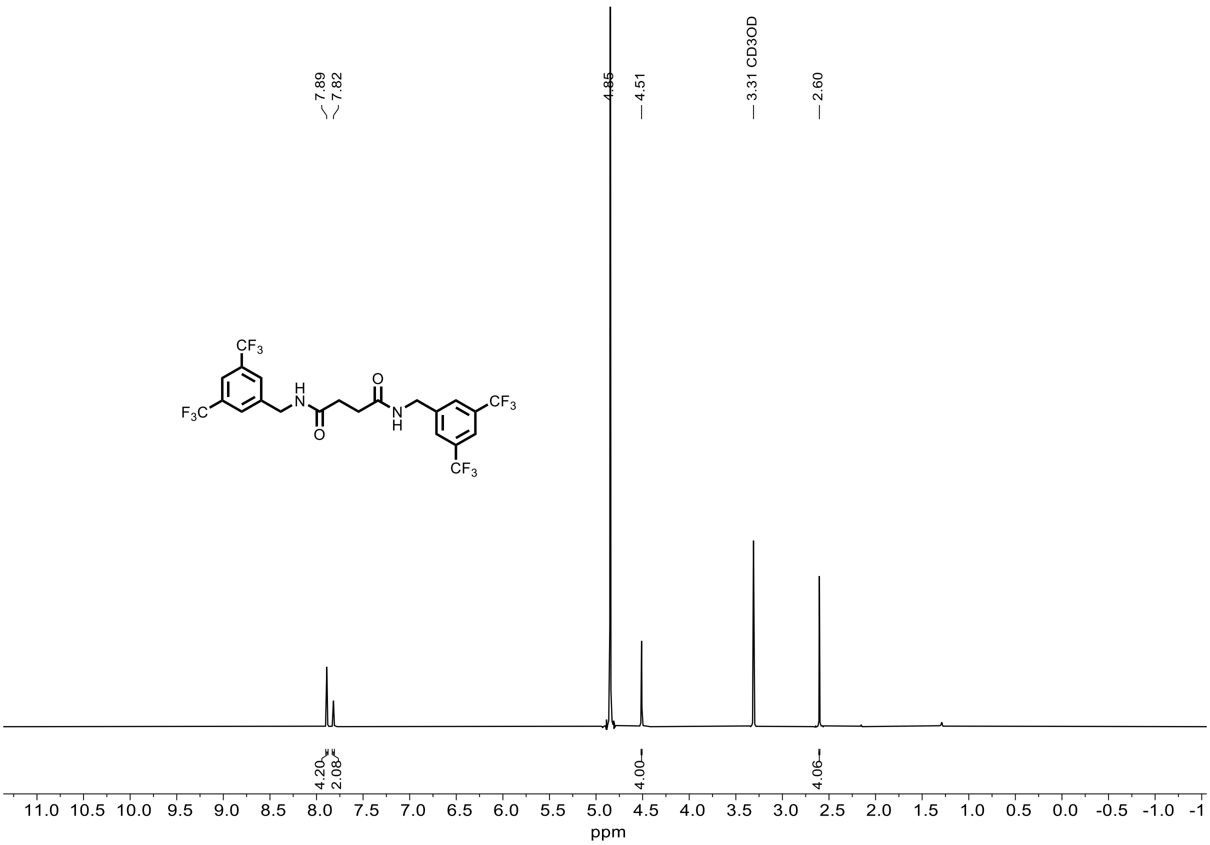

Figure S53. ^{1}H NMR spectrum (500 MHz, T = 298 K) of **5** in methanol-d_4.

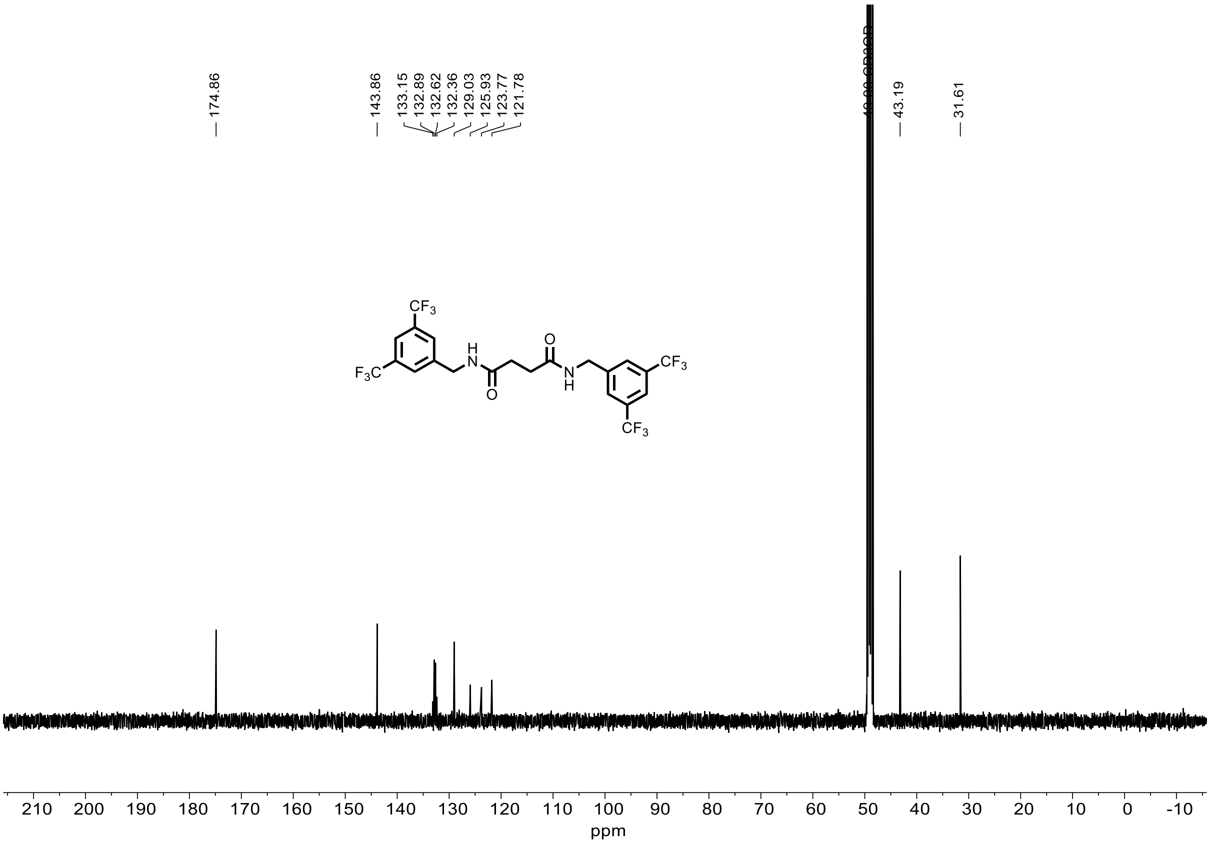

Figure S54. ^{1}H NMR spectrum (125 MHz, T = 298 K) of **5** in methanol-d_4.

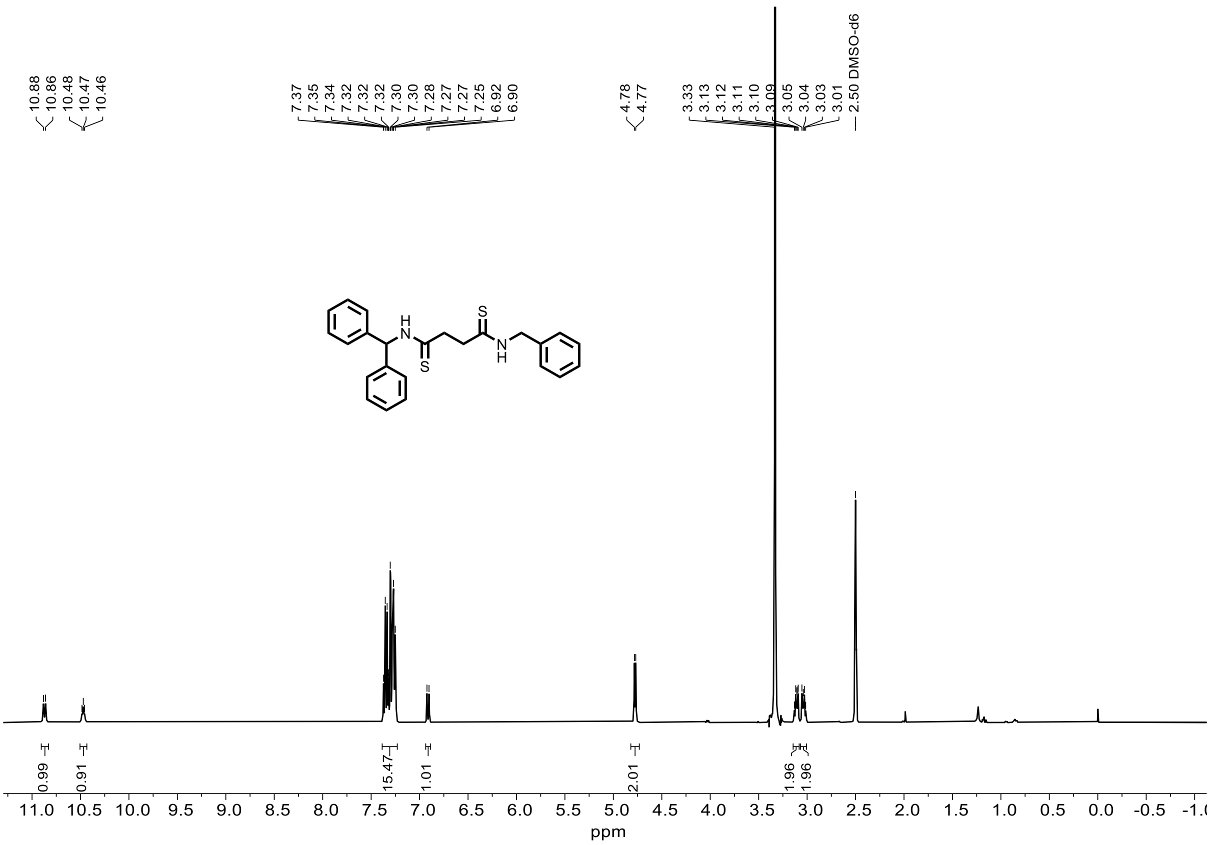

Figure S55. ^{1}H NMR spectrum (400 MHz, T = 298 K) of **6** in dimethyl sulfoxide-d_6.

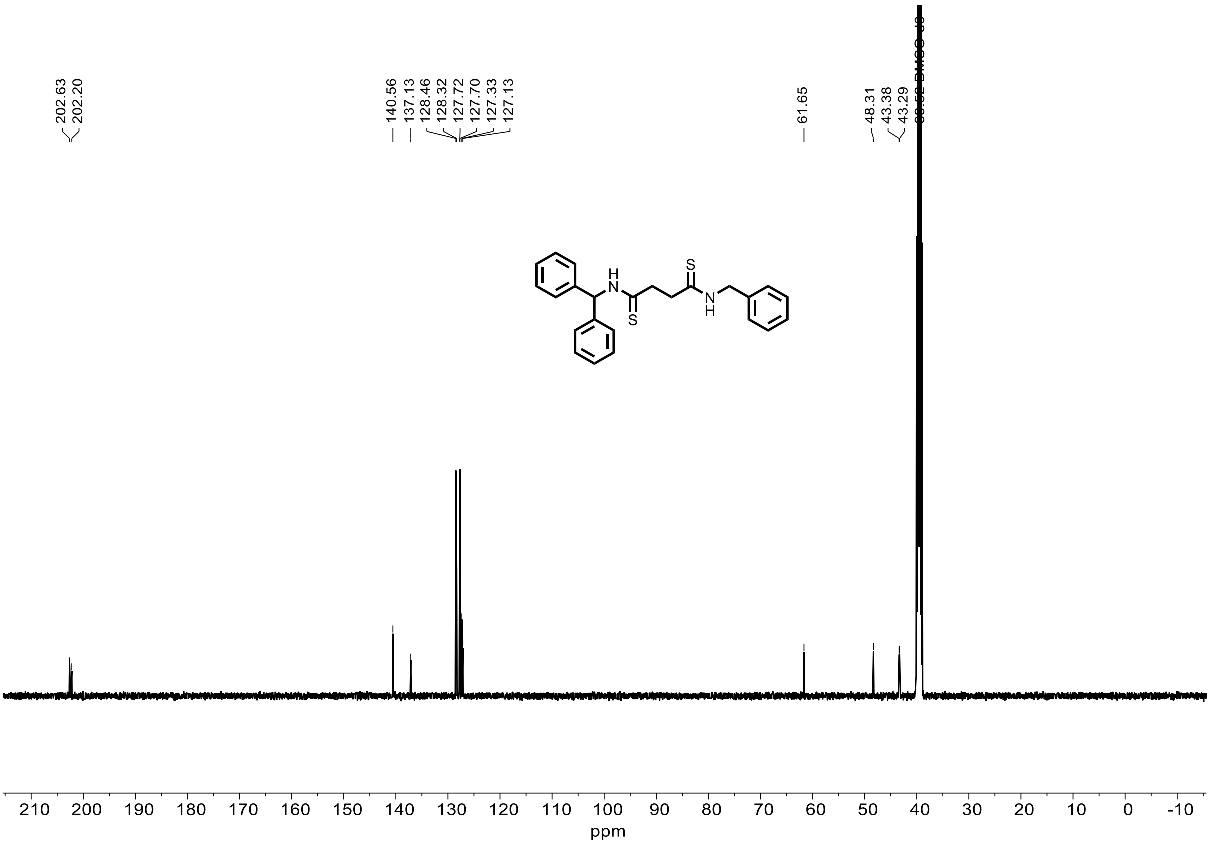

Figure S56. ^{13}C NMR spectrum (125 MHz, T = 298 K) of **6** in dimethyl sulfoxide-d_6.

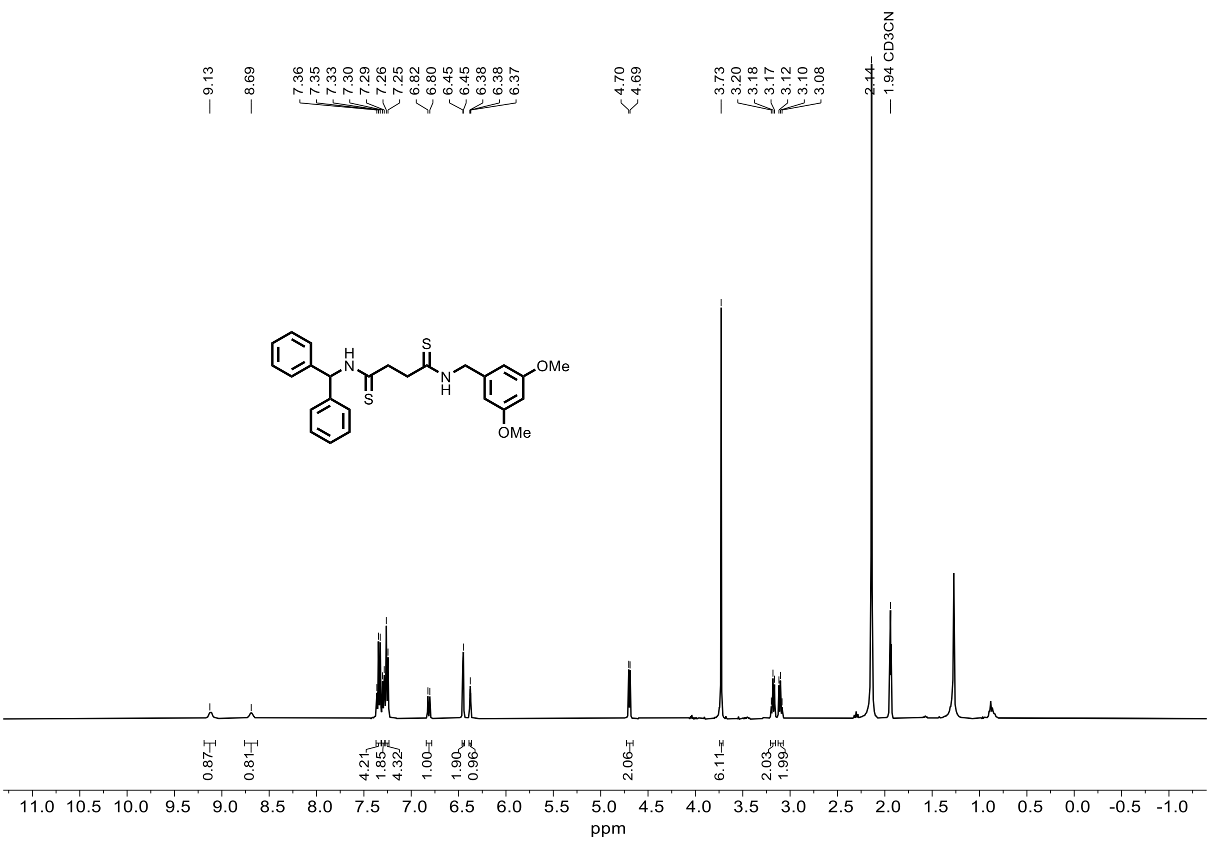

Figure S57. ^{1}H NMR spectrum (400 MHz, T = 298 K) of **7** in acetonitrile-d_3.

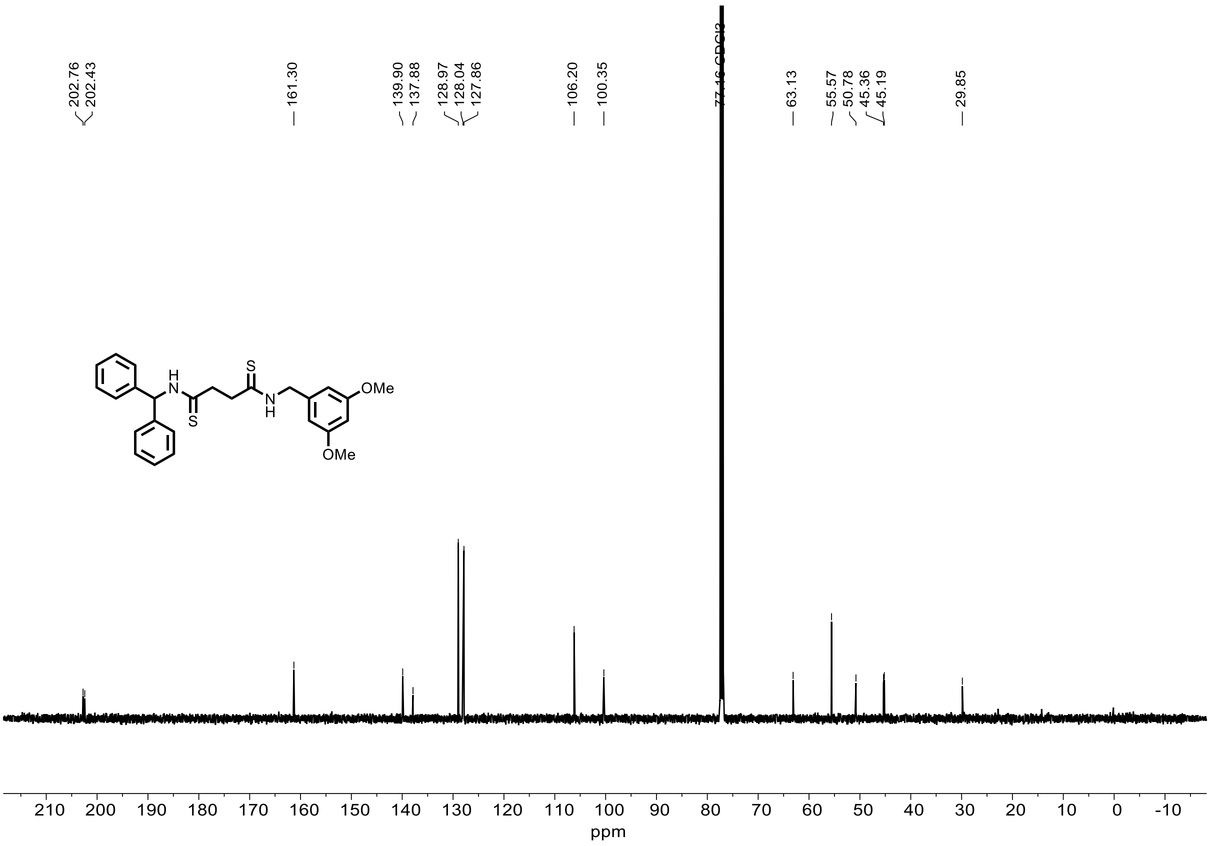

Figure S58. ^{13}C NMR spectrum (125 MHz, T = 298 K) of **7** in chloroform-d.

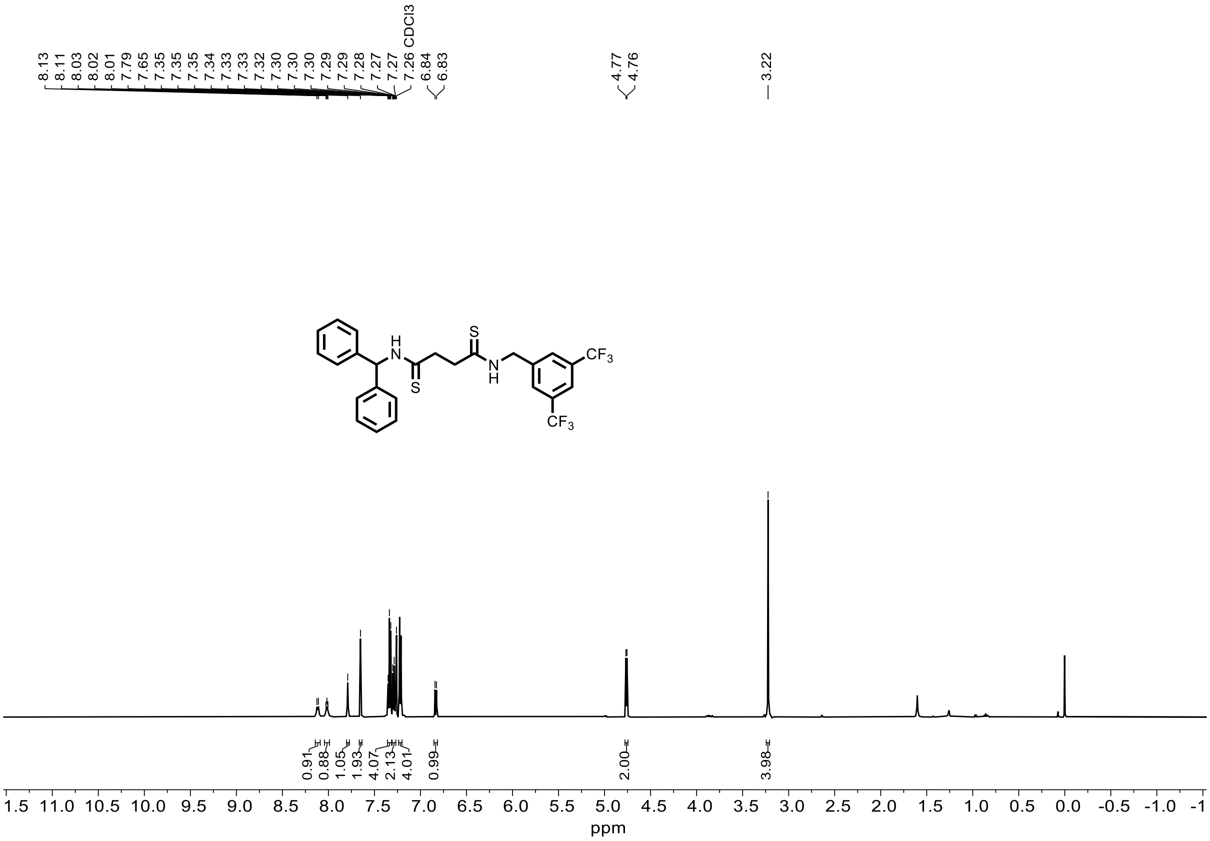

Figure S59. ^{1}H NMR spectrum (500 MHz, T = 298 K) of **8** in chloroform-d.

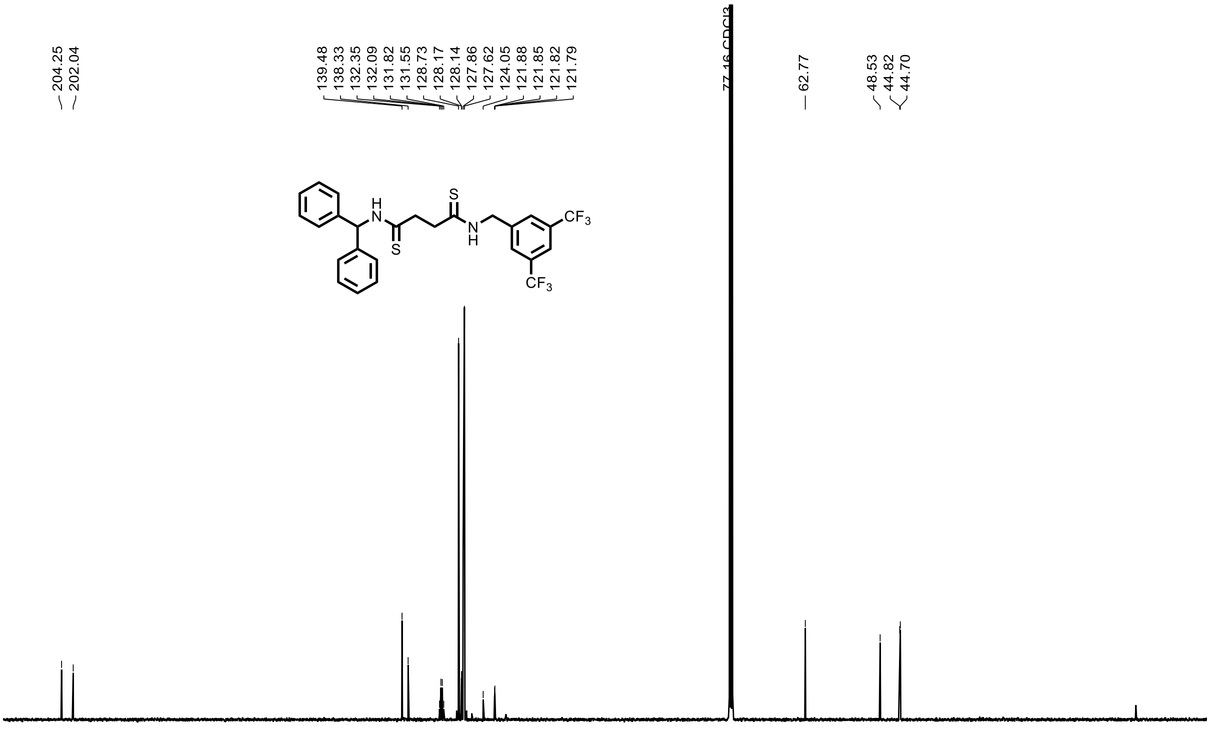

Figure S60. ^{13}C NMR spectrum (125 MHz, T = 298 K) of **8** in chloroform-d.

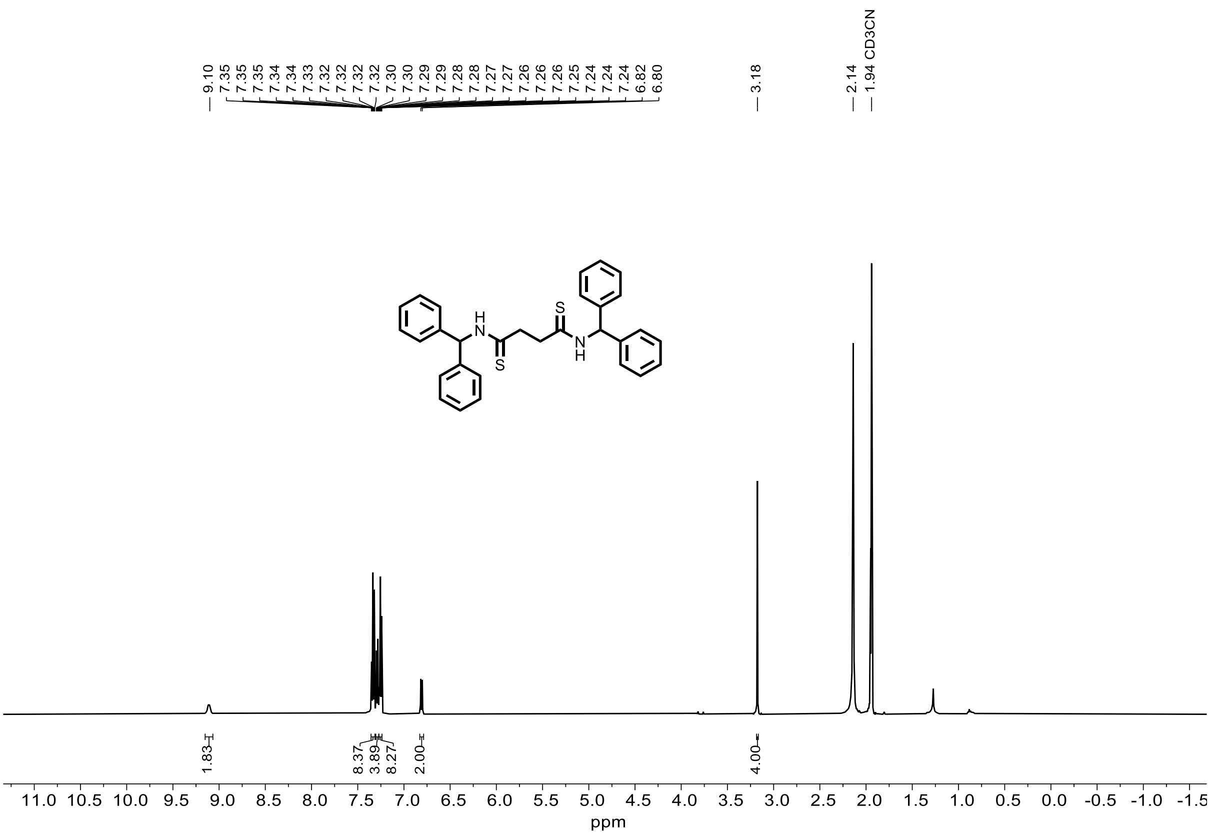

Figure S61. ^{1}H NMR spectrum (500 MHz, T = 298 K) of **9** in acetonitrile-d_3.

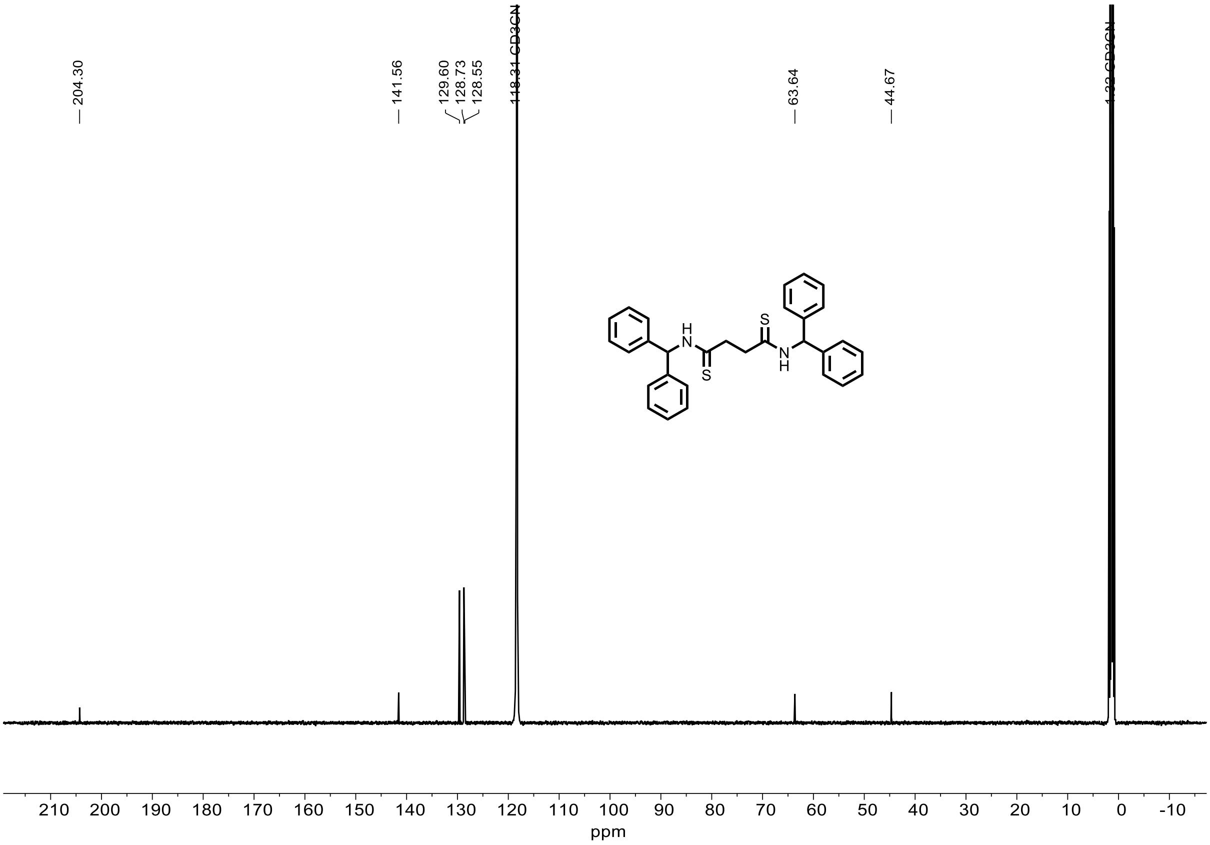

Figure S62. ^{13}C NMR spectrum (125 MHz, T = 298 K) of **9** in acetonitrile-d_3.

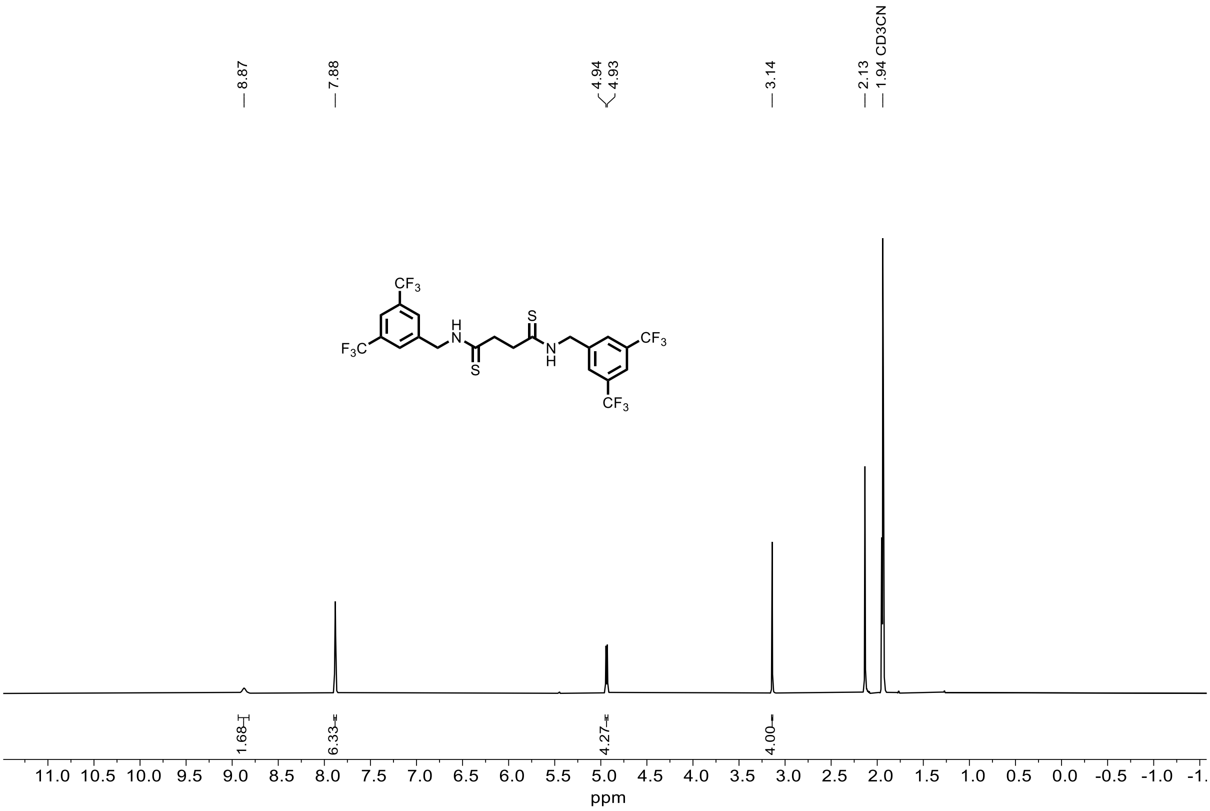

Figure S63. ^{1}H NMR spectrum (400 MHz, T = 298 K) of **10** in acetonitrile-d_3.

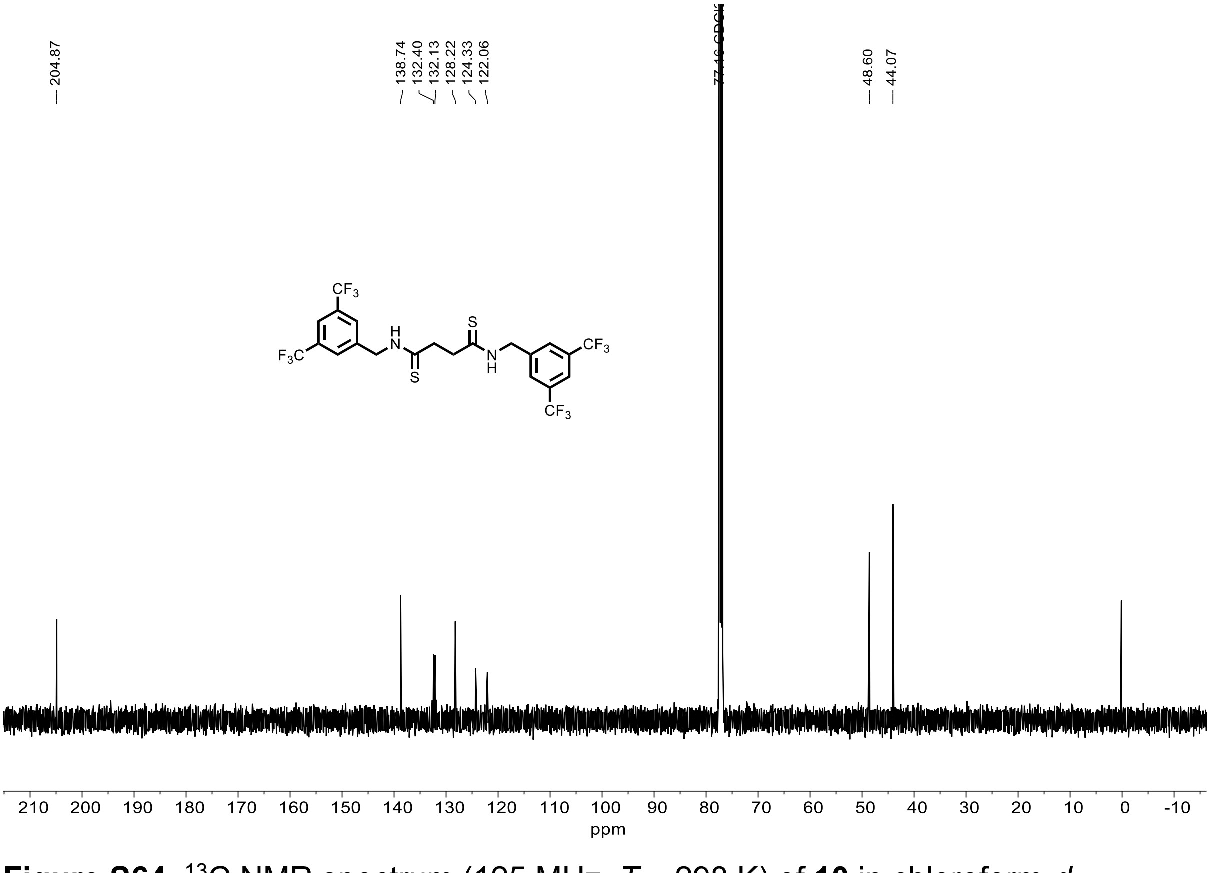

Figure S64. ^{13}C NMR spectrum (125 MHz, T = 298 K) of **10** in chloroform-d.

S13 Mass Spectra

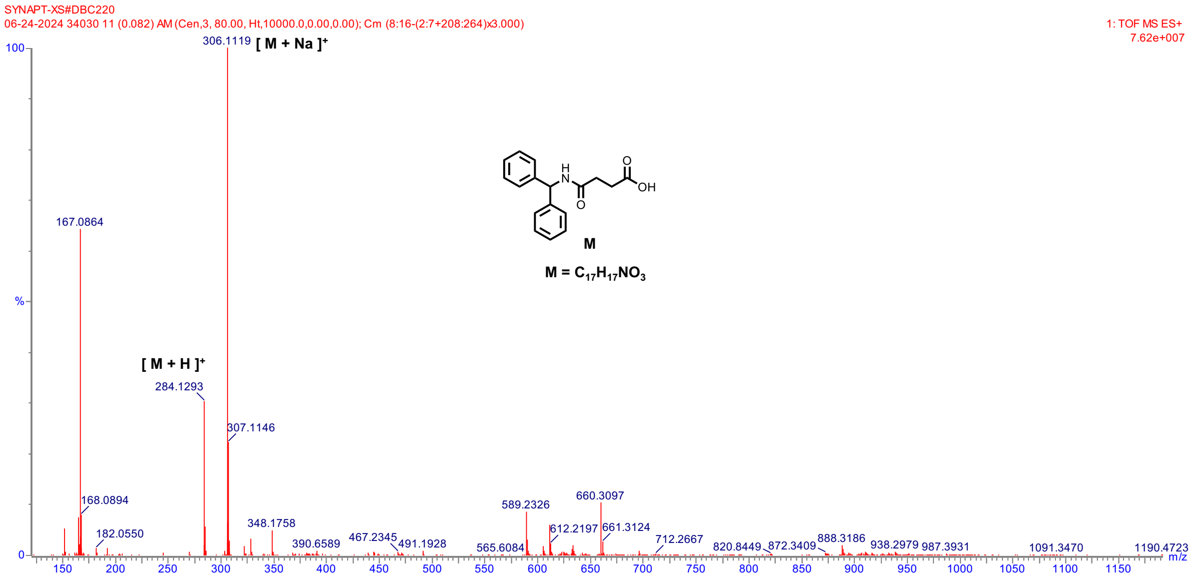

Figure S65. Mass spectrum of compound **12**.

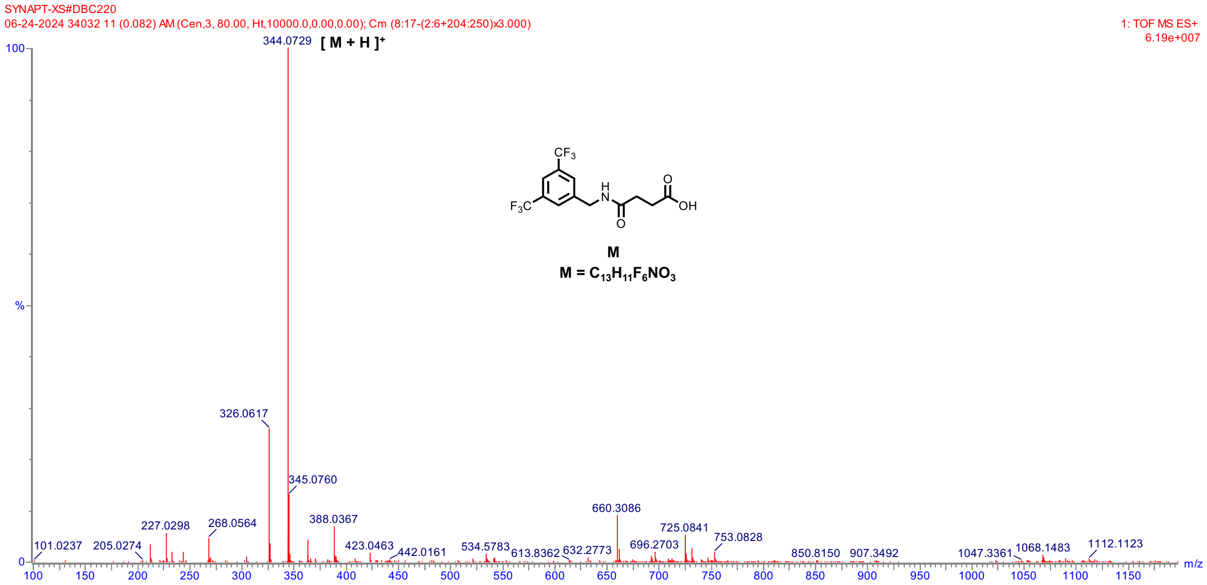

Figure S66. Mass spectrum of compound **14**.

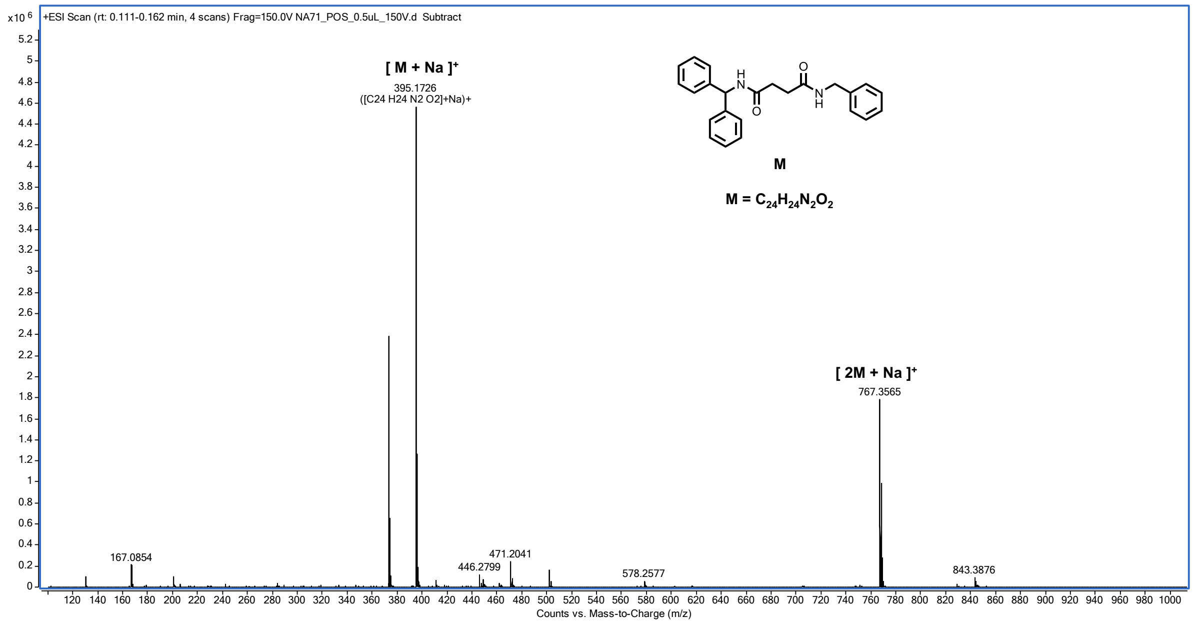

Figure S67. Mass spectrum of compound **1**.

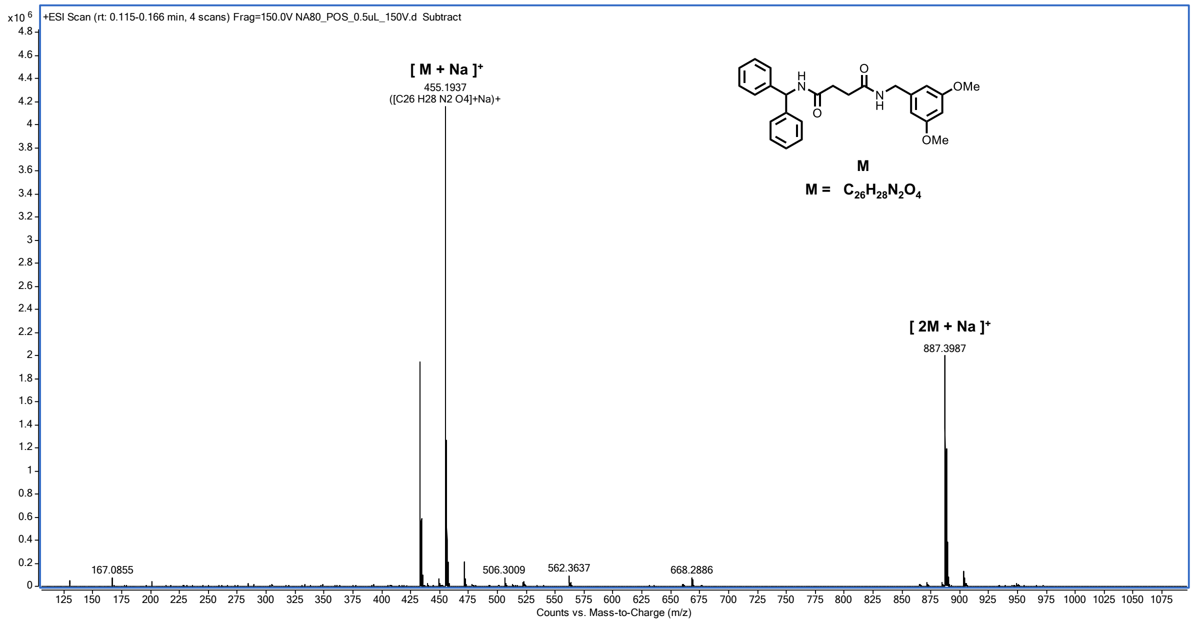

Figure S68. Mass spectrum of compound **2**.

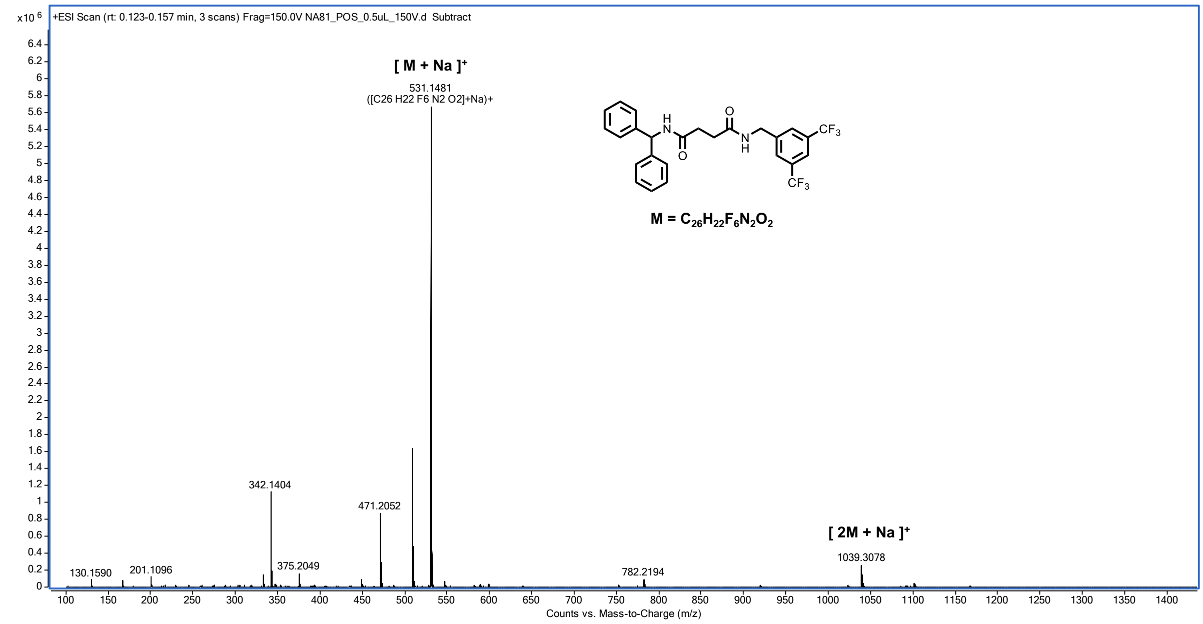

Figure S69. Mass spectrum of compound **3**.

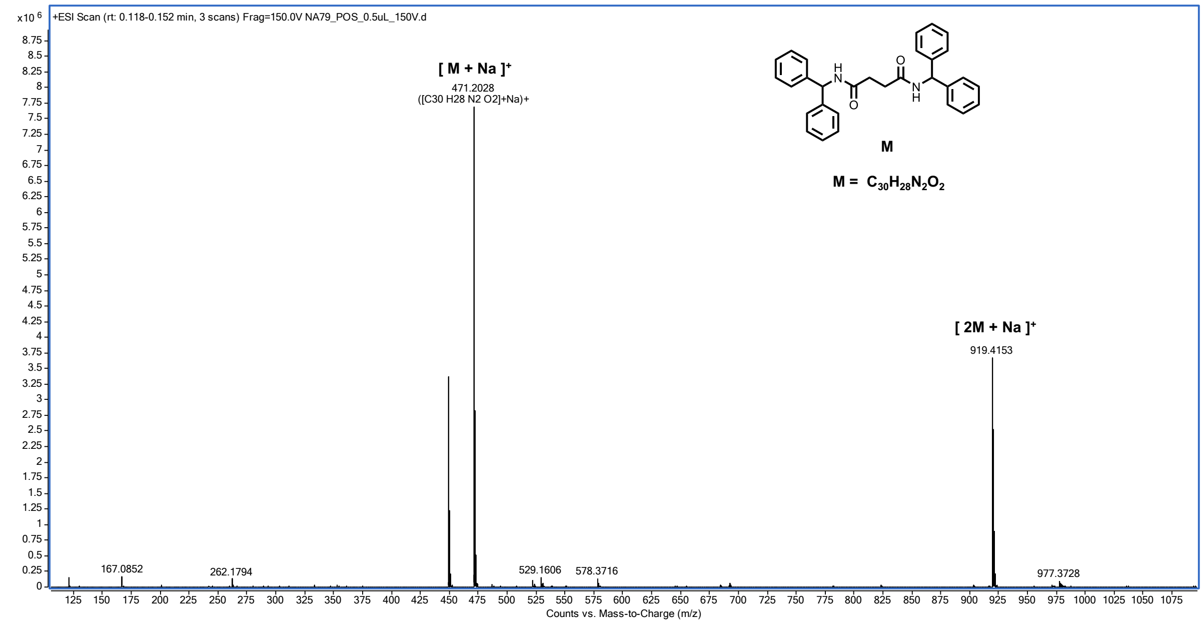

Figure S70. Mass spectrum of compound **4**.

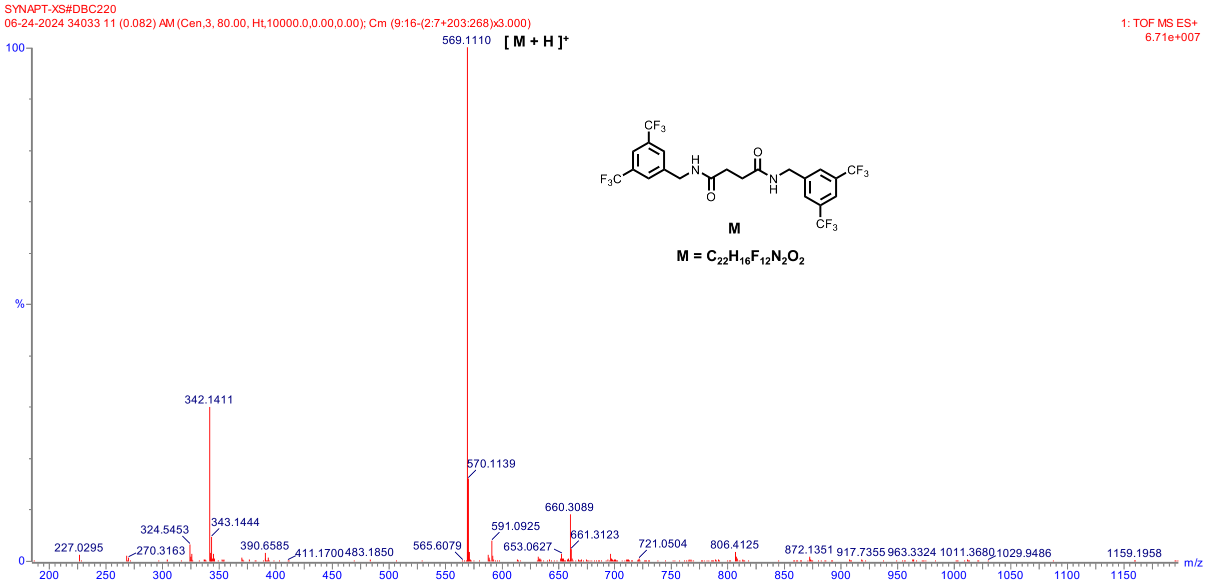

Figure S71. Mass spectrum of compound **5**.

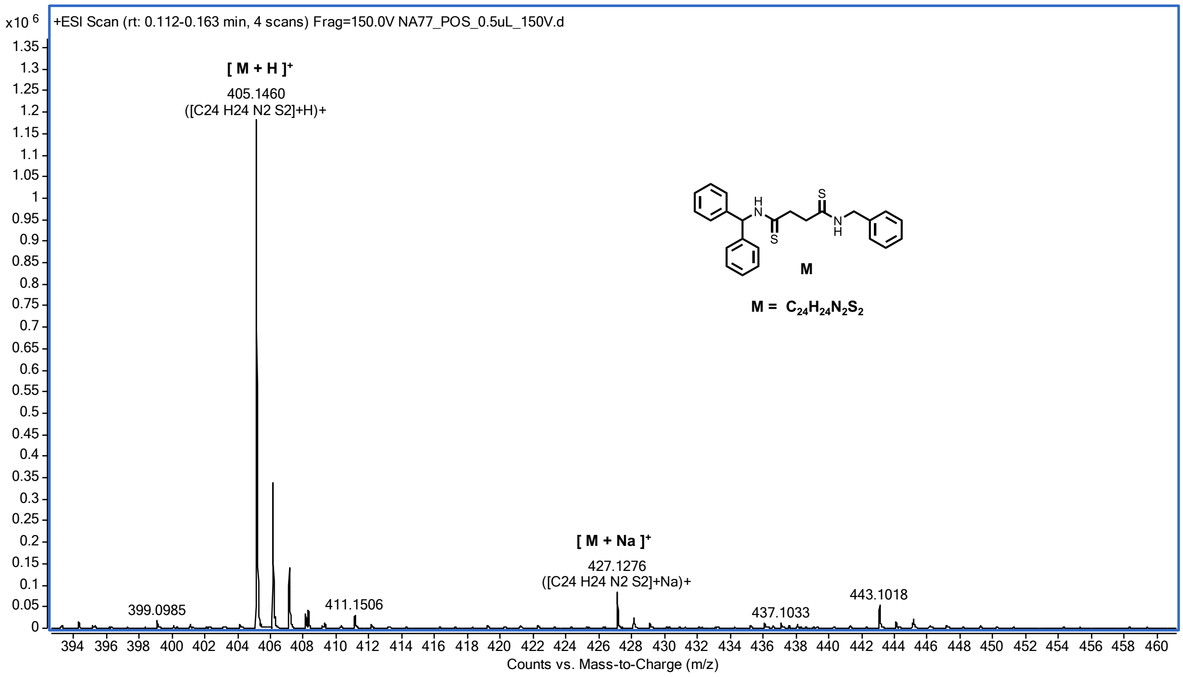

Figure S72. Mass spectrum of compound **6**.

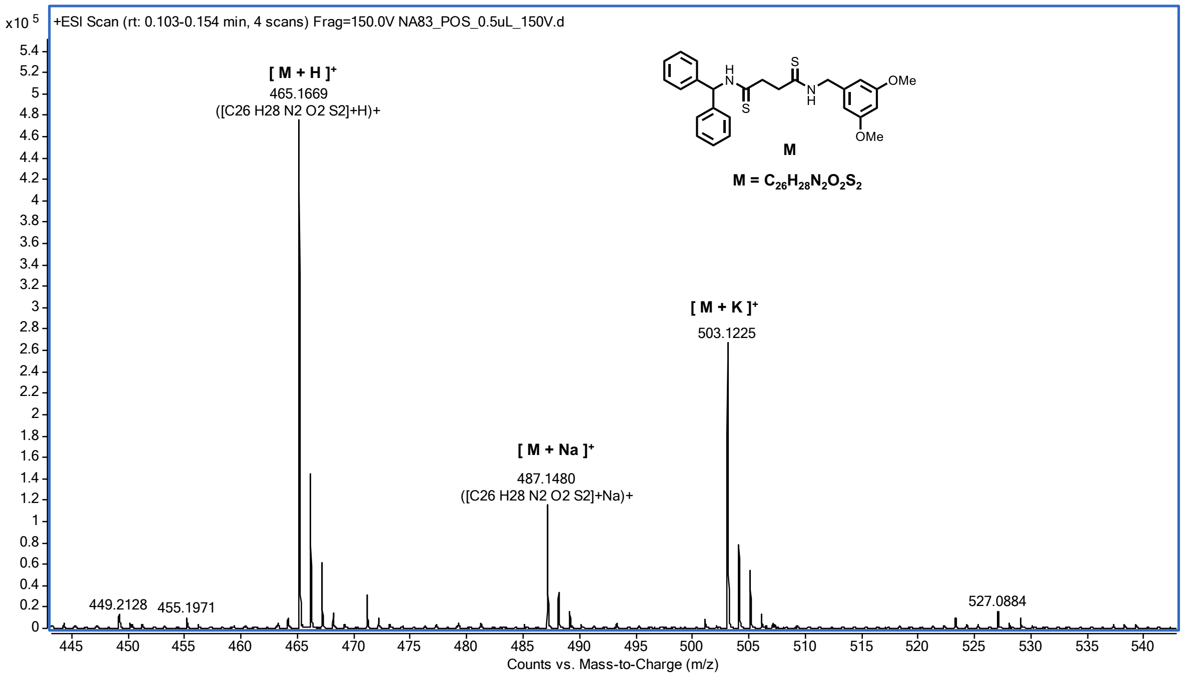

Figure S73. Mass spectrum of compound **7**.

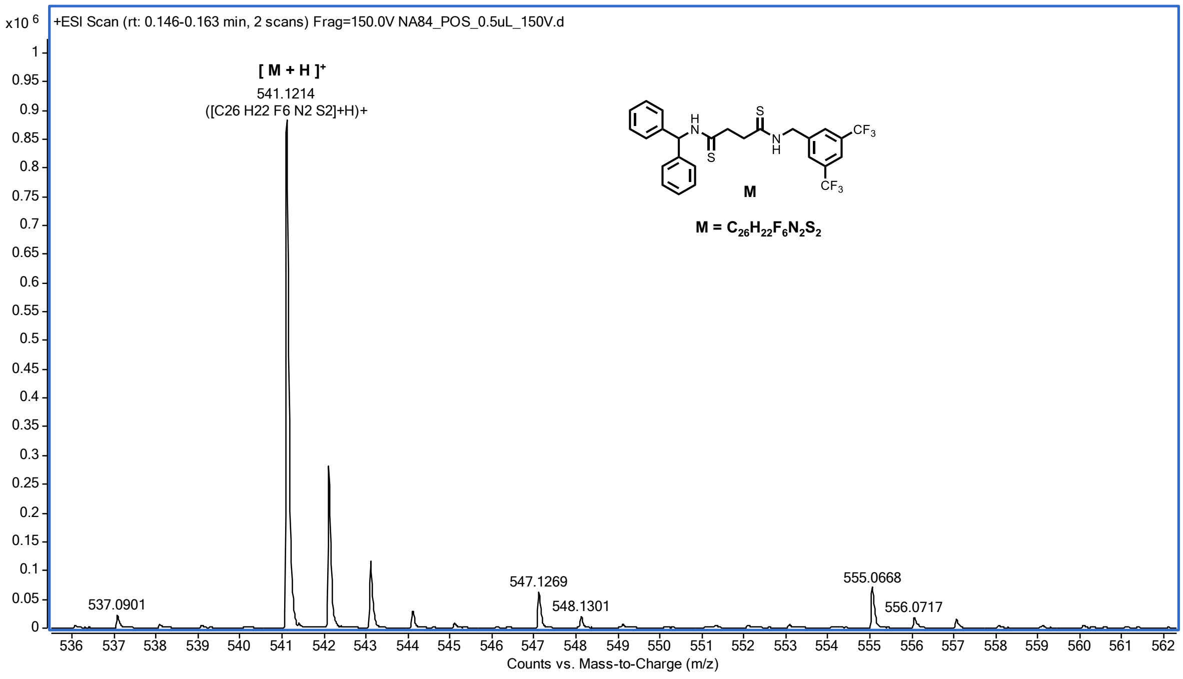

Figure S74. Mass spectrum of compound **8**.

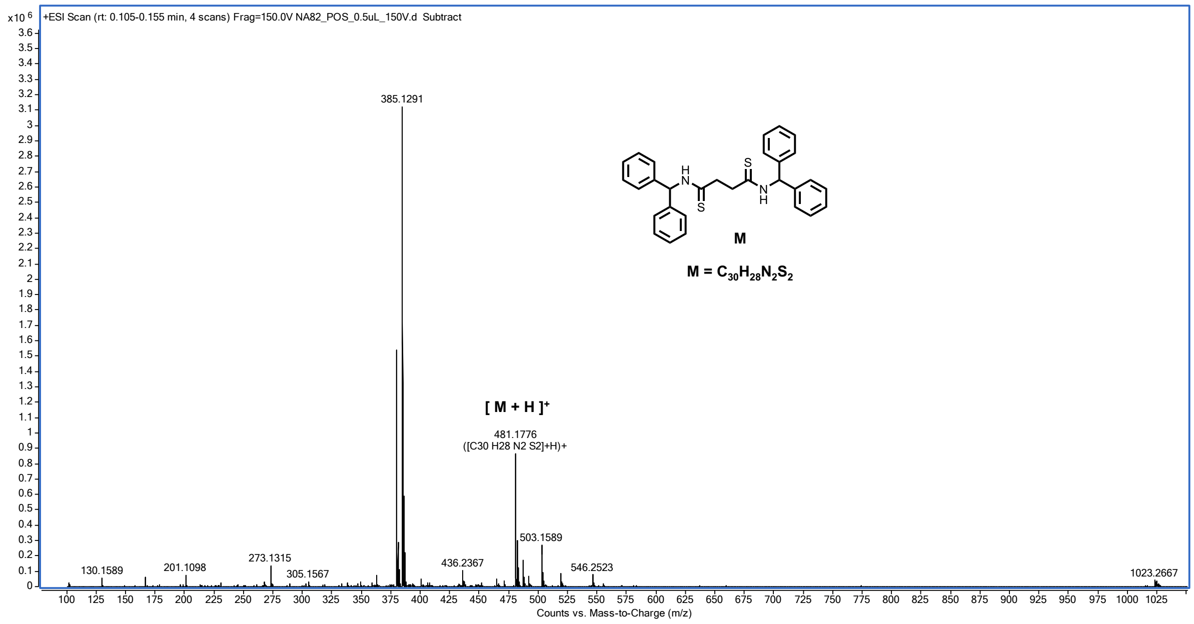

Figure S75. Mass spectrum of compound **9**.

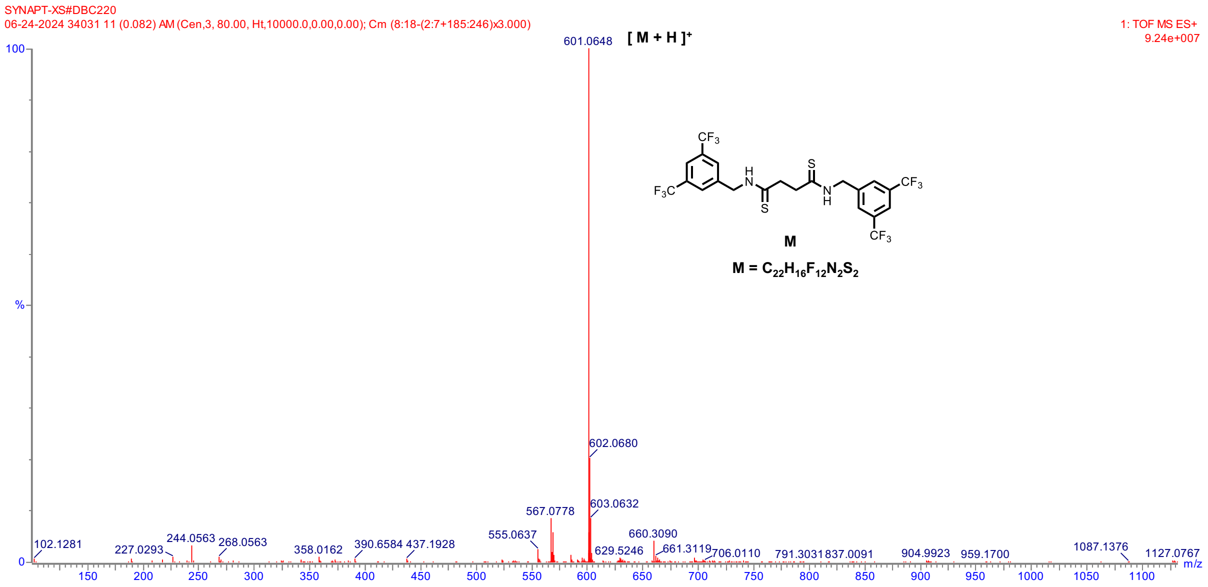

Figure S76. Mass spectrum of compound **10**.

S14 References

1. C. Gao, Z.-L. Luan, Q. Zhang, S. Yang, S.-J. Rao, D.-H. Qu and H. Tian, *Org. Lett.*, 2017, **19**, 1618–1621.
2. P. Thordarson, *Chem. Soc. Rev.*, 2011, **40**, 1305–1323.
3. D. B. Hibbert and P. Thordarson, *Chem. Commun.*, 2016, **52**, 12792–12805.
4. N. Akhtar, U. N. K. Conthagamage, S. P. Bucher, Z. A. Abdulsalam, M. L. Davis, W. N. Beavers and V. García-López, *Mater. Adv.*, 2024, **5**, 8534–8545.
5. G. te Velde, F. M. Bickelhaupt, E. J. Baerends, C. Fonseca Guerra, S. J. A. van Gisbergen, J. G. Snijders and T. Ziegler, *J. Comput. Chem.*, 2001, **22**, 931–967.

6. C. Fonseca Guerra, J. G. Snijders, G. te Velde and E. J. Baerends, *Theor Chem Acc*, 1998, **99**, 391–403.
7. Vrije Universiteit: Amsterdam, the Netherlands, ADF, https://www.scm.com/amsterdam-modeling-suite/adf/, (accessed September 16, 2025).
8. S. Grimme, J. Antony, S. Ehrlich and H. Krieg, *J. Chem. Phys.*, 2010, **132**, 154104.
9. S. Grimme, S. Ehrlich and L. Goerigk, *J. Comput. Chem.*, 2011, **32**, 1456–1465.
10. E. van Lenthe, E. J. Baerends and J. G. Snijders, *J. Chem. Phys.*, 1994, **101**, 9783–9792.
11. C. Nieuwland, F. Zaccaria and C. Fonseca Guerra, *Phys. Chem. Chem. Phys.*, 2020, **22**, 21108–21118.
12. P. Vermeeren, L. P. Wolters, G. Paragi and C. Fonseca Guerra, *ChemPlusChem*, 2021, **86**, 812–819.
13. E. Van Lenthe and E. J. Baerends, *J. Comp. Chem.*, 2003, **24**, 1142–1156.
14. M. Franchini, P. H. T. Philipsen, E. van Lenthe and L. Visscher, *J. Chem. Theory Comput.*, 2014, **10**, 1994–2004.
15. A. D. Becke, *J. Chem. Phys.*, 1988, **88**, 2547–2553.
16. P. Pracht, F. Bohle and S. Grimme, *Phys. Chem. Chem. Phys.*, 2020, **22**, 7169–7192.
17. A. Klamt, *J. Phys. Chem.*, 1995, **99**, 2224–2235.
18. A. Klamt and G. Schüürmann, *J. Chem. Soc., Perkin Trans. 2*, 1993, 799–805.
19. A. Klamt and V. Jonas, *J. Chem. Phys.*, 1996, **105**, 9972–9981.
20. C. C. Pye and T. Ziegler, *Theor. Chem. Acc.*, 1999, **101**, 396–408.
21. N. L. Allinger, X. Zhou and J. Bergsma, *Journal of Molecular Structure: THEOCHEM*, 1994, **312**, 69–83.
22. E. S. Böes, P. R. Livotto and H. Stassen, *Chemical Physics*, 2006, **331**, 142–158.
23. F. Zaccaria, G. Paragi and C. F. Guerra, *Phys. Chem. Chem. Phys.*, 2016, **18**, 20895–20904.
24. H. Jacobsen, A. Bérces, D. P. Swerhone and T. Ziegler, *Comput. Phys. Commun.*, 1997, **100**, 263–276.
25. A. Bérces, R. M. Dickson, L. Fan, H. Jacobsen, D. Swerhone and T. Ziegler, *Comput. Phys. Commun.*, 1997, **100**, 247–262.
26. S. K. Wolff, *i*, 2005, **104**, 645–659.
27. S. Grimme, *Chemistry – A European Journal*, 2012, **18**, 9955–9964.
28. W.-J. van Zeist and F. M. Bickelhaupt, *Org. Biomol. Chem.*, 2010, **8**, 3118–3127.
29. P. Vermeeren, S. C. C. van der Lubbe, C. Fonseca Guerra, F. M. Bickelhaupt and T. A. Hamlin, *Nat. Protoc.*, 2020, **15**, 649–667.
30. P. Vermeeren, T. A. Hamlin and F. Matthias Bickelhaupt, *Chem. Commun.*, DOI:10.1039/D1CC02042K.
31. F. M. Bickelhaupt, *J. Comput. Chem.*, 1999, **20**, 114–128.
32. F. Zaccaria, S. C. C. van der Lubbe, C. Nieuwland, T. A. Hamlin and C. Fonseca Guerra, *ChemPhysChem*, 2021, **22**, 2286–2296.
33. F. M. Bickelhaupt and E. J. Baerends, in *Reviews in Computational Chemistry*, John Wiley & Sons, Ltd, 2000, pp. 1–86.
34. T. A. Hamlin, P. Vermeeren, C. Fonseca Guerra and F. M. Bickelhaupt, in *Complementary Bonding Analysis*, ed. S. Grabowsky, De Gruyter, 2021, pp. 199–212.
35. C. Fonseca Guerra, J.-W. Handgraaf, E. J. Baerends and F. M. Bickelhaupt, *J. Comput. Chem.*, 2004, **25**, 189–210.

36. T. A. Albright, J. K. Burdett and M.-H. Whangbo, *Orbital Interactions in Chemistry*, John Wiley & Sons, 2013.
